# Risk factors for Epstein Barr virus-associated cancers: a systematic review, critical appraisal, and mapping of the epidemiological evidence

**DOI:** 10.7189/jogh.10.010405

**Published:** 2020-06

**Authors:** Deniz Bakkalci, Yumeng Jia, Joanne R Winter, Joanna EA Lewis, Graham S. Taylor, Helen R Stagg

**Affiliations:** 1 Institute for Global Health, University College London, London, UK; 2 Department of Infectious Disease Epidemiology, Imperial College London, London, UK; 3 MRC Centre for Global Infectious Disease Analysis, Department of Infectious Disease Epidemiology, Imperial College London, London, UK; 4 College of Medical and Dental Sciences, University of Birmingham, Birmingham, UK; 5 Usher Institute, University of Edinburgh, Edinburgh, UK; * Joint first authors, listed alphabetically.; † Joint senior authors.

## Abstract

**Background:**

Epstein Barr Virus (EBV) infects 90%-95% of all adults globally and causes ~ 1% of all cancers. Differing proportions of Burkitt’s lymphoma (BL), gastric carcinoma (GC), Hodgkin’s lymphoma (HL) and nasopharyngeal carcinoma (NPC) are associated with EBV. We sought to systematically review the global epidemiological evidence for risk factors that (in addition to EBV) contribute to the development of the EBV-associated forms of these cancers, assess the quality of the evidence, and compare and contrast the cancers.

**Methods:**

MEDLINE, Embase and Web of Science were searched for studies of risk factors for EBV-associated BL, GC, HL and NPC without language or temporal restrictions. Studies were excluded if there was no cancer-free comparator group or where analyses of risk factors were inadequately documented. After screening and reference list searching, data were extracted into standardised spreadsheets and quality assessed. Due to heterogeneity, a narrative synthesis was undertaken.

**Results:**

9916 hits were retrieved. 271 papers were retained: two BL, 24 HL, one GC and 244 NPC. The majority of studies were from China, North America and Western Europe. Risk factors were categorised as dietary, environmental/non-dietary, human genetic, and infection and clinical. Anti-EBV antibody load was associated with EBV-associated GC and BL. Although the evidence could be inconsistent, HLA-A alleles, smoking, infectious mononucleosis and potentially other infections were risk factors for EBV-associated HL. Rancid dairy products; anti-EBV antibody and EBV DNA load; history of chronic ear, nose and/or throat conditions; herbal medicine use; family history; and human genetics were risk factors for NPC. Fresh fruit and vegetable and tea consumption may be protective against NPC.

**Conclusions:**

Many epidemiological studies of risk factors in addition to EBV for the EBV-associated forms of BL, GC, HL and NPC have been undertaken, but there is a dearth of evidence for GC and BL. Available evidence is of variable quality. The aetiology of EBV-associated cancers likely results from a complex intersection of genetic, clinical, environmental and dietary factors, which is difficult to assess with observational studies. Large, carefully designed, studies need to be strategically undertaken to harmonise and clarify the evidence.

**Registration:**

PROSPERO CRD42017059806.

Epstein Barr Virus (EBV) is a herpesvirus estimated to infect 90%-95% of all humans [1]. Infection is lifelong and in low-income settings occurs in childhood; in higher income settings it may also occur in childhood but is often delayed until late adolescence or early adulthood. In a minority of people infection is associated with the development of cancer [2]. Indeed, Epstein, Achong and Barr’s work describing the presence of EBV in a cultured Burkitt’s lymphoma (BL) cell line was the scientific foundation implicating the first known human tumour virus [3]. EBV is now associated with 1% of global cancers, which are mostly lymphomas and carcinomas; approximately 140 000 people die of EBV-associated cancers each year [4,5].

Despite decades of intensive research, the aetiology of EBV-linked cancers remains unclear. As only a fraction of EBV-infected individuals develop cancer, other factors must also be influential. Several reviews have been undertaken of the biological and epidemiological evidence for the association between EBV and different cancers [5-16], but none have systematically collated the epidemiological evidence for additional risk factors beyond EBV infection, or assessed the quality of the presented evidence. Pragmatically, such an undertaking requires a focus on a small number of contrasting cancer types that have strong evidence of a causal relationship with EBV, are of significant burden and/or have globally disparate distributions, such as BL, Hodgkin’s lymphoma (HL), gastric carcinoma (GC), and nasopharyngeal carcinoma (NPC).

In 2016, there were approximately 461 000 incident cases of non-Hodgkin’s lymphoma (NHL) globally [17]. BL is a highly aggressive and fast-growing NHL classified in three forms: endemic, sporadic and AIDS-associated [5]. The endemic form is thought to have EBV present in more than 95% of tumours; this decreases to 15%-88% for sporadic tumours and 30%-40% for those that are AIDS-associated [5]. Endemic BL is generally a paediatric condition of the jaw found in equatorial Africa and Papua New Guinea which has been strongly linked to malaria [5]. Sporadic BL is associated largely with white populations, across a wider age range and disease of the abdomen [5]. All types of BL are more common in males than females [5].

HL is a lymphoma characterised by the presence of Hodgkin and Reed-Sternberg cells which has various histological appearances. There were an estimated 73 000 incident cases globally in 2016 [17]. 40%-50% are thought to be associated with EBV, but the degree of association is highly population-dependent; EBV positive HL as a percentage of all HL is highest (90%-100%) in lower income countries [18-20]. Incidence rates of this form of cancer peak in children and individuals over 70 years of age, are more common in non-white ethnic groups, and in males than females [5].

With 830 000 deaths and 1.2 million cases globally (2016), GC is a major cause of morbidity and mortality [17]. Around 95% of GC is gastric adenocarcinoma; approximately 10% of this is EBV-associated, with variation between populations [5]. Due to the commonality of GC, EBV-associated GC is likely to be the most common EBV-associated malignancy, but is poorly studied [5]. The cancer is associated with being male; for men prevalence decreases with age [21].

96 000 cases of cancer of the nasopharynx occurred in 2016 [17]. NPC forms the majority of this burden and occurs in the epithelial cells of the nasopharynx. Tumours are classified depending upon the level of differentiation in these cells: type I (squamous), type II (non-keratinising), type III (undifferentiated). EBV is strongly associated with types II and III and more controversially with type I [22]. NPC incidence is geographically extremely variable, with the disease associated with northern Africa and southeast Asia, particularly southern China and East Malaysia [5]. The disease is more common in males; age associations vary between settings.

Given the global importance of EBV-associated BL, HL, GC and NPC, their contrasting features yet common link with EBV, and their complex but unclear aetiology, we undertook a systematic review to map the current global epidemiological evidence base of risk factors for the EBV-associated forms of these tumours in addition to EBV itself. We present an evaluative narrative summary of that evidence, a quality assessment, and an appraisal of the critical gaps in the literature.

## METHODS

### Search strategy

MEDLINE (United States National Library of Medicine, Bethesda MD, United States of America; through Ovid [Wolter Kluwer, Alphen aan den Rijn, The Netherlands]), Embase (Elsevier, Amsterdam, The Netherlands; through Ovid) and the Web of Science (Clarivate Analytics, Philadelphia, PA, United States of America) were searched for studies of risk factors for a) EBV-associated HL and BL and b) EBV-associated NPC and GC in June to August 2017 (Appendix S1 in the **Online Supplementary Document**). Search strategies were compiled for MEDLINE and then adapted for the other databases. Reference lists of included papers and review articles were also searched (‘snowballing’). This review was registered on PROSPERO as CRD42017059806.

### Study selection

Inclusion criteria:

- Epidemiological studies (observational and interventional) of risk factors for EBV-associated HL, BL, NPC and/or GC with extractable data and a cancer-free comparator population.
- Human studies.
- No date restrictions.
- No population restrictions.
- No language restrictions.

Exclusion criteria:

- Animal studies.
- Studies of risk factors for EBV infection, not EBV-associated cancers.
- Studies where the tumours were not proven to be EBV positive, unless for NPC, where EBV is thought to be associated with 95% of tumours [23].
- Comparator population had cancer or pre-cancerous lesions (including both EBV-negative HL/BL/GC/NPC and other forms of cancer).
- Gene/protein expression or genetic (human or EBV) studies of samples taken from cancer tissues, due to the potential for post-tumour mutations.
- Genetics studies where samples were taken from different bodily sites for the cancer and comparator groups.
- EBV genetic and anti-EBV antibody expression studies where samples were taken cross-sectionally from cancer patients, due to the potential for tumour-induced expression, or where antibody load was not documented.

Hits were screened at the title, abstract and full text stages by three reviewers per cancer, with at least a 10% overlap. Disagreements were resolved by an independent reviewer. Data extraction was undertaken by the same three reviewers, with at least 10% overlap. Discrepancies were resolved by consensus.

### Data extraction

Data were extracted into a pre-designed spreadsheet which included data on the study design, population and risk factors assessed for EBV-associated cancers.

### Quality assessment

The quality of included studies was assessed using a checklist adapted from Downs and Black [24], as per the guidance issued by Deeks et al. [25]. Quality assessment was made for the main risk factor(s) of interest; if multiple risk factors were documented the most conservative score was taken. The minimum sample size required to detect a relative increase in cancer of 50% from a statistically conservative baseline of 50% among the unexposed was calculated at different powers (with a significance level of 5%) using the Fleiss method within Epi Info. Different thresholds were set for cohort/cross-sectional studies and (unmatched) case-control studies. Conservatively, a ratio of one to one between exposure strata was assumed, as well as only two strata. This criteria was scored from 0 (<70% power) to 5 (>99% power). We pragmatically defined a minimal confounder set as age, sex and ethnicity. Three reviewers undertook the assessment, with at least 10% overlap. Discrepancies were resolved by consensus.

### Data synthesis

Due to between-study heterogeneity, the findings of the included publications were narratively synthesised by theme.

### Ethics approval

As a systematic review of previously published data, this work did not require ethical approval.

## Results

### Search results and included studies

**Figure 1:**
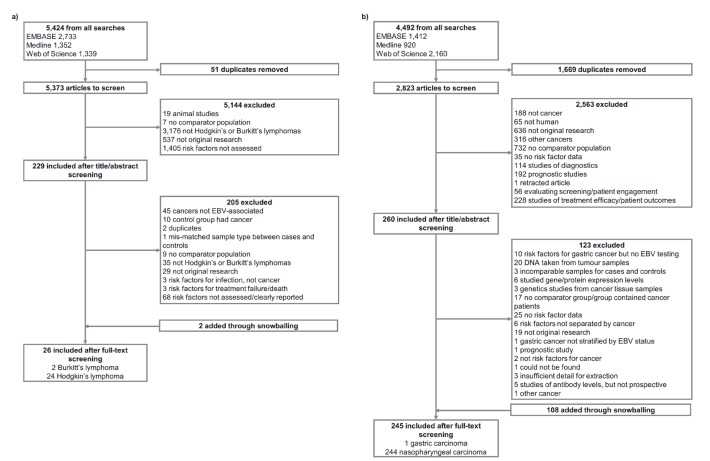
Flowcharts of extraction for systematic review. **Panel A.** EBV-associated Hodgkin’s and Burkitt’s lymphomas. **Panel B.** EBV-associated nasopharyngeal carcinoma and gastric carcinoma. EBV – Epstein Barr virus.

**Figure 2:**
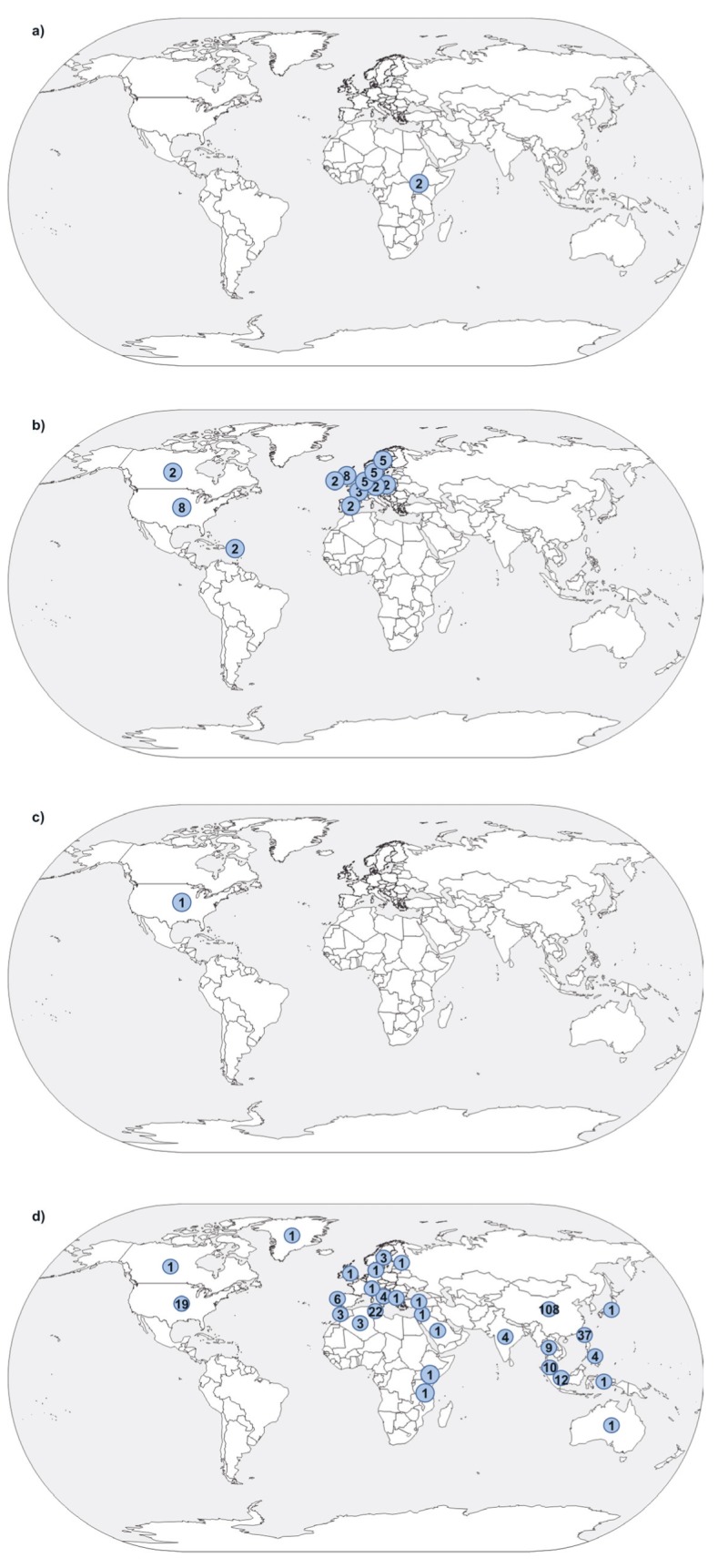
Global distribution of extracted publications. Dots represent the number of publications reporting data from a particular setting, where reported. One publication may have studied participants in multiple settings. Map taken from http://www.freeworldmaps.net/outline/maps/world-map-outline.gif. **Panel A.** Burkitt’s lymphoma. **Panel B.** Hodgkin’s lymphoma. **Panel C.** gastric carcinoma. **Panel D.** nasopharyngeal carcinoma (excluding the global study by Shield et al.) [26].

After de-duplication, 5373 hits were retrieved by the lymphomas search and 2823 by the carcinomas search (**Figure 1**). This was reduced to 229 and 260 hits, respectively, after title and abstract screening. 271 papers were extracted, including those identified through snowballing: two on EBV-associated BL, 24 on EBV-associated HL, one on EBV-associated GC and 244 on NPC (Table S1 in the **Online Supplementary Document**). All BL, HL and GC publications used case-control designs (although one of the HL papers also contained a reconstructed cohort) and 227/244 (93.0%) of those for NPC. Papers analysed populations ranging in size between 11 and hundreds of thousands (longitudinal studies). The majority of NPC analyses were undertaken in China, GC and HL papers in Europe and the Americas, and BL papers in Africa (**Figure 2**).

### Quality assessment of included studies

In the overall quality assessment, 32/271 (11.8%) of publications were very under-powered, with a score of zero or one, ie, less than 80% power (Table S2 in the **Online Supplementary Document**). An assessment of power could not be undertaken for eight publications. All of the BL and GC publications scored zero for power. 15/24 (62.5%) of HL publications and 208/244 (85.2%) NPC publications had a sample size sufficient for 90% power or above.

171/271 (63.1%) analyses were undertaken using appropriate statistical methods eg, conditional regression for individually matched studies; 65/271 (24.0%) adjusted for the minimal confounder set. Across the publications, none were thought to be at high risk for observer bias for the outcome, but the potential for recall bias was higher (85/271, 31.4%), with exceptions being studies of genetic risk factors.

### Risk factors for Burkitt’s lymphoma

Two publications met our inclusion criteria for EBV-associated BL. Both were undertaken on the same population in Uganda in the 1970s. They examined infection and clinical factors, specifically anti-EBV antibody load prior to cancer diagnosis (anti-viral capsid antigen [VCA], anti-early antigen [EA] and anti-EBV nuclear antigen [EBNA]) [27,28]. Only levels of anti-VCA were found to be associated with the development of BL. Both had small sample sizes and largely utilised descriptive statistics.

### Risk factors for Hodgkin’s lymphoma

Among the 24 EBV-associated HL publications, three examined dietary risk factors, 11 infection and clinical factors, eight human genetic factors, and five environmental and non-diet lifestyle. Within these publications, 21 represented eight sets of overlapping or identical study populations (and one did not have enough information to classify) [29]: one set from Denmark and Sweden [30-34]; one from the Netherlands [30,34-37]; three from the UK (England [38,39], England [30,34,40,41], England and Scotland) [30,34,36,41,42]; two from the USA (Connecticut and Massachusetts [43-45]; California [46-48]); and one from Canada, Puerto Rico and the USA [49,50].

Of the papers partially or totally examining diet as a risk factor for HL (**Table 1**), one looked at dietary patterns [43], one alcohol [38], and one dietary fats [44]. None of the papers found any statistically associated risk factors. Two of these publications used substantially overlapping study populations [43,44]. Due to the retrospective collection of dietary information, recall bias may have been an issue.

**Table 1:** Summary of dietary factors associated with Hodgkin’s lymphoma

Potential risk factor	Summary of results
Alcohol	No association [38]
Dietary patterns (“vegetable”, “high meat”, “fruit/low-fat dairy”, “desserts/sweets”)	No association [43]
Dietary fats (total fat intake; fat subtypes)	No association [44]

Within the infection and clinical factors publications, three from Europe provided an indication that personal infectious mononucleosis (IM) may be a risk factor for HL (**Table 2**) [32,40,42]. Although the two publications from the Americas had a high degree of uncertainty (confidence intervals (CI) crossed the null), the reported effect estimates were somewhat suggestive of risk for either personal or familial IM [47,49]. Importantly, all exposure data was self-reported.

**Table 2:** Summary of infection and clinical factors associated with Hodgkin’s lymphoma

Potential risk factor	Summary of results
**Infection:**	
Childhood infections (not IM)	Two or more infections (measles, mumps, chicken pox, pertussis, rubella) associated with reduced risk [40]
	Increased risk associated with Strep or sore throat/scarlet fever/tonsillitis, infections in siblings. Other personal infections also considered [49]
	Measles, mumps, rubella (as a single variable, but not individually) – potentially protective [47]
	Measles, mumps, rubella in older adult patients and chicken pox across all age groups – no association found [47]
IM (personal)	Personal IM associated with increased risk (across all ages and in younger adults alone; association not seen in older adults alone) [32]
	Personal IM associated with increased risk [40]
	Personal IM associated with increased risk [42]
	Personal IM – no association found [47,49]
IM (familial)	Familial IM – no association found [42,47]
	IM in siblings – no association found [49]
CMV	CMV seropositivity associated with increased risk [51]
**Other clinical:**	
Autoimmune diseases and allergies	Rheumatoid arthritis associated with increased risk. Other autoimmune and allergic conditions also examined [33]
	Autoimmune conditions in parents associated with increased risk. Personal autoimmune or allergic conditions, autoimmune conditions and allergies in siblings, allergies in parents – no association [49]
BMI, weight, height	No association (BMI) [39]
	Higher weight and BMI associated with protection [45]
	No association [48]
Physical activity	Participating in (strenuous) physical activity as an adult associated with protection [47]
**Family history of cancer**	Earlier age of cancer onset among family members may be associated with an increased risk. Different types of tumour examined [48]

BMI – body mass index, CMV – cytomegalovirus, IM – infectious mononucleosis

Three publications examined the impact of childhood infections. Using a combined variable, Glaser et al. found that measles, mumps or rubella may be protective against HL diagnosed in younger patients aged 19-44 years [47]. When these factors were considered individually CIs crossed the null, but the effect estimates tended in the same direction. In older adolescents and young adults, Alexander et al. demonstrated that two or more infections (measles, mumps, chicken pox, pertussis) were protective without examining each infection individually [40]. Within Linabery et al. (children and adolescents), the direction of effect was protective for mumps, neutral for measles and in favour of risk for rubella (all with CIs that crossed the null), but Strep or sore throat/Scarlet fever/Tonsillitis as a combined variable was associated with greater HL risk and likely contributed strongly to overall findings for infections [49]. Again, all exposure data was self-reported. Another publication documented a potential association with cytomegalovirus (CMV) serostatus, but minimal information was available as it was a conference abstract [51].

Two papers examined the impact of autoimmune and allergic conditions on the risk of HL [33,49]. Although each found specific risk factors among the autoimmune conditions (eg, personal rheumatoid arthritis, parental autoimmune conditions), the evidence was not consistent. Neither scored higher for quality than the other.

A second publication by Linabery et al., using an overlapping population to their infection, autoimmune and allergy study [49], examined the impact of family cancer history, but found no clear associations [50].

Body mass index (BMI), weight, height and measures of physical activity were examined as risk factors by three publications [39,45,48]. Physical activity as an adult was associated with protection from HL. Two papers documented no BMI association and one found that higher BMI/weight was protective. The direction of effect was not associated with the quality of the evidence.

Of the eight publications that examined the human genetic factors associated with EBV-associated HL, six focussed on human leukocyte antigens (HLA), with one additionally looking at tumour necrosis factor (TNF) α and β (**Table 3**) [29,30,35-37,41]. One examined Killer-cell immunoglobulin-like receptors (KIRs) [52] and one was a general genetics publication [34].

**Table 3:** Summary of human genetic factors associated with Hodgkin’s lymphoma

Potential risk factor	Summary of results
**HLA and associated genes:**	
*HLA-A*	A*01 associated with increased risk, A*02 with decreased risk [36]
	A*01:01 associated with increased risk [41]
*HLA-B*	B*08:01 associated with increased risk [41]
	B*07 and B*08 no association [29]
*HLA* region	Locus D6S265 allele 126 and locus D6S510 allele 284 heterozygotes and homozygotes (both HLA class I) associated with HL risk in a classic association analysis. (Other, weaker, associations also found.) Association lost when haplotype sharing statistic analysed [35]. Later narrowed down as above [36].
	HLA class I associated through seven SNPs – rs2530388, rs3823352, rs2256543, rs4713276, rs2523972, rs6457110, rs2517749 [7^6^]. Later narrowed down as above [36].
	C*07:01 and DRB1*03:01 associated with increased risk [41]
	Genome-wide association study: rs6904029 (HCG9) associated with decreased risk or rs2734986 (HLA-G) associated with increased risk [34]
	rs6457715, near HLA-DPB1, associated with increased risk [30]
**Cytokines and chemokines:**	
*TNFA*, *TNFB*	No association [35]
**Other immune-related:**	
*KIR*	No clear association [52]

HL – Hodgkin’s lymphoma, HLA – human leukocyte antigen, KIR – killer-cell immunoglobulin-like receptors, SNP – single nucleotide polymorphism, TNF – tumour necrosis factor

Among the HLA papers, the evidence was balanced in favour of a *HLA-A* association, particularly an increased risk with the HLA-A*01 serotype group. Three publications represented a gradual narrowing down to A*01 [35-37]. For B alleles, the evidence was contradictory. Various other alleles and loci were also associated.

Of the publications examining environmental and non-diet lifestyle factors, three looked at smoking as a risk factor for HL (**Table 4**) [31,38,46]. All three provided evidence supportive of an association between current smoking and HL (and two of ever having smoked and HL), including from Willet et al., which had previously found no association with alcohol.[38] More detailed breakdowns of smoking status were less clear, however. There was a risk of recall bias in all of the analyses and only one adjusted for all of our *a priori* confounders, but analysis choices were deemed appropriate throughout.

**Table 4:** Summary of environmental and non-diet lifestyle factors associated with Hodgkin’s lymphoma

Potential risk factor	Summary of results
Smoking	Having ever smoked and being a current smoker associated with increased risk [38]
	Having ever smoked and current smoking associated [31]
	Current smoking associated [46]
	Years smoked, pack years and years since stopped smoking – no association [38]
	Age at initiation of smoking, duration, intensity, cumulative exposure, time since cessation – not associated [31]
	Having ever smoked, intensity, duration, age at initiation, years since cessation and childhood exposure – no association [46]
Childhood environmental factors	Numbers of younger siblings associated with protection [32]
	Being an older sibling potentially protective among younger adult patients [47]
	Number of older siblings – no association [32]
	Childhood household size no association[47]
	Bedroom sharing associated with reduced risk among younger adult patients [47]
	Persons per room, attendance at kindergarten, mother’s age at birth, personal and parental education levels – no association [32]
	Number of playmates – no association [47]

Within the Glaser et al. and Hjlagrim et al. IM publications, environmental factors during childhood were also documented [32,47]. Among younger adult patients only (19-44 years; other grouping 45-79 years) in Glaser et al., sharing a bedroom and being an older sibling was associated with reduced risk. Similarly, Hjalgrim et al. noted that having more younger siblings was protective across all ages, but when the population was broken down by age the association was only observed among those 18-44 years (ie, not in those 45-74 years old). These papers performed similarly in terms of quality to their previously documented counterparts.

### Risk factors for gastric carcinoma

One publication met our inclusion criteria for EBV-associated GC. In American men of Japanese ancestry, Levine et al. examined infection and clinical factors for GC, specifically anti-EBV antibody load prior to cancer being diagnosed (IgG and IgA anti-VCA, IgG anti-EA and IgG anti-EBNA) [53]. IgG anti-VCA was specifically found to be associated with EBV-positive GC vs non-cancer controls. Power was very low and the minimal confounder set was not adjusted for.

### Risk factors for nasopharyngeal carcinoma

Among the 244 NPC publications, 45 examined dietary risk factors, 50 infection and clinical factors, 158 human genetic factors, and 56 environmental and non-diet lifestyle. Publications from the same setting and time frame (thus with overlapping populations) were numerous. Two Coghill et al. publications were a conference abstract and a manuscript of the same analyses, thus the abstract is excluded from the following [54,55].

Among the dietary risk factors papers, there were a series of common factors that were assessed (**Table 5**). The first of these was alcohol (often in combination with smoking), where generally the evidence was not in favour of involvement [56,58-73].

**Table 5:** Summary of dietary factors associated with nasopharyngeal carcinoma

Potential risk factor	Summary of results
**Alcohol**	Risk factor [78] [S1,S2]
	Potentially associated [79] [S3]
	No overall association [56,58-73]
	Results inconclusive [S4]
	Result undocumented [S5]
**Dairy:**	
Rancid dairy products	Rancid butter risk factor [61,75]
	Rancid butter potential association[91]
Other dairy products	Milk protective [S5,S6]
	Milk no association [61,86]
	Butter no association [61]
	Eggs protective [61] [S6]
	Eggs no association [86]
	Salted (duck) eggs (particular time points) risk factor [79,83]
	No association [91]
**Fish and shellfish:**	
Salted fish	Risk factor [57,59,64,65,71,77-82]
	Inconclusive [83-85]
	No association [59,71,76,85-88]
Other preserved fish	Protective [75]
	Dried fish results inconclusive [83]
	No association [87]
	Fermented fish (sauce) – no association [60,72,84]
Other fish/shellfish	Shrimp protective [79]
	No association [87]
	Fresh fish protective [S6]
	Fresh fish potentially protective [86]
	Fresh fish and other seafood – no association [86]
	Deep sea fish protective [S5]
**Meat:**	
Smoked, cured, dried, salted preserved meat	Risk factor [58] [S1]
	Risk factor, but not consistent [75,82]
	No association [74,76,86-88]
	Quaddid risk factor [S7]
	Fermented pork no association [72]
	Salted meat no association [72]
Other meat	Fresh meat (pork/beef liver) risk factor [79]
	Red meat risk factor [68]
	Chicken risk factor [S7]
	Chicken protective [91]
	Fried meat – inconclusive association [61]
	Sheep’s tail fat no association [S7]
	Merguez, khelli no association [75]
	Fresh meat no association [86]
	Processed meat no association [S6]
	Sausage no association [72]
**Other salted products**	Salt-cured food risk factor [68]
	Salted vegetables risk factor (at least in adulthood) [79,82]
	Salted and dried tomatoes or salted or brined peppers risk factor [61]
	Other salty foods – inconclusive association [61]
	Salted vegetables no association [71]
	Salted mustard greens – inconclusive association [83]
	Salted roots no association [79]
**Other fermented and preserved foods**	Fermented pastes risk factor[89]
	Fermented black bean paste and fermented soy bean paste no association[83]
	Fermented foods no association [S1]
	Fermented soy bean products associated [92]
	Fermented soybean products no association [88]
	Fermented and salted vegetables no association [72]
	Preserved vegetables (potential) risk factor [57,65,72]
	Preserved plums risk factor[84,93]
	Preserved vegetables generally risk factor (although salted vegetables and picked Chinese cabbage protective) [93]
	Preserved vegetables no association [86,88]
	Preserved fruit no association [79]
	Pickled vegetables (and fungus on pickles) risk factor [S7]
	Mouldy bean curd no association[84]
**Vegetables, beans and fruit**	Chung choi not consistently associated, could be risk factor [77]
	Fresh fruits in childhood protective [81]
	Fresh fruit associated protective [61]
	Fresh fruit and vegetables protective [79]
	Fresh green vegetables protective, others no association [84]
	Fruit and vegetables protective [68] [S6]
	Leafy vegetables protective [85]
	Dark vegetables and fresh fruit protective [S5]
	Grapes protective[91,93]
	Non-preserved fruits and vegetables generally protective [93]
	Cooked vegetables and citrus fruits not consistently associated[75]
	Fresh fruit and vegetables (including green and leafy) no association [74,86]
	Servings per week of fruit and vegetables no association [88]
	Carrots no association[64,66,91]
**Other:**	
Coffee	No association [73,86] [S6]
Tea	Herbal tea risk factor [85]
	Green tea protective [86]
	Herbal tea protective [82]
	Protective [65,73] [S4,S6]
	Inconclusive association [61]
	Black, Oolong tea no association [86]
Slow cooked soup	Protective [65,82]
Dietary nutrients	Folate, vitamin B6, protective; vitamin B12, methionine no association [S8]
	Vitamin A, Vitamin C, tocopherol no association [86]
	Beta carotene and vitamin C no association [74]
Foods containing nitrosamines	Inconclusive association across all foods [88]
Preserves and condiments	In childhood risk factor [S7]
	Regular spicy sauce consumption generally risk factor [61]
	No association [75,91]
Other	Irregular meals risk factor [61]
	Adult diet on weaning risk factor [S7]
	Rancid sheep fat risk factor [75]
	Melon seeds risk factor [85]
	Sugary, dried or salted snacks, risk factor [61]
	Food additives risk factor [S9]
	White bread risk factor [S9]
	Lentils protective [91]
	Corn bread protective [S9]
	Margarine and olive oil protective [S9]
	M’hammas no association [S7]
	Potatoes no association [91]
	Grains no association [61]
	Monosodium glutamate no association [85]
	Non-dairy oils and fats no association, unless salted [61]
	Olives no association [75,87]
	Protein, fat, carbohydrate, sodium intake, soybean milk no association [86]
	Olives inconclusive [61]
	French fries – inconclusive association [61]
	General diet – results unclear [95]

*References marked with letter S are available in the **Online Supplementary Document**.

Salted fish was a second frequently analysed factor, with eleven publications determining it to be a risk factor [57,59,64,65,71,77-82], three with some (but not conclusive) evidence of association [83-85], and seven as not associated with NPC (although, in four of these, effect estimates trended in the risk factor direction) [60,72,76,86-89]. Smoked, cured or dried preserved meats had no consistent association with NPC (seven papers) [72,74,76,86-88]. Rancid dairy and other fats were considered to be a risk factor or to be potentially associated in all four of the analyses that included them [61,75,91]. Slow cooked soup was associated with protection against NPC in two [65,82].

Two papers were in agreement that preserved plums were a risk factor for NPC [84,93]. Broadly, fresh fruit and vegetables were deemed protective, as was the consumption of tea in various forms, including herbal.

The infection and clinical papers examined risk factors that fell into eight main categories-EBV serology/DNA load/genetics, other infections, medical history, medication, oral hygiene, T-cells, and family history of NPC or cancer (**Table 6**).

**Table 6:** Summary of infection and clinical factors associated with nasopharyngeal carcinoma

Potential risk factor	Summary of results
**Infection:**	
EBV serology	Higher titres associated risk factor [92,94-97]
	Lower anti-gp350 levels associated risk factor. Potential interaction with IgA anti-EBNA-1 [55]
	Stable, fluctuating or ascending IgA anti-VCA risk factor [90]
	IgA anti-VCA, anti-EA, anti-EBNA not associated [97]
	IgA/IgG anti-VCA, IgA/IgG anti-EA, anti-EBNA not associated [S10]
	Anti-gH/gL not associated [55]
Other infections	CMV, HSV, VZV no association [S11]
	SV40 – no association[S12]
	Core antigen of HBV associated with higher risk, other antigens/antibodies no association [S13]
	IM – potential association, depending upon how recent exposure was [S14]
	IM – no association [S3]
	Malaria – association not consistent[63]
**Other clinical:**	
Medical history	Allergic rhinitis associated risk factor [S15]
	Acute and/or chronic rhinosinusitis risk factor [S9,S16]
	Paranasal sinusitis risk factor [S17]
	Sinusitis potential risk factor [92]
	(Chronic) ear, nose (and throat) conditions risk factor [60,62,73] [S18]
	(Chronic) ear and nose diseases – inconclusive [83]
	Ear, nose and throat conditions – no association [64]
	Hayfever, tonsillectomy, heart disease, diabetes, cold sores, canker sores no association [73]
	Nasal polyps, childhood radiation treatment no association[S3]
	Result undocumented [S5]
Medication	Herbal medicines risk factor [S17,S19-S21]
	Herbal medicines – no association [S22]
	Home remedies in childhood risk factor [S7]
	Nasal balms, drops or oils – risk factor [58] [S17]
	Nasal balms, drops, ointments, oils or sprays – no association [59,64,83] [S3,S22]
	Nasal balms or oils – unclear [S21]
Oral hygiene	Frequent brushing, fewer filled/decayed teeth protective [61] [S23]
T-cells	Higher proportions of LMP-2 specific cytotoxic T-cells and CD4+/CD25+ T-cells risk factor [S24]
**Family history of NPC**	Risk factor [57,63,65,76,78] [S5,S18,S25-S31]
	Inconclusive evidence[60,92,95]
	No association [62,77]
	Results not clearly presented [64]
**Family history of cancer**	Risk factor [69,78,93] [S9,S27]
	No association [S29,S30]

CD – cluster of differentiation, CMV – cytomegalovirus, EA – early antigen, EBNA – Epstein Barr virus nuclear antigen, EBV – Epstein Barr virus, gp – glycoprotein, HBV – hepatitis B virus, HSV – herpes simplex virus, Ig – immunoglobulin, IM – infectious mononucleosis, LMP – latent membrane protein, NPC – nasopharyngeal carcinoma, SNP – single nucleotide polymorphism, SV40 – simian virus 40, VCA – viral capsid antigen, VZV – varicella zoster virus

*References marked with letter S are available in the **Online Supplementary Document**.

Overall, anti-EBV antibodies were associated with NPC. Among the seven papers, four documented this association with anti-VCA antibodies (three IgA [90,92,94-96], one IgG) [97], one anti-EA (IgA) [94], one anti-EA/EBNA-1 [95], two anti-EBV deoxyribonuclease [92,96], and one suggested that anti-gp350 was protective [55]. One showed no associations and two that some antibodies were not associated. Where stated, all used serum samples for testing.

In terms of medical history, the general trend of evidence was that a history of (chronic) ear nose and/or throat conditions was associated with NPC risk, as were herbal medicines. Nasal balms, drops, ointments, oils or sprays were generally not associated (five of seven papers).

Twenty publications analysed whether a family history of NPC was associated with personal risk, with the balance of evidence on the side of this being a risk factor (15 publications), in line with the genetic evidence presented in **Table 7**. Three other publications contained effect estimates that trended in the same direction. Among the 15, only one adjusted for genetic factors in the analysis; four others adjusted for other shared factors eg, diet. Five of the seven analyses examining whether family history of cancer as a whole was associated with personal NPC also had positive results; the likelihood and strength of the associations could be linked to the setting in which this work was undertaken, ie, how large a proportion of all cancer cases are due to NPC, although the studies were geographically confined within Asia.

**Table 7:** Summary of human genetic factors associated with nasopharyngeal carcinoma

Potential risk factor	References
**Angiogenesis: **	
VEGF	[S32,S33]
**Apoptosis**	
FAS/FAS-L	[S34-S36]
**Red blood cell antigens**	[61] [S37-S40]
**Cell cycle, growth and differentiation:**	
CCND1	[S41,S42]
EGF, EGFR	[S43]
MDM2	[S44-S46]
TP53	[S45,S47-S51]
**Cellular adhesion:**	
CD44	[S52]
CDH1	[S53]
MMP	[S54,S55]
**Cellular chaperones:**	
HSP-70	[S56]
**Cytokines and chemokines:**	
IFN-A	[S48]
IFN-G	[S57,S58]
IFN-GR1	[S59]
IL-1A/B	[S59-S61]
IL-1RN	[S59,S62]
IL-2	[S63]
IL-4RA	[S59]
IL-8	[S58,S64,S65]
IL-10	[S57,S59,S66-68]
IL-12	[S69,S70]
IL-16	[S71]
IL-18	[S66,S72,S73]
IL-27	[S70]
TGFB	[S74,S75]
TNFA	[S56,S76,S77]
**DNA damage and repair:**	
General	[S78,S79]
BPIFA1	[S80]
ERCC1	[S81]
ERCC2	[S82]
hOGG1	[S83]
NBS1	[S84]
XPC	[S85]
XRCC1	[S82,S83,S86,S87]
XRCC3	[S82,S88]
**HLA and associated genes:**	
HCGA9	[S59]
HLA region/type	[S38,S40,S89-S115]
HLA-A	[S59,S116-S118]
HLA-DQ	[S119,S120]
HLA-DR	[S120]
HLA-E	[S121-S123]
HLA-G	[S124]
MICA	[S125,S126]
TAP1	[S127,S128]
**Inflammation:**	
COX-2	[S129]
MAPKAPK2	[S130]
**Metabolism:**	
CYP1A1	[S1,S131]
CYP2A6	[S1]
CYP2E1	[89] [S1,S132-S137]
GSTM1	[S1,S131,S138-S147]
GSTP1	[S131,S133,S144]
GSTT1	[S1,S131,S138,S140-S142,S144,S147]
MPO	[S133]
MTHFR	[S148]
NAT2	[S131,S138]
NQO1	[S133]
**MicroRNAs:**	
MIR34	[S149]
Multiple	[S150]
**Other immune-related:**	
CR2	[S151-S153]
CTLA4	[S154]
DC SIGN	[S155]
IGK	[S156]
KIR	[S90,S96,S157]
PIGR	[S152,S158,S159]
TLR3	[S160]
TLR4	[S161]
TLR10	[S162]
**Other:**	
ACE	[S163]
BPIFA1 (PLUNC)	[S164]
CAV-1	[S165]
DLC-1	[S166]
IKB	[S167]
MAP2K4	[S168]
N4BP2	[S169]
NFKB	[S167,S170]
VDR	[S171]
**Genome wide-association study/other screening approaches**	[57,68,72] [S142,S172-S182]

*References marked with letter S are available in the **Online Supplementary Document**.

Human genetic risk factors for NPC have been thoroughly reviewed elsewhere, particularly by Hildesheim et al. [9]. **Table 7** thus briefly documents the publications on such factors, grouped by the genes of interest. Publications were generally of reasonable quality, with no risk of recall bias for the genetics component of the work. 137/158 (86.7%) had sample sizes that met our criteria for 90% power or more and 90/158 (57.0%) clearly documented recruiting individuals for both exposure arms from the same population. 60/158 (38.0%) were not assessed as having used appropriate statistical tests (usually due to the type of matching used).

Among the environmental and non-dietary risk factors papers, smoking and exposure to dust, smoke and fumes in both home and occupational environments, and socioeconomic status were common factors to analyse (**Table 8**). Both passive and personal smoking was analysed by a series of papers, with a wide variety of findings. Twelve publications found smoking to be a risk factor, an additional two reported a potential association and fifteen inconclusive or unclear associations. Nine publications found no evidence for an association.

**Table 8:** Summary of environmental and non-diet lifestyle factors associated with nasopharyngeal carcinoma

Potential risk factor	Summary of results
**Smoking/tobacco**	Risk factor (passive or personal) [56,57,60,61,65,69,72] [S2,S3,S5,S9,S21]
	Potential association [78] [S30]
	Passive or personal smoking no association [66-68,70,71,89] [S1,S18,S183]
	Results inconclusive or association unclear [58,59,62-64,73,76,83,92,93,95,96] [S17,S22,S26,S184]
**Cannabis and other drugs**	Cannabis – unclear association [67]
	Betel nuts – no association [58,72,89]
	Betel nuts – result undocumented [S5]
**Occupational exposures:**	
Wood dust and wood	Wood dust risk factor [60] [S184,S185]
	Wood dust – no clear associations [S186]
	Wood – no association [59,89]
	Wood cutting no association[71]
Smoke, fumes and dust	Inhalation of smoke and dust risk factor [59]
	Smoke, quarry, road or other dust risk factor [59]
	Fumes and smoke risk factors [73]
	Dust/exhaust risk factors [S187]
	Products of combustion risk factor if lengthy exposure [S18]
	Construction dust no association [59]
	Dust and/or fumes, smoke no association [64,73] [S18,S184]
	Wool and synthetic fibre dust no association [S188]
Formaldehyde and other chemicals	Chlorophenols risk factor [S189]
	Chemicals risk factors [73]
	Chemicals no association [S184]
	Chemical fumes inconclusive [64]
	Formaldehyde – no clear association [S186,S187]
	Formaldehyde no association [S184,S185]
	Bleaching agents, dyes and endotoxin no association[S188]
Solvents	Solvents no association [S185,S188]
	Solvents – impact inconclusive [76]
Heat	Potential risk factor [73]
	No association [S184]
Ventilation	Poor ventilation – results unclear [S21]
	No association [S22]
Overall occupation	Agricultural work potential risk factor [71]
	Occupational hazards risk factor [68]
	Occupation no association [58] [S17,S190]
	Occupation – result undocumented [S5]
	Occupational exposure to cotton dust, inks and potentially acids, bases and caustics risk factors [S188]
**Home environment:**	
Wood dust and wood	Wood fuel risk factor [76,85]
Smoke and fumes	Exposure to cooking fumes through having no separate kitchen or a range without a chimney risk factor [93]
	Poor ventilation risk factor [85] [S1,S17]
	Poor ventilation no association [58]
	Domestic fumes – no consistent association [67]
	Soot and cooking fuel no association [58]
Water	Reservoir sourced water protective [91]
**General dust and smoke**	General smoke exposure risk factor [S1]
	General dust exposure no association [S1]
**Family structure:**	
	Sibship size (particularly older siblings) risk factor; birth interval, maternal and paternal age no association [S191]
	Birth order – coming later is protective [S192]
	Marital status – no consistent association[63]
**Age:**	
	Age – rate increases with age [S193]
	Age – increases into 30-50 year age groups [S17]
	Age – more NPC than expected in individuals aged 30-39 [S104]
	Age – peak in late adolescence/early adulthood (15-24) and then later in life (65-79 years) [S195]
**Sex:**	
	Sex – more NPC in men [S17,S193,S194]
**Geographic region and ethnicity:**	
	Country – eastern and southeastern Asian are the highest rate regions; China, Malaysia and Singapore countries of note [26]
	Ethnicity – rates higher in individuals with Native American ancestry than White or Hispanic [S196]
	Ethnicity – in individuals with White and Black ancestry higher rates than Chinese [S193]
	Ethnicity – mainland Chinese vs Taiwanese, association with NPC [S21]
	Ethnicity – higher risk in mainlanders than in Taiwanese [S17]
	Immigration – immigrants have higher NPC rates, particularly if they originate from North Africa, Southeast Asia and Asian Arab countries [S197]
**Socio-demographic:**	
	Socioeconomic status – higher is a risk factor [58,85]
	Education – higher levels protective [60] [S3]
	Education – no consistent association [63]
	Education – no association [S190]

NPC – nasopharyngeal carcinoma

*References marked with letter S are available in the **Online Supplementary Document**.

Broad analyses of the impact of dust, fumes and smoke in occupational settings were inconclusive across included papers, however, within the different analyses of dust, wood dust was found to be associated in three analyses, but not in a fourth. Other occupational wood exposure was not found to be associated, but use of wood fuels in the home was found to be a risk factor within two publications. Poor ventilation in the workplace was not found to be associated with NPC risk. Within the home, the balance of evidence suggested that poor ventilation could be a risk factor. The association with socioeconomic status and education was inconsistent.

## DISCUSSION

BL, HL, GC and NPC are established as EBV-associated cancers. We present the first systematic review of epidemiological studies of risk factors in addition to EBV for the EBV-associated forms of these cancers, with an associated quality assessment. The 271 included publications provide a rich overview of our knowledge on the causes of EBV-associated BL, HL, GC and NPC. We highlight smoking, IM, and the *HLA* genetic region as risk factors for HL and being an older sibling as potentially protective. Rancid dairy products, salted fish, anti-EBV antibody and EBV DNA load, history of chronic ear, nose and/or throat conditions, herbal medicine use, family history, and genetics are NPC risk factors, as well as potentially smoking. In addition, fresh fruit and vegetables, slow cooked soup, and tea consumption are potentially protective against NPC. Anti-EBV antibody load was found to be associated with both BL and GC. We thus demonstrate a wider range of risk factors for NPC than the other EBV-associated cancers, which could either represent a more complex aetiology for NPC, or simply the limited number of publications for the other diseases.

In addition to the extensive array of studies and thus risk factors documented within this review, we note that our fundamental understanding of EBV and its associated cancers is underpinned by many important laboratory and epidemiological studies not captured by our specific and rigorous inclusion criteria [98]. Critically, we note the detailed evidence accumulated over decades linking endemic malaria to BL, which has been derived largely from settings where the EBV association with BL is robust [99]. Less strongly, HLA type, plants in the Euphorbiaceae family, and sociodemographic factors have been suggested to be associated with BL [5,100,101]. For GC, inference from studies where EBV status is unknown or negative is more problematic, as the EBV-associated form of the disease represents a distinct subtype with markedly different genomic, immunologic and pathological features [102], many of which have direct therapeutic relevance [103].

The major strength of this work is its systematic approach to examining published risk factors for EBV-associated cancers, including a quality assessment and mapping of the available literature. The global and temporal scope of our review allows a wide-reaching consolidation of the literature to date, as well as an identification of our knowledge gaps and targeting of future studies. As to its limitations, many of the NPC papers (particularly genetics studies) were only captured during the snowballing process, likely due to our decision to follow the STROBE guidelines for indexing when choosing our search terms. We excluded studies of genetic factors where samples were taken from cancer tissues, due to concerns about malignancy-induced genetic changes. This may, however, have excluded some genetics studies of loci that are unlikely to have been mutated in a cancerous cell. It should be noted that, where we document that a publication provides no evidence of an association, this does not mean that the converse is true; some studies were simply under-powered, thus confidence intervals were wide and estimates statistically uncertain. Our sample size calculations did not consider the implications of matching. We only documented analyses answering the declared aims or hypotheses of a publication; while *post hoc* exploratory data analyses can provide valuable insights, they require formal validation by subsequent studies and can sometimes arise due to data dredging [104].

Regarding the methodological quality of the included publications, we note several common features. Testing of multiple factors was common, increasing the likelihood of chance findings. Slightly over a third of publications used inappropriate statistical methods and only a quarter adjusted for our pragmatically minimal confounder set of age, ethnicity and sex. We acknowledge, however, that studies from certain settings would have recruited from ethnically homogenous populations, removing the need to control for ethnicity. Particular care should be taken interpreting the findings of dietary and environmental factor studies that do not adjust for upstream socioeconomic determinants of these factors and generally of recall bias. A few publications only presented results for factors positively associated with cancer.

As this review was specifically one of EBV-associated forms of our chosen cancers, we were limited by the number of studies that did not report EBV status. For GC, this reflects the relatively recent documentation of GC’s EBV-association and the fact that histological samples are not tested for the virus as standard. Without such testing, future meta-analyses will be substantially hampered. The close association between BL and EBV in endemic areas means that we could have included all publications from these regions regardless of EBV tumour status, however we opted for a consistent approach per tumour.

The major implications of our findings for public health are as follows. First, some of the critical risk and protective factors documented are modifiable eg, dietary elements, such as the consumption of rancid dairy products. These present opportunities for governmental interventions. Second, risk profiles can be built from these data for use as screening tools in areas of high cancer incidence. Third, our thorough documentation of the literature to date provides a signpost for future studies a) examining promising, but not fully proven, risk or protective factors in a broader span of geographical locations and b) to ensure that causal networks can be thoroughly mapped and thus confounders appropriately adjusted for [105]. Finally, it is importantly to reflect on the fact that the EBV-associated forms of certain cancers, eg, GC- differ from the non-EBV-associated forms. Thus reviews such as ours, where EBV-associated forms are considered separately, are critical to order to direct control efforts.

## CONCLUSIONS

We document 271 epidemiological publications on risk factors in addition to EBV for the EBV-associated forms of BL, GC, HL and NPC; the majority focussed on NPC. The quality of the available evidence was variable. The aetiology of EBV-associated cancers likely results from a complex intersection of genetic, clinical and dietary factors, which are difficult to pull apart through observational studies. A more strategic approach to building the evidence base should be undertaken with large, well-designed studies, in order to harmonise and clarify the evidence, particularly for GC and BL.

## Additional material

Online Supplementary Document
