# Supplementary material for: Risk factors for Epstein Barr virus-associated cancers: a systematic review, critical appraisal, and mapping of the epidemiological evidence
Source: J Glob Health. 2020 Mar 30;10(1):010405. doi: 10.7189/jogh.10.010405 (PMC7125417; doi:10.7189/jogh.10.010405)
Supplement: Online Supplementary Document [file jogh-10-010405-s001.pdf]

ONLINE SUPPLEMENTARY DOCUMENT

Appendix S1: Search strategies

Search strategies for MEDLINE (through Ovid) presented; strategies were adapted for EMBASE and the Web of Science. Lymphomas of interest: Hodgkin's and Burkitt's. Carcinomas of interest: gastric and nasopharyngeal.

Lymphoma search strategy (run 1st June 2017)

#	Terms	Hits
1	exp Herpesvirus 4, Human/	22,298
2	(EBV or Epstein-barr virus or Epstein barr Virus or HHV-4 or HHV4).mp. [mp=title, abstract, original title, name of substance word, subject heading word, keyword heading word, protocol supplementary concept word, rare disease supplementary concept word, unique identifier, synonyms]	35,532
3	1 OR 2	38,699
4	exp Risk Factors/	703,159
5	exp Epidemiology/	24,419
6	exp Cross-Sectional Studies/	247,653
7	exp Cohort Studies/	1,693,391
8	exp Case-Control Studies/	877,749
9	exp Clinical Trial/	806,724
10	(epidemiol* or risk factor or cross-sectional or cohort or case control or case-control or intervention study).mp. [mp=title, abstract, original title, name of substance word, subject heading word, keyword heading word, protocol supplementary concept word, rare disease supplementary concept word, unique identifier, synonyms]	1,507,190
11	4 OR 5 OR 6 OR 7 OR 8 OR 9 OR 10	3,642,502
12	exp Lymphoma/	162,050
13	exp Burkitt Lymphoma/	9,943
14	exp Hodgkin Disease/	33,391
15	exp Lymphoma, Non-Hodgkin/	94,494
16	(Burkitt* or Hodgkin* or lymphoepithelioma-like carcinoma* or lymphoma*).mp. [mp=title, abstract, original title, name of substance word, subject heading word, keyword heading word, protocol supplementary concept word, rare disease supplementary concept word, unique identifier, synonyms]	243,875
17	12 OR 13 OR 14 OR 15 OR 16	247,229
18	3 AND 11 AND 17	1,352

Carcinomas search strategy (run 4th August 2017)

#	Nasopharyngeal Terms	Hits
1	“Herpesvirus 4, Human” [MeSH] OR “EBV” OR “Epstein-Barr Virus” OR “Burkitt Herpesvirus” OR “Human Herpesvirus 4” OR “HHV4”	36,213
2	“Nasopharyngeal Neoplasms” [MeSH] OR “Cancer of Nasopharynx” OR “Nasopharyngeal cancer” OR “Cancer of the Nasopharynx” OR “Nasopharynx Neoplasms” OR “Nasopharyngeal Carcinoma” OR “NPC”	320,098
3	“Risk Factors” [MeSH] OR “Epidemiology” [MeSH] OR “Cross-Sectional Studies” [MeSH] OR “Cohort Studies” [MeSH] OR “Case-Control Studies” [MeSH] OR “Clinical Trial” [MeSH] OR “Epidemiol*” OR “risk factor” OR “cross-sectional” OR “cohort” OR “case control” OR “case-control” OR “intervention study” OR “association” OR “risk” OR “rate” OR “odds”	3,786,202
4	#1 AND #2 AND #4	735

#	Gastric Terms	Hits
1	“Herpesvirus 4, Human” [MeSH] OR “EBV” OR “Epstein-Barr Virus” OR “Burkitt Herpesvirus” OR “Human Herpesvirus 4” OR “HHV4”	36,213
2	“Stomach Neoplasms” [MeSH] OR “Cancer of the Stomach” OR “Gastric Cancer” OR “Gastric Neoplasms” OR “Neoplasms, Gastric” OR “Neoplasms, Stomach” OR “Stomach Cancer” OR “Gastric adenocarcinoma” OR “Gastric Carcinoma” OR “Adenocarcinoma of the Stomach”	96,514
3	“Risk Factors” [MeSH] OR “Epidemiology” [MeSH] OR “Cross-Sectional Studies” [MeSH] OR “Cohort Studies” [MeSH] OR “Case-Control Studies” [MeSH] OR “Clinical Trial” [MeSH] OR “Epidemiol*” OR “risk factor” OR “cross-sectional” OR “cohort” OR “case control” OR “case-control” OR “intervention study” OR “association” OR “risk” OR “rate” OR “odds”	3,786,202
4	#1 AND #2 AND #4	185

Appendix S2: Supplementary references

S1 Lourembam DS, Singh AR, Sharma TD, Singh TS, Singh TR, Singh LS. Evaluation of Risk Factors for Nasopharyngeal Carcinoma in a High-risk Area of India, the Northeastern Region. Asian Pacific journal of cancer prevention. APJCP. 2015;16:4927-35. Medline:26163617

S2 Nam JM, McLaughlin JK, Blot WJ. Cigarette smoking, alcohol, and nasopharyngeal carcinoma: a case-control study among U.S. whites. J Natl Cancer Inst. 1992;84:619-22. Medline:1556772 doi:10.1093/jnci/84.8.619

S3 Vaughan TL, Shapiro JA, Burt RD, Swanson GM, Berwick M, Lynch CF, et al. Nasopharyngeal cancer in a low-risk population: Defining risk factors by histological type. Cancer Epidemiol Biomarkers Prev. 1996;5:587-93. Medline:8824359

S4 Ruan HL, Xu FH, Liu WS, Feng QS, Chen LZ, Zeng YX, et al. Alcohol and tea consumption in relation to the risk of nasopharyngeal carcinoma in Guangdong, China. Front Med China. 2010;4:448-56. Medline:21110141 doi:10.1007/s11684-010-0280-6

S5 Hsu WL, Yu KJ, Chien YC, Chen TC, Lin CY, Tsou YA, et al. Familial tendency and environmental co-factors of nasopharyngeal carcinoma: The gene-environment interaction study on nasopharyngeal carcinoma in Taiwan. BMC Proceedings Conference: 7th Biannual International Symposium on Nasopharyngeal Carcinoma. 2015;10.

S6 Lo YL, Pan WH, Hsu WL, Chien YC, Chen JY, Hsu MM, et al. Partial least square discriminant analysis discovered a dietary pattern inversely associated with nasopharyngeal carcinoma risk. PLoS One. 2016;11:e0155892. Medline:27249558 doi:10.1371/journal.pone.0155892

S7 Jeannel D, Hubert A, de Vathaire F, Ellouz R, Camoun M, Ben Salem M, et al. Diet, living conditions and nasopharyngeal carcinoma in Tunisia–a case-control study. Int J Cancer. 1990;46:421-5. Medline:2394508 doi:10.1002/ijc.2910460316

S8 Zeng FF, Liu YT, Lin XL, Fan YY, Zhang XL, Xu CH, et al. Folate, vitamin B-6, vitamin B-12 and methionine intakes and risk for nasopharyngeal carcinoma in Chinese adults: a matched case-control study. Br J Nutr. 2016;115:121-8. Medline:26515433 doi:10.1017/S0007114515004146

S9 Nesic V, Sipetic S, Vlajinac H, Stosic-Divjak S, Jesic S. Risk Factors for the occurrence of undifferentiated carcinoma of nasopharyngeal type: A case-control study. Srp Arh Celok Lek. 2010;138:6-10. Medline:20422906 doi:10.2298/SARH1002006N

S10 Lanier AP, Henle W, Bender TR, Henle G, Talbot ML. Epstein-Barr virus-specific antibody titers in seven Alaskan natives before and after diagnosis of nasopharyngeal carcinoma. Int J Cancer. 1980;26:133-7. Medline:6259065 doi:10.1002/ijc.2910260203

S11 Bogger-Goren S, Gotlieb-Stematsky T, Rachima M, Barkowsky E, Schlomo-David J. Nasopharyngeal carcinoma in Israel: epidemiology and Epstein-Barr virus-related serology. Eur J Cancer Clin Oncol. 1987;23:1277-81. Medline:2824207 doi:10.1016/0277-5379(87)90108-8

S12 Mazzoni E, Martini F, Corallini A, Taronna A, Barbanti-Brodano G, Querzoli P, et al. Serologic investigation of undifferentiated nasopharyngeal carcinoma and simian virus 40 infection. Head Neck. 2016;38:232-6. Medline:25244358 doi:10.1002/hed.23879

S13 Ye YF, Xiang YQ, Fang F, Gao R, Zhang LF, Xie SH, et al. Hepatitis B virus infection and risk of nasopharyngeal carcinoma in southern China. Cancer Epidemiol Biomarkers Prev. 2015;24:1766-73. Medline:26364160 doi:10.1158/1055-9965.EPI-15-0344

S14 Levine R, Zhu K, Gu Y, Brann E, Hall I, Caplan L, et al. Self-reported infectious mononucleosis and 6 cancers: a population-based, case-control study. Scand J Infect Dis. 1998;30:211-4. Medline:9790125 doi:10.1080/00365549850160819

S15 Chung SD, Wu CS, Lin HC, Hung SH. Association Between Allergic Rhinitis and Nasopharyngeal Carcinoma: A Population-Based Study. Laryngoscope. 2014;124:1744-9. Medline:24272528 doi:10.1002/lary.24532

S16 Hung SH, Chen PY, Lin HC, Ting J, Chung SD. Association of Rhinosinusitis with nasopharyngeal carcinoma: A population-based study. Laryngoscope. 2014;124:1515-20. Medline:24114992 doi:10.1002/lary.24435
S17 Lin TM, Chen KP, Lin CC, Hsu MM, Tu SM, Chiang TC, et al. Retrospective study on nasopharyngeal carcinoma. J Natl Cancer Inst. 1973;51:1403-8. Medline:4762926 doi:10.1093/jnci/51.5.1403
S18 Yu MC, Garabrant DH, Huang TB, Henderson BE. Occupational and other non-dietary risk factors for nasopharyngeal carcinoma in Guangzhou, China. Int J Cancer. 1990;45:1033-9. Medline:2351484 doi:10.1002/ijc.2910450609
S19 Hildesheim A, West S, DeVeyra E, De Guzman MF, Jurado A, Jones C, et al. Herbal medicine use, Epstein-Barr virus, and risk of nasopharyngeal carcinoma. Cancer Res. 1992;52:3048-51. Medline:1317256
S20 Hildesheim A, West S, Jones C, Deveyra E, Hinuma Y. Epstein-Barr-Virus, Herbal medicine use, and risk of nasopharyngeal carcinoma. Am J Epidemiol. 1991;134:758.
S21 Lin TM, Chang HJ, Chen CJ. Risk factors for nasopharyngeal carcinoma. Anticancer Res. 1986;6:791-6. Medline:3752958
S22 Lin TM, Yang CS, Tu SM, Chen CJ, Kuo KC, Hirayama T. Interaction of factors associated with cancer of the nasopharynx. Cancer. 1979;44:1419-23. Medline:227565 doi:10.1002/1097-0142(197910)44:4<1419::AID-CNCR2820440437>3.0.CO;2-A
S23 Liu Z, Chang ET, Liu Q, Cai YL, Zhang Z, Chen GM, et al. Oral hygiene and risk of nasopharyngeal carcinoma-a population-based case-control study in China. Cancer Epidemiol Biomarkers Prev. 2016;25:1201-7. Medline:27197279 doi:10.1158/1055-9965.EPI-16-0149
S24 Mo WN, Tang AZ, Zhou L, Huang GW, Wang Z, Zeng Y. Analysis of Epstein-Barr viral DNA load, EBV-LMP2 specific cytotoxic T-lymphocytes and levels of CD4(+)CD25(+) T cells in patients with nasopharyngeal carcinomas positive for IgA antibody to EBV viral capsid antigen. Chin Med J (Engl). 2009;122:1173-8. Medline:19493466
S25 Friborg J, Wohlfahrt J, Koch A, Storm H, Olsen OR, Melbye M. Cancer susceptibility in nasopharyngeal carcinoma families - A population-based cohort study. Cancer Res. 2005;65:8567-72. Medline:16166338 doi:10.1158/0008-5472.CAN-04-4208
S26 Hsu WL, Yu KJ, Chien YC, Chiang CJ, Cheng YJ, Chen JY, et al. Familial tendency and risk of nasopharyngeal carcinoma in Taiwan: Effects of covariates on risk. Am J Epidemiol. 2011;173:292-9. Medline:21148719 doi:10.1093/aje/kwq358
S27 Laouamri S, Hamdi-Cherif M. Nasopharyngeal carcinoma and familial cancer in Setif (Algeria). Himmich H, editor. 2000. 88-323-0211-X.
S28 Liu Z, Fang F, Chang ET, Ye W. Cancer risk in the relatives of patients with nasopharyngeal carcinoma - A register-based cohort study in Sweden. Br J Cancer. 2015;112:1827-31. Medline:25928707 doi:10.1038/bjc.2015.140
S29 Ung A, Chen CJ, Levine PH, Cheng YJ, Brinton LA, Chen IH, et al. Familial and sporadic cases of nasopharyngeal carcinoma in Taiwan. Anticancer Res. 1999;19:661-5. Medline:10216473
S30 Xie SH, Yu ITS, Tse LA, Au JSK, Lau JSM. Tobacco smoking, family history, and the risk of nasopharyngeal carcinoma: a case-referent study in Hong Kong Chinese. Cancer Causes Control. 2015;26:913-21. Medline:25822573 doi:10.1007/s10552-015-0572-x
S31 Jia WH, Feng BJ, Xu ZL, Zhang XS, Huang P, Huang LX, et al. Familial risk and clustering of nasopharyngeal carcinoma in Guangdong, China. Cancer. 2004;101:363-9. Medline:15241835 doi:10.1002/cncr.20372
S32 Nasr HB, Chahed K, Bouaouina N, Chouchane L. Functional vascular endothelial growth factor -2578 C/A polymorphism in relation to nasopharyngeal carcinoma risk and tumor progression. Clin Chim Acta. 2008;395:124-9. Medline:18588866 doi:10.1016/j.cca.2008.05.022
S33 Wang T, Hu K, Ren JH, Zhu QY, Wu G, Peng G. Polymorphism of VEGF-2578C/A associated with the risk and aggressiveness of nasopharyngeal carcinoma in a

Chinese population. Mol Biol Rep. 2010;37:59-65. Medline:19340604 doi:10.1007/s11033-009-9526-2
S34 Ben Hadj Jrad B, Mahfouth W, Bouaouina N, Gabbouj S, Ben Ahmed S, Ltaief M, et al. A polymorphism in FAS gene promoter associated with increased risk of nasopharyngeal carcinoma and correlated with anti-nuclear autoantibodies induction. Cancer Lett. 2006;233:21-7. Medline:16473667 doi:10.1016/j.canlet.2005.02.037
S35 Cao Y, Miao XP, Huang MY, Deng L, Lin DX, Zeng YX, et al. Polymorphisms of death pathway genes FAS and FASL and risk of nasopharyngeal carcinoma. Mol Carcinog. 2010;49:944-50. Medline:20842669 doi:10.1002/mc.20676
S36 Zhu Q, Wang T, Ren J, Hu K, Liu W, Wu G. FAS-670A/G polymorphism: A biomarker for the metastasis of nasopharyngeal carcinoma in a Chinese population. Clin Chim Acta. 2010;411:179-83. Medline:19895798 doi:10.1016/j.cca.2009.10.024
S37 Hawkins BR, Simons MJ, Goh EH, Chia KB, Shanmugaratnam K. Immunogenetic aspects of nasopharyngeal carcinoma. II. Analysis of ABO, rhesus and MNSs red cell systems. Int J Cancer. 1974;13:116-21. Medline:4206461 doi:10.1002/ijc.2910130113
S38 Lanier A, Bender T, Talbot M, Wilmeth S, Tschopp C, Henle W, et al. Nasopharyngeal carcinoma in Alaskan Eskimos, Indians, and Aleuts: A review of cases and study of Epstein-Barr virus, HLA, and environmental risk factors. Cancer. 1980;46:2100-6. Medline:6253051 doi:10.1002/1097-0142(19801101)46:9<2100::AID-CNCR2820460932>3.0.CO;2-G
S39 Sheng L, Sun X, Zhang L, Su D. ABO blood group and nasopharyngeal carcinoma risk in a population of Southeast China. Int J Cancer. 2013;133:893-7. Medline:23389798 doi:10.1002/ijc.28087
S40 Simons MJ, Day NE, Wee GB, Shanmugaratnam K, Ho HC, Wong SH, et al. Nasopharyngeal carcinoma V: immunogenetic studies of Southeast Asian ethnic groups with high and low risk for the tumor. Cancer Res. 1974;34:1192-5. Medline:4842362
S41 Deng L, Zhao X-R, Pan KF, Wang Y, Deng X-Y, Lu Y-Y, et al. Cyclin D1 Polymorphism and the Susceptibility to NPC Using DHPLC. Sheng Wu Hua Xue Yu Sheng Wu Wu Li Xue Bao (Shanghai). 2002;34:16-20. Medline:11958128
S42 Shih LC, Tsai CW, Tsai MH, Tsou YA, Chang WS, Li FJ, et al. Association of cyclin D1 genotypes with nasopharyngeal carcinoma risk. Anticancer Res. 2012;32:1093-8. Medline:22399638
S43 Gao LB, Wei YS, Zhou B, Wang YY, Liang WB, Li C, et al. No association between epidermal growth factor and epidermal growth factor receptor polymorphisms and nasopharyngeal carcinoma. Cancer Genet Cytogenet. 2008;185:69-73. Medline:18722874 doi:10.1016/j.cancergencyto.2008.04.019
S44 Sousa H, Pando M, Breda E, Catarino R, Medeiros R. Role of the MDM2 SNP309 polymorphism in the initiation and early age of onset of nasopharyngeal carcinoma. Mol Carcinog. 2011;50:73-9. Medline:21229604 doi:10.1002/mc.20689
S45 Xiao M, Zhang L, Zhu X, Huang J, Jiang H, Hu S, et al. Genetic polymorphisms of MDM2 and TP53 genes are associated with risk of nasopharyngeal carcinoma in a Chinese population. BMC Cancer. 2010;10:147. Medline:20398418 doi:10.1186/1471-2407-10-147
S46 Zhou G, Zhai Y, Cui Y, Zhang X, Dong X, Yang H, et al. MDM2 promoter SNP309 is associated with risk of occurrence and advanced lymph node metastasis of nasopharyngeal carcinoma in Chinese population. Clin Cancer Res. 2007;13:2627-33. Medline:17473193 doi:10.1158/1078-0432.CCR-06-2281
S47 Birgander R, Sjalander A, Zhou Z, Fan C, Beckman L, Beckman G. p53 polymorphisms and haplotypes in nasopharyngeal cancer. Hum Hered. 1996;46:49-54. Medline:8825463 doi:10.1159/000154325
S48 Golovleva I, Birgander R, Sjalander A, Lundgren E, Beckman L. Interferon-alpha and p53 alleles involved in nasopharyngeal carcinoma. Carcinogenesis. 1997;18:645-7. Medline:9111194 doi:10.1093/carcin/18.4.645

S49 Hadhri-Guiga B, Toumi N, Khabir A, Sellami-Boudawara T, Ghorbel A, Daoud J, et al. Proline homozygosity in codon 72 of TP53 is a factor of susceptibility to nasopharyngeal carcinoma in Tunisia. Cancer Genet Cytogenet. 2007;178:89-93. Medline:17954263 doi:10.1016/j.cancergencyto.2007.05.013
S50 Sousa H, Santos AM, Catarinoa R, Pinto D, Vasconcelos A, Lopes C, et al. Linkage of TP53 codon 72 pro/pro genotype as predictive factor for nasopharyngeal carcinoma development. Eur J Cancer Prev. 2006;15:362-6. Medline:16835507 doi:10.1097/00008469-200608000-00010
S51 Tiwawech D, Srivatanakul P, Karaluk A, Ishida T. The p53 codon 72 polymorphism in Thai nasopharyngeal carcinoma. Cancer Lett. 2003;198:69-75. Medline:12893432 doi:10.1016/S0304-3835(03)00283-0
S52 Xiao M, Hu S, Zhang L, Huang J, Jiang H, Cai X. Polymorphisms of CD44 gene and nasopharyngeal carcinoma susceptibility in a Chinese population. Mutagenesis. 2013;28:577-82. Medline:23883608 doi:10.1093/mutage/get035
S53 Ben Nasr H, Hamrita B, Batbout M, Gabbouj S, Bouaouina N, Chouchane L, et al. A single nucleotide polymorphism in the E-cadherin gene promoter-160 C/A is associated with risk of nasopharyngeal cancer. Clin Chim Acta. 2010;411:1253-7. Medline:20462505 doi:10.1016/j.cca.2010.05.001
S54 Shao JY, Cao Y, Miao XP, Huang MY, Deng L, Hao JJ, et al. A single nucleotide polymorphism in the matrix metalloproteinase 2 promoter is closely associated with high risk of nasopharyngeal carcinoma in Cantonese from southern China. Chin J Cancer. 2011;30:620-6. Medline:21880183 doi:10.5732/cjc.010.10592
S55 Zhou G, Zhai Y, Cui Y, Qiu W, Yang H, Zhang X, et al. Functional polymorphisms and haplotypes in the promoter of the MMP2 gene are associated with risk of nasopharyngeal carcinoma. Hum Mutat. 2007;28:1091-7. Medline:17607721 doi:10.1002/humu.20570
S56 Jalbout M, Bouaouina N, Gargouri J, Corbex M, Ben Ahmed S, Chouchane L. Polymorphism of the stress protein HSP70-2 gene is associated with the susceptibility to the nasopharyngeal carcinoma. Cancer Lett. 2003;193:75-81. Medline:12691826 doi:10.1016/S0304-3835(02)00697-3
S57 Farhat K, Hassen E, Gabbouj S, Bouaouina N, Chouchane L. Interleukin-10 and interferon-gamma gene polymorphisms in patients with nasopharyngeal carcinoma. Int J Immunogenet. 2008;35:197-205. Medline:18312596 doi:10.1111/j.1744-313X.2008.00752.x
S58 Tai SH, Wei YS, Wang P, Zhou B, Ran P, Yang ZH, et al. Genetic polymorphisms of interferon-gamma and interleukin-8 in patients with nasopharyngeal carcinoma. [In Chinese] Sichuan Da Xue Xue Bao Yi Xue Ban. 2007;38:862-5. Medline:17953379
S59 Sousa H, Mesquita L, Ribeiro J, Catarino R, Breda E, Medeiros R. Polymorphisms in host immune response associated genes and risk of nasopharyngeal carcinoma development in Portugal. Immunobiology. 2016;221:145-52. Medline:26391153 doi:10.1016/j.imbio.2015.09.015
S60 Yang ZH, Dai Q, Zhong L, Zhang X, Guo QX, Li SN. Association of IL-1 polymorphisms and IL-1 serum levels with susceptibility to nasopharyngeal carcinoma. Mol Carcinog. 2011;50:208-14. Medline:21154765 doi:10.1002/mc.20706
S61 Zhu Y, Xu Y, Wei Y, Liang W, Liao M, Zhang L. Association of IL-1B gene polymorphisms with nasopharyngeal carcinoma in a Chinese population. Clin Oncol (R Coll Radiol). 2008;20:207-11. Medline:18289837 doi:10.1016/j.clon.2008.01.003
S62 Sousa H, Breda E, Santos AM, Catarino R, Pinto D, Canedo P, et al. IL-1RN VNTR polymorphism as a susceptibility marker for nasopharyngeal carcinoma in Portugal. Arch Oral Biol. 2013;58:1040-6. Medline:23562526 doi:10.1016/j.archoralbio.2013.02.004
S63 Wei YS, Lan Y, Zhang LA, Wang JC. Association of the Interleukin-2 Polymorphisms with Interleukin-2 Serum Levels and Risk of Nasopharyngeal Carcinoma. DNA Cell Biol. 2010;29:363-8. Medline:20438365 doi:10.1089/dna.2010.1019

S64 Ben Nasr H, Chahed K, Mestiri S, Bouaouina N, Snoussi K, Chouchane L. Association of IL-8 (-251)T/A polymorphism with susceptibility to and aggressiveness of nasopharyngeal carcinoma. Hum Immunol. 2007;68:761-9. Medline:17869651 doi:10.1016/j.humimm.2007.06.006
S65 Wei YS, Lan Y, Tang RG, Xu QQ, Huang Y, Nong HB, et al. Single nucleotide polymorphism and haplotype association of the interleukin-8 gene with nasopharyngeal carcinoma. Clin Immunol. 2007;125:309-17. Medline:17720627 doi:10.1016/j.clim.2007.07.010
S66 Pratesi C, Bortolin MT, Bidoli E, Tedeschi R, Vaccher E, Dolcetti R, et al. Interleukin-10 and interleukin-18 promoter polymorphisms in an Italian cohort of patients with undifferentiated carcinoma of nasopharyngeal type. Cancer Immunol Immunother. 2006;55:23-30. Medline:16059673 doi:10.1007/s00262-005-0688-z
S67 Tsai CW, Tsai MH, Shih LC, Chang WS, Lin CC, Bau DT. Association of Interleukin-10 (IL10) Promoter Genotypes with Nasopharyngeal Carcinoma Risk in Taiwan. Anticancer Res. 2013;33:3391-6. Medline:23898109
S68 Wei YS, Kuang XH, Zhu YH, Liang WB, Yang ZH, Tai SH, et al. Interleukin-10 gene promoter polymorphisms and the risk of nasopharyngeal carcinoma. Tissue Antigens. 2007;70:12-7. Medline:17559576 doi:10.1111/j.1399-0039.2007.00806.x
S69 Ben Chaaben A, Busson M, Douik H, Boukouaci W, Mamoghli T, Chaouch L, et al. Association of IL-12p40 +1188 A/C polymorphism with nasopharyngeal cancer risk and tumor extension. Tissue Antigens. 2011;78:148-51. Medline:21623733 doi:10.1111/j.1399-0039.2011.01702.x
S70 Wei YS, Lan Y, Luo B, Lu D, Nong HB. Association of variants in the interleukin-27 and interleukin-12 gene with nasopharyngeal carcinoma. Mol Carcinog. 2009;48:751-7. Medline:19148899 doi:10.1002/mc.20522
S71 Gao LB, Liang WB, Xue H, Rao L, Pan XM, Lv ML, et al. Genetic polymorphism of interleukin-16 and risk of nasopharyngeal carcinoma. Clin Chim Acta. 2009;409:132-5. Medline:19758567 doi:10.1016/j.cca.2009.09.017
S72 Farhat K, Hassen E, Bouzgarrou N, Gabbouj S, Bouaouina N, Chouchane L. Functional IL-18 promoter gene polymorphisms in Tunisian nasopharyngeal carcinoma patients. Cytokine. 2008;43:132-7. Medline:18555694 doi:10.1016/j.cyto.2008.05.004
S73 Nong LG, Luo B, Zhang L, Nong HB. Interleukin-18 gene promoter polymorphism and the risk of nasopharyngeal carcinoma in a Chinese population. DNA Cell Biol. 2009;28:507-13. Medline:19622039 doi:10.1089/dna.2009.0912
S74 Hu S, Zhou G, Zhang L, Jiang H, Xiao M. The effects of functional polymorphisms in the TGFbeta1 gene on nasopharyngeal carcinoma susceptibility. Otolaryngol Head Neck Surg. 2012;146:579-84. Medline:22282866 doi:10.1177/0194599811434890
S75 Wei YS, Zhu YH, Du B, Yang ZH, Liang WB, Lv ML, et al. Association of transforming growth factor-beta1 gene polymorphisms with genetic susceptibility to nasopharyngeal carcinoma. Clin Chim Acta. 2007;380:165-9. Medline:17368597 doi:10.1016/j.cca.2007.02.008
S76 Ho SY, Wang YJ, Huang PC, Tsai ST, Chen CH, Chen HHW, et al. Evaluation of the associations between the single nucleotide polymorphisms of the promoter region of the tumor necrosis factor-alpha gene and nasopharyngeal carcinoma. J Chin Med Assoc. 2006;69:351-7. Medline:16970270 doi:10.1016/S1726-4901(09)70272-2
S77 Sousa H, Breda E, Santos AM, Catarino R, Pinto D, Medeiros R. Genetic risk markers for nasopharyngeal carcinoma in Portugal: Tumor necrosis factor alpha-308G>A polymorphism. DNA Cell Biol. 2011;30:99-103. Medline:20874489 doi:10.1089/dna.2010.1086
S78 Cho EY, Hildesheim A, Chen CJ, Hsu MM, Chen IH, Mittl BF, et al. Nasopharyngeal carcinoma and genetic polymorphisms of DNA repair enzymes XRCC1 and hOGG1. Cancer Epidemiol Biomarkers Prev. 2003;12:1100-4. Medline:14578150
S79 Qin HD, Shugart YY, Bei JX, Pan QH, Chen LN, Feng QS, et al. Comprehensive pathway-based association study of DNA Repair gene variants and the risk of

nasopharyngeal carcinoma. Cancer Res. 2011;71:3000-8. Medline:21368091 doi:10.1158/0008-5472.CAN-10-0469
S80 Yew P-Y, Mushiroda T, Kiyotani K, Govindasamy GP, Yap L-F, Teo S-H, et al. Identification of a functional variant in SPLUNC1 associated with nasopharyngeal carcinoma susceptibility among Malaysian Chinese. Mol Carcinog. 2012;51:E74-82. Medline:22213098 doi:10.1002/mc.21857
S81 Yang ZH, Dai Q, Kong XL, Yang WL, Zhang L. Association of ERCC1 polymorphisms and susceptibility to nasopharyngeal carcinoma. Mol Carcinog. 2009;48:196-201. Medline:18615480 doi:10.1002/mc.20468
S82 Yang ZH, Du B, Wei YS, Zhang JH, Zhou B, Liang WB, et al. Genetic polymorphisms of the DNA repair gene and risk of nasopharyngeal carcinoma. DNA Cell Biol. 2007;26:491-6. Medline:17630853 doi:10.1089/dna.2006.0537
S83 Laantri N, Jalbout M, Khyatti M, Ayoub WB, Dahmoul S, Ayad M, et al. XRCC1 and hOGG1 genes and risk of nasopharyngeal carcinoma in North African countries. Mol Carcinog. 2011;50:732-7. Medline:21520294 doi:10.1002/mc.20754
S84 Zheng J, Zhang C, Jiang L, You YH, Liu YH, Lu JC, et al. Functional NBS1 Polymorphism Is Associated With Occurrence and Advanced Disease Status of Nasopharyngeal Carcinoma. Mol Carcinog. 2011;50:689-96. Medline:21656575 doi:10.1002/mc.20803
S85 Yang ZH, Liang WB, Jia J, Wei YS, Zhou B, Zhang L. The xeroderma pigmentosum group C gene polymorphisms and genetic susceptibility of nasopharyngeal carcinoma. Acta Oncol. 2008;47:379-84. Medline:17882560 doi:10.1080/02841860701558815
S86 Cao Y, Miao XP, Huang MY, Deng L, Hu LF, Ernberg I, et al. Polymorphisms of XRCC1 genes and risk of nasopharyngeal carcinoma in the Cantonese population. BMC Cancer. 2006;6:167. Medline:16796765 doi:10.1186/1471-2407-6-167
S87 Dai Q, Yang ZH, Ye YL. Association between genetic polymorphisms in the DNA repair gene X-ray repair cross-complementing 1-Arg399Gln and the risk of nasopharyngeal carcinoma in Chinese population from Sichuan province. [In Chinese]. Zhongguo Zu Zhi Gong Cheng Yan Jiu Yu Lin Chuang Kang Fu. 2007;11:5861-4.
S88 Liu JC, Tsai CW, Hsu CM, Chang WS, Li CY, Liu SP, et al. contribution of double strand break repair gene XRCC3 genotypes to nasopharyngeal carcinoma risk in Taiwan. Chin J Physiol. 2015;58:64-71. Medline:25687493 doi:10.4077/CJP.2015.BAD279
S89 Burt RD, Vaughan TL, McKnight B, Davis S, Beckmann AM, Smith AG, et al. Associations between human leukocyte antigen type and nasopharyngeal carcinoma in Caucasians in the United States. Cancer Epidemiol Biomarkers Prev. 1996;5:879-87. Medline:8922295
S90 Butsch Kovacic M, Martin M, Gao X, Fuksenko T, Chen CJ, Cheng YJ, et al. Variation of the killer cell immunoglobulin-like receptors and HLA-C genes in nasopharyngeal carcinoma. Cancer Epidemiol Biomarkers Prev. 2005;14:2673-7. Medline:16284396 doi:10.1158/1055-9965.EPI-05-0229
S91 Chan SH, Day NE, Kunaratnam N, Chia KB, Simons MJ. HLA and nasopharyngeal carcinoma in Chinese–a further study. Int J Cancer. 1983;32:171-6. Medline:6874140 doi:10.1002/ijc.2910320206
S92 Chan SH, Chew CT, Prasad U, Wee GB, Srinivasan N, Kunaratnam N. HLA and nasopharyngeal carcinoma in Malays. Br J Cancer. 1985;51:389-92. Medline:3855643 doi:10.1038/bjc.1985.52
S93 Chentoufi AA, AlZahrani KA, Mulia EA, AlMakoshi L, Alshareef R, Paz MR, et al. Association between the frequency of human leukocyte antigen class I and class II genes in Saudi population and relative risk of developing nasopharyngeal carcinoma. Hum Immunol. 2016;77:124. doi:10.1016/j.humimm.2016.07.183
S94 Cui HY. Apparent correlation between nasopharyngeal carcinoma and HLA phenotype. [In Chinese]. Zhonghua Zhong Liu Za Zhi. 1982;4:249-53. Medline:7166103
S95 Dardari R, Khyatti M, Jouhadi H, Benider A, Ettayebi H, Kahlain A, et al. Study of human leukocyte antigen class I phenotypes in Moroccan patients with

nasopharyngeal carcinoma. Int J Cancer. 2001;92:294-7. Medline:11291059 doi:10.1002/1097-0215(200102)9999:9999<::AID-IJC1177>3.0.CO;2-9
S96 Gao X, Tang M, Martin P, Zeng Y, Carrington M. Effect of HLA and KIR polymorphism on NPC risk. BMC Proceedings Conference: 7th Biannual International Symposium on Nasopharyngeal Carcinoma. 2015;10.
S97 Guo X, Winkler CA, Li J, Guan L, Tang M, Liao J, et al. Evaluation and integration of genetic signature for prediction risk of nasopharyngeal carcinoma in Southern China. BioMed Res Int. 2014;2014:434072. Medline:25180181 doi:10.1155/2014/434072
S98 Hall PJ, Levin AG, Entwistle CC, Knight SC, Wasunna A, Kung'u A, et al. HLA antigens in East African Black patients with Burkitt's lymphoma or nasopharyngeal carcinoma and in controls: a pilot study. Hum Immunol. 1982;5:91-105. Medline:7141899 doi:10.1016/0198-8859(82)90055-6
S99 Hildesheim A, Apple RJ, Chen CJ, Wang SS, Cheng YJ, Klitz W, et al. Association of HLA class I and II alleles and extended haplotypes with nasopharyngeal carcinoma in Taiwan. J Natl Cancer Inst. 2002;94:1780-9. Medline:12464650 doi:10.1093/jnci/94.23.1780
S100 Jing J, Louie E, Henderson BE, Terasaki P. Histocompatibility leukocyte antigen patterns in nasopharyngeal carcinoma cases from California. Natl Cancer Inst Monogr. 1977;47:153-6. Medline:613234
S101 Lakhanpal M, Singh LC, Rahman T, Sharma J, Singh MM, Kataki AC, et al. Contribution of susceptibility locus at HLA class I region and environmental factors to occurrence of nasopharyngeal cancer in Northeast India. Tumour Biol. 2015;36:3061-73. Medline:25514873 doi:10.1007/s13277-014-2942-5
S102 Li X, Ghandri N, Piancatelli D, Adams S, Chen D, Robbins FM, et al. Associations between HLA class I alleles and the prevalence of nasopharyngeal carcinoma (NPC) among Tunisians. J Transl Med. 2007;5:22. Medline:17480220 doi:10.1186/1479-5876-5-22
S103 Lu CC, Chen JC, Jin YT, Yang HB, Chan SH, Tsai ST. Genetic susceptibility to nasopharyngeal carcinoma within the HLA-A locus in Taiwanese. Int J Cancer. 2003;103:745-51. Medline:12516093 doi:10.1002/ijc.10861
S104 Mokni-Baizig N, Ayed K, Ayed FB, Ayed S, Sassi F, Ladgham A, et al. Association between HLA-A/-B antigens and -DRB1 alleles and nasopharyngeal carcinoma in Tunisia. Oncology. 2001;61:55-8. Medline:11474249 doi:10.1159/000055353
S105 Ooi EE, Ren EC, Chan SH. Association between microsatellites within the human MHC and nasopharyngeal carcinoma. Int J Cancer. 1997;74:229-32. Medline:9133461 doi:10.1002/(SICI)1097-0215(19970422)74:2<229::AID-IJC16>3.0.CO;2-8
S106 Ou B-X. H-Y. R, Fan Y, Liu L-M. Study on association between HLA-A, B, C, DR and nasopharyngeal cancer (NPC) in Guangzhou area. Chin J Cancer. 1985;4:5-7.
S107 Pasini E, Caggiari L, Dal Maso L, Martorelli D, Guidoboni M, Vaccher E, et al. Undifferentiated nasopharyngeal carcinoma from a nonendemic area: Protective role of HLA allele products presenting conserved EBV epitopes. Int J Cancer. 2009;125:1358-64. Medline:19536817 doi:10.1002/ijc.24515
S108 Simons MJ, Wee GB, Day NE, Morris PJ, Shanmugaratnam K, De-The GB. Immunogenetic aspects of nasopharyngeal carcinoma: I. Differences in HL-A antigen profiles between patients and control groups. Int J Cancer. 1974;13:122-34. Medline:4131857 doi:10.1002/ijc.2910130114
S109 Simons MJ, Wee GB, Chan SH, Shanmugaratnam K. Probable identification of an HL-A second-locus antigen associated with a high risk of nasopharyngeal carcinoma. Lancet. 1975;1:142-3. Medline:46054 doi:10.1016/S0140-6736(75)91433-6
S110 Tang M, Zeng Y, Poisson A, Marti D, Guan L, Zheng Y, et al. Haplotype-dependent HLA susceptibility to nasopharyngeal carcinoma in a Southern Chinese population. Genes Immun. 2010;11:334-42. Medline:20072141 doi:10.1038/gene.2009.109
S111 Tang M, Lautenberger JA, Gao X, Sezgin E, Hendrickson SL, Troyer JL, et al. The principal genetic determinants for nasopharyngeal carcinoma in China involve the HLA

Class I Antigen Recognition Groove. PLoS Genet. 2012;8:e1003103. Medline:23209447 doi:10.1371/journal.pgen.1003103
S112 Wu SB, Hwang SJ, Chang AS, Hsieh T, Hsu MM, Hsieh RP, et al. Human leukocyte antigen (HLA) frequency among patients with nasopharyngeal carcinoma in Taiwan. Anticancer Res. 1989;9:1649-53. Medline:2697183
S113 Yu KJ, Gao XJ, Chen CJ, Yang XH, Diehl SR, Goldstein A, et al. Association of human leukocyte antigens with nasopharyngeal carcinoma in high-risk multiplex families in Taiwan. Hum Immunol. 2009;70:910-4. Medline:19683024 doi:10.1016/j.humimm.2009.08.005
S114 Zhao M, Cai H, Zheng H, Yang X, Fang W, Zhang L, et al. Further evidence for the existence of major susceptibility of nasopharyngeal carcinoma in the region near HLA-A locus in Southern Chinese. Journal of Translational Medicine. 2012;10 (1) (no pagination).
S115 Zhu XN, Chen R, Kong FH, Liu W. Human leukocyte antigens -A, -B, -C, and -DR and nasopharyngeal carcinoma in northern China. Ann Otol Rhinol Laryngol. 1990;99:286-7. Medline:2327697 doi:10.1177/000348949009900407
S116 Burt RD, Vaughan TL, Nisperos B, Swanson M, Berwick M. A protective association between the HLA-A2 antigen and nasopharyngeal carcinoma in US Caucasians. Int J Cancer. 1994;56:465-7. Medline:8112882 doi:10.1002/ijc.2910560402
S117 Chin YM, Mushiroda T, Takahashi A, Kubo M, Krishnan G, Yap LF, et al. HLA-A SNPs and amino acid variants are associated with nasopharyngeal carcinoma in Malaysian Chinese. Int J Cancer. 2015;136:678-87. Medline:24947555
S118 Simons MJ, Wee GB, Chan SH, Shanmugaratnam K, Day NE, de-The G. Immunogenetic aspects of nasopharyngeal carcinoma (NPC) III. HL A type as a genetic marker of NPC predisposition to test the hypothesis that Epstein Barr virus is an etiological factor in NPC. Lyon, France: IARC (International Agency for Research on Cancer) Scientific Publications. 1975;No.11 II:249-58.
S119 Delfitri M. No Association between HLA-DQB1 Genotypes with Nasopharyngeal Carcinoma in Batak Ethnic Groups in Indonesia. Biomedical Research-India. 2011;22:235-40.
S120 Li PK, Poon AS, Tsao SY, Ho S, Tam JS, So AK, et al. No association between HLA-DQ and -DR genotypes with nasopharyngeal carcinoma in southern Chinese. Cancer Genet Cytogenet. 1995;81:42-5. Medline:7773959 doi:10.1016/0165-4608(94)00205-3
S121 Douik H, Romdhane NA, Guemira F. Are HLA-E*0103 alleles predictive markers for nasopharyngeal cancer risk? Pathol Res Pract. 2016;212:345-9. Medline:26896927 doi:10.1016/j.prp.2016.01.010
S122 Hassen E, Ghedira R, Ghandri N, Farhat K, Gabbouj S, Bouaouina N, et al. Lack of Association Between Human Leukocyte Antigen-E Alleles and Nasopharyngeal Carcinoma in Tunisians. DNA Cell Biol. 2011;30:603-9. Medline:21332388 doi:10.1089/dna.2010.1140
S123 Hirankarn N, Kimkong I, Mutirangura A. HLA-E polymorphism in patients with nasopharyngeal carcinoma. Tissue Antigens. 2004;64:588-92. Medline:15496202 doi:10.1111/j.1399-0039.2004.00311.x
S124 Ghandri N, Gabbouj S, Farhat K, Bouaouina N, Abdelaziz H, Nouri A, et al. Association of HLA-G polymorphisms with nasopharyngeal carcinoma risk and clinical outcome. Hum Immunol. 2011;72:150-8. Medline:20969909 doi:10.1016/j.humimm.2010.10.006
S125 Douik H, Ben Chaaben A, Attia Romdhane N, Romdhane HB, Mamoghli T, Fortier C, et al. Association of MICA-129 polymorphism with nasopharyngeal cancer risk in a Tunisian population. Hum Immunol. 2009;70:45-8. Medline:19000729 doi:10.1016/j.humimm.2008.10.008
S126 Tian W, Zeng XM, Li LX, Jin HK, Luo QZ, Wang F, et al. Gender-specific associations between MICA-STR and nasopharyngeal carcinoma in a southern Chinese Han population. Immunogenetics. 2006;58:113-21. Medline:16547745 doi:10.1007/s00251-006-0093-6

S127 Hassen E, Farhat K, Gabbouj S, Jalbout M, Bouaouina N, Chouchane L. TAP1 gene polymorphisms and nasopharyngeal carcinoma risk in a Tunisian population. Cancer Genet Cytogenet. 2007;175:41-6. Medline:17498556 doi:10.1016/j.cancergencyto.2007.01.009
S128 Sui J, Mo Q, Li XJ, Ma J, Ren YX, Gao W, et al. [Transporter associated with antigen processing 1 637A/G gene polymorphisms and susceptibility to nasopharyngeal carcinoma in Han population in Yunnan Province, China]. [Chinese]. Zhonghua er bi yan hou tou jing wai ke za zhi =. Zhonghua Er Bi Yan Hou Tou Jing Wai Ke Za Zhi. 2010;45:238-43. Medline:20450707
S129 Ben Nasr H, Chahed K, Bouaouina N, Chouchane L. PTGS2 (COX-2) -765 G > C functional promoter polymorphism and its association with risk and lymph node metastasis in nasopharyngeal carcinoma. Mol Biol Rep. 2009;36:193-200. Medline:17968676 doi:10.1007/s11033-007-9166-3
S130 Yang L, Liu B, Qiu F, Huang B, Li Y, Huang D, et al. The effect of functional MAPKAPK2 copy number variation CNV-30450 on elevating nasopharyngeal carcinoma risk is modulated by EBV infection. Carcinogenesis. 2014;35:46-52. Medline:24056810 doi:10.1093/carcin/bgt314
S131 Cheng YJ, Chien YC, Hildesheim A, Hsu MM, Chen IH, Chuang J, et al. No association between genetic polymorphisms of CYP1A1, GSTM1, GSTT1, GSTP1, NAT2, and nasopharyngeal carcinoma in Taiwan. Cancer Epidemiol Biomarkers Prev. 2003;12:179-80. Medline:12582034
S132 Ben Chaaben A, Abaza H, Douik H, Chaouch L, Ayari F, Ouni N, et al. Genetic polymorphism of cytochrome P450 2E1 and the risk of nasopharyngeal carcinoma. Bull Cancer. 2015;102:967-72. Medline:26582733 doi:10.1016/j.bulcan.2015.09.013
S133 Guo X, Zeng Y, Deng H, Liao J, Zheng Y, Li J, et al. Genetic Polymorphisms of CYP2E1, GSTP1, NQO1 and MPO and the Risk of Nasopharyngeal Carcinoma in a Han Chinese Population of Southern China. BMC Res Notes. 2010;3:212. Medline:20663217 doi:10.1186/1756-0500-3-212
S134 Hildesheim A, Chen CJ, Caporaso NE, Cheng YJ, Hoover RN, Hsu MM, et al. Cytochrome P4502E1 genetic polymorphisms and risk of nasopharyngeal carcinoma: results from a case-control study conducted in Taiwan. Cancer Epidemiol Biomarkers Prev. 1995;4:607-10. Medline:8547826
S135 Hildesheim A, Anderson LM, Chen CJ, Cheng YJ, Brinton LA, Daly AK, et al. CYP2E1 genetic polymorphisms and risk of nasopharyngeal carcinoma in Taiwan. J Natl Cancer Inst. 1997;89:1207-12. Medline:9274915 doi:10.1093/jnci/89.16.1207
S136 Jia WH, Pan QH, Qin HD, Xu YF, Shen GP, Chen L, et al. A Case-control and a family-based association study revealing an association between CYP2E1 polymorphisms and nasopharyngeal carcinoma risk in Cantonese. Carcinogenesis. 2009;30:2031-6. Medline:19805575 doi:10.1093/carcin/bgp239
S137 Kongruttanachok N, Sukdikul S, Setavarin S, Kerekhjanarong V, Supiyaphun P, Voravud N, et al. Cytochrome P450 2E1 polymorphism and nasopharyngeal carcinoma development in Thailand: a correlative study. BMC Cancer. 2001;1:4. Medline:11389775 doi:10.1186/1471-2407-1-4
S138 Bendjemana K, Abdennebi M, Gara S, Jmal A, Ghanem A, Touati S, et al. Genetic polymorphism of gluthation-S transferases and N-acetyl transferases 2 and nasopharyngeal carcinoma: the Tunisia experience. Bull Cancer. 2006;93:297-302. Medline:16567317
S139 Da S-J, Liang B, Wu H-L, Chen M-N, Liu P-X. Relationship between GSTM1 gene polymorphism and genetic susceptibility in nasopharyngeal carcinoma. Shi yong ai zheng za zhi= The practical journal of cancer. 2002;17:617-8.
S140 Deng ZL, Wei YP, Ma Y. Frequent genetic deletion of detoxifying enzyme GSTM1 and GSTT1 genes in nasopharyngeal carcinoma patients in Guangxi Province, China]. [In Chinese] Zhonghua Zhong Liu Za Zhi. 2004;26:598-600. Medline:15634519

S141 Deng Z, Wei Y, Luo W, Liao Z, Ma Y. Glutathione S-transferase M1 and T1 Gene Deletion Associated with Increased Susceptibility to Nasopharyngeal Carcinoma. Chinese-German J Clin Oncol. 2005;4:276-8. doi:10.1007/s10330-005-0363-z
S142 Guo X, O'Brien SJ, Zeng Y, Nelson GW, Winkler CA. GSTM1 and GSTT1 gene deletions and the risk for nasopharyngeal carcinoma in Han Chinese. Cancer Epidemiol Biomarkers Prev. 2008;17:1760-3. Medline:18628429 doi:10.1158/1055-9965.EPI-08-0149
S143 He Y, Zhou GQ, Li X, Dong XJ, Chai XQ, Yao KT. Correlation of polymorphism of the coding region of glutathione S- transferase M1 to susceptibility of nasopharyngeal carcinoma in South China population. Ai Zheng. 2009;28:5-7. Medline:19448408
S144 Jiang Y, Li N, Dong P, Zhang N, Sun Y, Han M, et al. Polymorphisms in GSTM1, GSTTI and GSTP1 and nasopharyngeal cancer in the east of China: A case-control study. Asian Pac J Cancer Prev. 2011;12:3097-100. Medline:22393996
S145 Nazar-Stewart V, Vaughan TL, Burt RD, Chen C, Berwick M, Swanson GM. Glutathione S-transferase M1 and susceptibility to nasopharyngeal carcinoma. Cancer Epidemiol Biomarkers Prev. 1999;8:547-51. Medline:10385146
S146 Tiwawech D, Srivatanakul P, Karalak A, Ishida T. Glutathione S-transferase M1 gene polymorphism in Thai nasopharyngeal carcinoma. Asian Pac J Cancer Prev. 2005;6:270-5. Medline:16235985
S147 Wei Y, Long X, Liu Z, Ma Y, Deng Z. Genetic polymorphism of glutathione-S-transferase M1 and T1 in associated with carcinogenesis of hepatocellular carcinoma and nasopharyngeal carcinoma. Chinese-German J Clin Oncol. 2012;11:138-41. doi:10.1007/s10330-011-0945-x
S148 Cao Y, Miao XP, Huang MY, Deng L, Liang XM, Lin DX, et al. Polymorphisms of methylenetetrahydrofolate reductase are associated with a high risk of nasopharyngeal carcinoma in a smoking population from Southern China. Mol Carcinog. 2010;49:928-34. Medline:20721969 doi:10.1002/mc.20669
S149 Li L, Wu J, Sima XT, Bai P, Deng W, Deng XK, et al. Interactions of miR-34b/c and TP-53 polymorphisms on the risk of nasopharyngeal carcinoma. Tumour Biol. 2013;34:1919-23. Medline:23504554 doi:10.1007/s13277-013-0736-9
S150 Qiu F, Yang L, Zhang L, Yang X, Yang R, Fang W, et al. Polymorphism in mature microRNA-608 sequence is associated with an increased risk of nasopharyngeal carcinoma. Gene. 2015;565:180-6. Medline:25861865 doi:10.1016/j.gene.2015.04.008
S151 Fan Q, He JF, Wang QR, Cai HB, Sun XG, Zhou XX, et al. Functional polymorphism in the 5′-UTR of CR2 is associated with susceptibility to nasopharyngeal carcinoma. Oncol Rep. 2013;30:11-6. Medline:23612877 doi:10.3892/or.2013.2421
S152 Hirunsatit R, Kongruttanachok N, Shotelersuk K, Supiyaphun P, Voravud N, Sakuntabhai A, et al. Polymeric immunoglobulin receptor polymorphisms and risk of nasopharyngeal cancer. BMC Genet. 2003;4:3. Medline:12546713 doi:10.1186/1471-2156-4-3
S153 Sousa H, Bastos M, Ribeiro J, Oliveira S, Breda E, Catarino R, et al. 5′UTR +24T>C CR2 is not associated with nasopharyngeal carcinoma development in the North Region of Portugal. Oral Dis. 2016;22:280-4. Medline:26748973 doi:10.1111/odi.12436
S154 Xiao M, Qi F, Chen X, Luo Z, Zhang L, Zheng C, et al. Functional polymorphism of cytotoxic T-lymphocyte antigen 4 and nasopharyngeal carcinoma susceptibility in a Chinese population. Int J Immunogenet. 2010;37:27-32. Medline:19922464 doi:10.1111/j.1744-313X.2009.00888.x
S155 Xu YF, Liu WL, Dong JQ, Liu WS, Feng QS, Chen LZ, et al. Sequencing of DC-SIGN promoter indicates an association between promoter variation and risk of nasopharyngeal carcinoma in cantonese. BMC Med Genet. 2010;11:161. Medline:21067616 doi:10.1186/1471-2350-11-161
S156 Ren W, Zheng H, Li M, Deng L, Li XL, Pan KF, et al. A functional single nucleotide polymorphism site detected in nasopharyngeal carcinoma-associated transforming gene Tx. Cancer Genet Cytogenet. 2005;157:49-52. Medline:15676147 doi:10.1016/j.cancergencyto.2004.06.009

S157 Gao SQ, Deng ZH. A study on the correlation of killer immunoglobulin-like receptor gene diversity with nasopharyngeal carcinoma in the southern chinese han population. Vox Sang. 2012;103:265-6.
S158 Fan Q, Jia WH, Zhang RH, Yu XJ, Chen LZ, Feng QS, et al. Correlation of polymeric immunoglobulin receptor gene polymorphisms to susceptibility of nasopharyngeal carcinoma.. [In Chinese]. Ai Zheng. 2005;24:915-8. Medline:16086865
S159 Fan Q, Jia WH, Zhang RH, Yu XJ, Chen LZ, Feng QS, et al. Association of pIgR polymorphisms with nasopharyngeal carcinoma. Chin J Cancer Res. 2006;18:168-72. doi:10.1007/s11670-006-0168-9
S160 He JF, Jia WH, Fan Q, Zhou XX, Qin HD, Shugart YY, et al. Genetic polymorphisms of TLR3 are associated with Nasopharyngeal carcinoma risk in Cantonese population. BMC Cancer. 2007;7:194. Medline:17939877 doi:10.1186/1471-2407-7-194
S161 Song C, Chen LZ, Zhang RH, Yu XJ, Zeng YX. Functional variant in the 3′-untranslated region of toll-like receptor 4 is associated with nasopharyngeal carcinoma risk. Cancer Biol Ther. 2006;5:1285-91. Medline:16969132 doi:10.4161/cbt.5.10.3304
S162 Zhou XX, Jia WH, Shen GP, Qin HD, Yu XJ, Chen LZ, et al. Sequence variants in toll-like receptor 10 are associated with nasopharyngeal carcinoma risk. Cancer Epidemiol Biomarkers Prev. 2006;15:862-6. Medline:16702361 doi:10.1158/1055-9965.EPI-05-0874
S163 Li ZH, Pan XM, Han BW, Han HB, Zhang Z, Gao LB. No association between ACE polymorphism and risk of nasopharyngeal carcinoma. J Renin Angiotensin Aldosterone Syst. 2012;13:210-5. Medline:22064901 doi:10.1177/1470320311426168
S164 He Y, Zhou G, Zhai Y, Dong X, Lv L, He F, et al. Association of PLUNC gene polymorphisms with susceptibility to nasopharyngeal carcinoma in a Chinese population. J Med Genet. 2005;42:172-6. Medline:15689457 doi:10.1136/jmg.2004.022616
S165 Tsou YA, Tsai CW, Tsai MH, Chang WS, Li FJ, Liu YF, et al. Association of Caveolin-1 genotypes with nasopharyngeal carcinoma susceptibility in Taiwan. Anticancer Res. 2011;31:3629-32. Medline:21965789
S166 Feng XL, Zhou W, Li H, Fang WY, Zhou YB, Yao KT, et al. The DLC-1 -29A/T polymorphism is not associated with nasopharyngeal carcinoma risk in Chinese population. Genet Test. 2008;12:345-9. Medline:18627284 doi:10.1089/gte.2007.0121
S167 Liu Y, Qiu FM, Yang L, Yang RR, Yang XR, Huang DS, et al. Polymorphisms of NF kappa B1 and I kappa B alpha and their synergistic effect on nasopharyngeal carcinoma susceptibility. BioMed Res Int. 2015;2015:362542. Medline:26161396
S168 Zheng J, Liu B, Zhang LY, Jiang L, Huang BF, You YH, et al. The protective role of polymorphism MKK4-1304 T > G in nasopharyngeal carcinoma is modulated by Epstein-Barr virus' infection status. Int J Cancer. 2012;130:1981-90. Medline:21702039 doi:10.1002/ijc.26253
S169 Zheng MZ, Qin HD, Yu XJ, Zhang RH, Chen LZ, Feng QS, et al. Haplotype of gene Nedd4 binding protein 2 associated with sporadic nasopharyngeal carcinoma in the Southern Chinese population. J Transl Med. 2007;5:36. Medline:17626640 doi:10.1186/1479-5876-5-36
S170 Zhou B, Rao L, Li Y, Gao LB, Wang YY, Chen Y, et al. A functional insertion/deletion polymorphism in the promoter region of NFKB1 gene increases susceptibility for nasopharyngeal carcinoma. Cancer Lett. 2009;275:72-6. Medline:19006646 doi:10.1016/j.canlet.2008.10.002
S171 Huang X, Cao Z, Zhang Z, Yang Y, Wang J, Fang D. No association between Vitamin D receptor gene polymorphisms and nasopharyngeal carcinoma in a Chinese Han population. Biosci Trends. 2011;5:99-103. Medline:21788694 doi:10.5582/bst.2011.v5.3.99
S172 Bei JX, Li Y, Jia WH, Feng BJ, Zhou GQ, Chen LZ, et al. A genome-wide association study of nasopharyngeal carcinoma identifies three new susceptibility loci. Nat Genet. 2010;42:599-603. Medline:20512145 doi:10.1038/ng.601

S173 Cui Q, Xu M, Bei JX, Zeng YX. Risk prediction of nasopharyngeal carcinoma by detecting host genetic and Epstein-Barr virus variation in saliva. BMC Proceedings Conference: 7th Biannual International Symposium on Nasopharyngeal Carcinoma. 2015;10.

S174 Dai W, Zheng H, Cheung AKL, Tang CSM, Ko JMY, Wong BWY, et al. Whole-exome sequencing identifies MST1R as a genetic susceptibility gene in nasopharyngeal carcinoma. Proc Natl Acad Sci U S A. 2016;113:3317-22. Medline:26951679 doi:10.1073/pnas.1523436113

S175 Feng BJ, Huang W, Shugart YY, Lee MK, Zhang F, Xia JC, et al. Genome-wide scan for familial nasopharyngeal carcinoma reveals evidence of linkage to chromosome 4. Nat Genet. 2002;31:395-9. Medline:12118254 doi:10.1038/ng932

S176 Yee Ko JM, Dai W, Wong EHW, Kwong D, Ng WT, Lee A, et al. Multigene pathway-based analyses identify nasopharyngeal carcinoma risk associations for cumulative adverse effects of TERT-CLPTM1L and DNA double-strand breaks repair. Int J Cancer. 2014;135:1634-45. Medline:24615621 doi:10.1002/ijc.28802

S177 Moumad K, Lascorz J, Bevier M, Khyatti M, Ennaji MM, Benider A, et al. Genetic polymorphisms in host innate immune sensor genes and the risk of nasopharyngeal carcinoma in North Africa. G3: Genes, Genomes. G3 (Bethesda). 2013;3:971-7. Medline:23576520 doi:10.1534/g3.112.005371

S178 Ng CC, Yew PY, Puah SM, Krishnan G, Yap LF, Teo SH, et al. A genome-wide association study identifies ITGA9 conferring risk of nasopharyngeal carcinoma. J Hum Genet. 2009;54:392-7. Medline:19478819 doi:10.1038/jhg.2009.49

S179 Su WH, Tse KP, Yang ML, Shugart YY, Chang YS. Copy number variation analysis identified multiple genetic variants related to nasopharyngeal carcinoma predisposition. Cancer Research Conference: 102nd Annual Meeting of the American Association for Cancer Research, AACR. 2011;71.

S180 Tse KP, Su WH, Chang KP, Tsang NM, Yu CJ, Tang P, et al. Genome-wide Association Study Reveals Multiple Nasopharyngeal Carcinoma-Associated Loci within the HLA Region at Chromosome 6p21.3. Am J Hum Genet. 2009;85:194-203. Medline:19664746 doi:10.1016/j.ajhg.2009.07.007

S181 Xiong W, Zeng ZY, Xia JH, Xia K, Shen SR, Li XL, et al. A susceptibility locus at chromosome 3p21 linked to familial nasopharyngeal carcinoma. Cancer Res. 2004;64:1972-4. Medline:15026332 doi:10.1158/0008-5472.CAN-03-3253

S182 Zheng H, Dai W, Tang C, Cheung A, Ko J, Sham P, et al. Unraveling the genetic basis of nasopharyngeal carcinoma using next-generation sequencing approaches. Cancer Research Conference: 106th Annual Meeting of the American Association for Cancer Research, AACR. 2015;75.

S183 Mabuchi K, Bross DS, Kessler II. Cigarette smoking and nasopharyngeal carcinoma. Cancer. 1985;55:2874-6. Medline:3995493 doi:10.1002/1097-0142(19850615)55:12<2874::AID-CNCR2820551228>3.0.CO;2-4

S184 Zhu R, Levine RS, Brann EA, Gnepp DR, Baum MK. A population-based case-control study of the relationship between cigarette-smoking and nasopharyngeal cancer (United-States). Cancer Causes Control. 1995;6:507-12. Medline:8580298 doi:10.1007/BF00054158

S185 Hildesheim A, Dosemeci M, Chan CC, Chen CJ, Cheng YJ, Hsu MM, et al. Occupational exposure to wood, formaldehyde, and solvents and risk of nasopharyngeal carcinoma. Cancer Epidemiol Biomarkers Prev. 2001;10:1145-53. Medline:11700262

S186 Vaughan TL, Stewart PA, Teschke K, Lynch CF, Swanson GM, Lyon JL, et al. Occupational exposure to formaldehyde and wood dust and nasopharyngeal carcinoma. Occup Environ Med. 2000;57:376-84. Medline:10810126 doi:10.1136/oem.57.6.376

S187 West S, Hildesheim A, Dosemeci M. Non-viral risk factors for nasopharyngeal carcinoma in the Philippines: results from a case-control study. Int J Cancer. 1993;55:722-7. Medline:7503957 doi:10.1002/ijc.2910550504

S188 Li W, Ray RM, Gao DL, Fitzgibbons ED, Seixas NS, Camp JE, et al. Occupational risk factors for nasopharyngeal cancer among female textile workers in Shanghai, China. Occup Environ Med. 2006;63:39-44. Medline:16361404 doi:10.1136/oem.2005.021709
S189 Mirabelli MC, Hoppin JA, Tolbert PE, Herrick RF, Gnepp DR, Brann EA. Occupational exposure to chlorophenol and the risk of nasal and nasopharyngeal cancers among U.S. men aged 30 to 60. Am J Ind Med. 2000;37:532-41. Medline:10723047 doi:10.1002/(SICI)1097-0274(200005)37:5<532::AID-AJIM9>3.0.CO;2-A
S190 Shanmugaratnam K, Tye CY, Goh EH, Chia KB. Etiological factors in nasopharyngeal carcinoma: a hospital-based, retrospective, case-control, questionnaire study. In: de-Thé G, Ito Y, editors. Nasopharyngeal Carcinoma: Etiology and Control: International Agency for Research on Cancer; 1978. p. 199-212.
S191 Liu Z, Fang F, Chang ET, Adami HO, Ye WM. Sibship size, birth order and risk of nasopharyngeal carcinoma and infectious mononucleosis: a nationwide study in Sweden. Int J Epidemiol. 2016;45:825-34. Medline:25925268 doi:10.1093/ije/dyv038
S192 Liu Z, Coghill AE, Pfeiffer RM, Hsu WL, Lou PJ, Wang CP, et al. Birth order and risk of nasopharyngeal carcinoma in multiplex families from Taiwan. Int J Cancer. 2016;139:2467-73. Medline:27537611 doi:10.1002/ijc.30390
S193 Burt RD, Vaughan TL, McKnight B. Descriptive epidemiology and survival analysis of nasopharyngeal carcinoma in the United States. Int J Cancer. 1992;52:549-56. Medline:1399135 doi:10.1002/ijc.2910520409
S194 Zong YS, Sham JST, Ng MH, Ou XT, Guo YQ, Zheng SA, et al. Immunoglobulin-a against viral capsid antigen of Epstein-Barr-Virus and indirect mirror examination of the nasopharynx in the detection of asymptomatic nasopharyngeal carcinoma. Cancer. 1992;69:3-7. Medline:1309307 doi:10.1002/1097-0142(19920101)69:1<3::AID-CNCR2820690104>3.0.CO;2-7
S195 Bray F, Haugen M, Moger TA, Tretli S, Aalen OO, Grotmol T. Age-incidence curves of nasopharyngeal carcinoma worldwide: Bimodality in low-risk populations and aetiologic implications. Cancer Epidemiol Biomarkers Prev. 2008;17:2356-65. Medline:18768504 doi:10.1158/1055-9965.EPI-08-0461
S196 Broehm C, Meisner A, Barry M, Wiggins C, Bocklage T. EBV-related nasopharyngeal carcinoma in Native Americans of New Mexico. Lab Invest. 2017;97:322A.
S197 Mousavi SM, Sundquist J, Hemminki K. Nasopharyngeal and hypopharyngeal carcinoma risk among immigrants in Sweden. Int J Cancer. 2010;127:2888-92. Medline:21351268 doi:10.1002/ijc.25287

Table S1: Included publications

BL- Burkitt's lymphoma, GC- gastric carcinoma, HL- Hodgkin's lymphoma, NPC- nasopharyngeal carcinoma, *Some publications stated the time frame of recruitment for cases, not controls; ^12 countries- Australia, Canada, Denmark, Estonia, India, Japan, Singapore, Switzerland, Thailand, The Philippines, UK, US; † or underlying population for cohort studies

Author	Cancer	Year of publication	Country	Year of study*	Study design	Control group†	Main risk factor focus
de-Thé *et al.*[1]	BL	1978	Uganda	1972-1977	Case-control	Population (children)	Infection and clinical
Geser *et al.*[2]	BL	1982	Uganda	1972-1977	Case-control	Population (children)	Infection and clinical
Alexander *et al.*[3]	HL	2000	UK	1991-1995	Case-control	Primary care	Infection and clinical
Alexander *et al.*[4]	HL	2003	UK	1993-1997	Case-control	Primary care	Infection and clinical
Besson *et al.*[5]	HL	2007	France	Unknown	Case-control	Family and population	Human genetic
Delahaye-Sourdeix *et al.*[6]	HL	2015	France, Germany, Spain, Czech Republic, Ireland, UK, Denmark, Sweden, The Netherlands; unknown	1987-2009	Case-control	Population, primary care, hospital	Human genetic
Diepstra *et al.*[7]	HL	2005	The Netherlands	1987-2000	Case-control	Family	Human genetic
Epstein *et al.*[8]	HL	2015	USA	1997-2000	Case-control	Population	Diet
Farrell *et al.*[9]	HL	2010	Unknown	Unknown	Case-control	Unknown	Infection and clinical
Gao *et al.*[10]	HL	2013	USA	1997-2000	Case-control	Population	Diet
Glaser *et al.*[11]	HL	2004	USA	1988-1994	Case-control	Population (female)	Environmental and non-diet lifestyle
Glaser *et al.*[12]	HL	2005	USA	1988-1994	Case-control	Population (female)	Environmental and non-diet lifestyle; Infection and clinical
Hjalgrim *et al.*[13]	HL	2007	Denmark, Sweden	1999-2002	Case-control	Population	Environmental and non-diet lifestyle
Hjalgrim *et al.*[14]	HL	2007	Denmark, Sweden	1999-2002	Case-control	Population	Environmental and non-diet lifestyle; Infection and clinical
Hollander *et al.*[15]	HL	2015	Denmark, Sweden	1999-2002	Case-control	Population	Infection and clinical
Jarrett *et al.*[16]	HL	2010	Unknown	Unknown	Case-control	Unknown	Human genetic
Johnson *et al.*[17]	HL	2015	UK	Unknown	Case-control	Population	Human genetic
Keegan *et al.*[18]	HL	2006	USA	1988-1994	Case-control	Population (female)	Infection and clinical
Li *et al.*[19]	HL	2013	USA	1997-2000	Case-control	Population	Infection and clinical
Linabery *et al.*[20]	HL	2014	Canada, Puerto Rico, USA	1989-2003	Case-control and reconstructed cohort	Population (children)	Infection and clinical
Linabery *et al.*[21]	HL	2015	Canada, Puerto Rico, USA	1989-2003	Case-control	Population (children)	Infection and clinical
Niens *et al.*[22]	HL	2006	The Netherlands	1987-2000	Case-control	Family	Human genetic

Niens *et al.*[23]	HL	2007	The Netherlands, UK	1987-2000	Case-control	Family, blood donor	Human genetic
Urayama *et al.*[24]	HL	2012	France, Germany, Spain, Czech Republic, Ireland, UK, Denmark, Sweden, The Netherlands, unknown	1987-2009	Case-control	Population, primary care, hospital	Human genetic
Willett *et al.*[25]	HL	2006	UK	1998-2003	Case-control	Population	Infection and clinical
Willett *et al.*[26]	HL	2007	UK	1998-2003	Case-control	Population	Diet; Environmental and non-diet lifestyle
Levine *et al.*[27]	GC	1995	USA	1967-1993	Case-control	Population (male)	Infection and clinical
Armstrong *et al.*[28]	NPC	1983	Malaysia	1973-1980	Case-control	Population	Diet
Armstrong *et al.*[29]	NPC	1983	Malaysia	1973-1980	Case-control	Population	Diet; Environment and non-diet lifestyle; Infection and clinical
Armstrong *et al.*[30]	NPC	1998	Malaysia	1990-1992	Case-control	Population	Diet
Armstrong *et al.*[31]	NPC	2000	Malaysia	1990-1992	Case-control	Population	Environment and non-diet lifestyle
Bei *et al.*[32]	NPC	2010	China	2002-2009	Case-control	Population (some from another country)	Human genetic
Bel Hadj Jrad *et al.*[33]	NPC	2006	Tunisia	1991-2003	Case-control	Blood donors	Human genetic
Ben Chaaben *et al.*[34]	NPC	2011	Tunisia	Unknown	Case-control	Population	Human genetic
Ben Chaaben *et al.*[35]	NPC	2015	Tunisia	Unknown	Case-control	Unknown	Human genetic
Ben Nasr *et al.*[36]	NPC	2007	Tunisia	Unknown	Case-control	Blood donors	Human genetic
Ben Nasr *et al.*[37]	NPC	2008	Tunisia	Unknown	Case-control	Blood donors	Human genetic
Ben Nasr *et al.*[38]	NPC	2009	Tunisia	Unknown	Case-control	Blood donors	Human genetic
Ben Nasr *et al.*[39]	NPC	2010	Tunisia	Unknown	Case-control	Blood donors	Human genetic
Bendjemana *et al.*[40]	NPC	2006	Tunisia	Unknown	Case-control	Family and professional	Human genetic
Birgander *et al.*[41]	NPC	1996	China	Unknown	Case-control	Blood donors	Human genetic
Bogger-Goren *et al.*[42]	NPC	1987	Israel	1974-1982	Case-control	Unknown	Infection and clinical
Bray *et al.*[43]	NPC	2008	12 countries^	1983-1997	Cohort/longitudinal	National	Environment and non-diet lifestyle
Broehm *et al.*[44]	NPC	2017	USA	1992-2013	Cohort/longitudinal	Region	Environment and non-diet lifestyle
Burt *et al.*[45]	NPC	1992	USA	1973-1986	Cohort/longitudinal	Global	Environment and non-diet lifestyle
Burt *et al.*[46]	NPC	1994	USA	1987-1991	Case-control	Population	Human genetic
Burt *et al.*[47]	NPC	1996	USA	1987-1994	Case-control	Population	Human genetic
Butsch Kovacic *et al.*[48]	NPC	2005	Taiwan	1991-1994	Case-control	Population	Human genetic
Cao *et al.*[49]	NPC	2006	China	2000-2003	Case-control	Population	Human genetic
Cao *et al.*[50]	NPC	2010	China	2001-2004	Case-control	Population	Human genetic
Cao *et al.*[51]	NPC	2011	China	1987-2007	Cohort/longitudinal	Region	Infection and clinical

Cao *et al.*[52]	NPC	2010	China	2000-2004	Case-control	Population	Human genetic
Chan *et al.*[53]	NPC	1983	Singapore	1973-1981	Case-control	Blood donors (including cord blood), staff, family	Human genetic
Chan *et al.*[54]	NPC	1985	Malaysia, Singapore	Unknown	Case-control	Population	Human genetic
Chan *et al.*[55]	NPC	1991	USA	1963-1980	Case-control	Hospital	Infection and clinical
Chelleng *et al.*[56]	NPC	2000	India	1996-1997	Case-control	Population	Diet; Environment and non-diet lifestyle; Infection and clinical
Chen *et al.*[57]	NPC	1990	Taiwan	1986-1987	Case-control	Population	Diet; Environment and non-diet lifestyle; Infection and clinical
Chen *et al.*[58]	NPC	1997	China	1992-1993	Case-control	Population	Diet; Environment and non-diet lifestyle; Infection and clinical
Chen *et al.*[59]	NPC	2012	China	1992-2007	Cohort/longitudinal	Region	Infection and clinical
Cheng *et al.*[60]	NPC	1999	Taiwan	1998-1999	Case-control	Population	Diet; Environment and non-diet lifestyle
Cheng *et al.*[61]	NPC	2003	Taiwan	1991-1994	Case-control	Population	Human genetic
Chentoufi *et al.*[62]	NPC	2016	Saudi Arabia	Unknown	Case-control	Hospital	Human genetic
Chin *et al.*[63]	NPC	2015	Malaysia	Unknown	Case-control	Blood donors	Human genetic
Cho *et al.*[64]	NPC	2003	Taiwan	1991-1994	Case-control	Population	Human genetic
Chung *et al.*[65]	NPC	2014	Taiwan	2005-2011	Case-control	Medical insurance	Infection and clinical
Coghill *et al.*[66]	NPC	2016	Taiwan	1996-2010	Case-control	Family	Infection and clinical
Coghill *et al.*[67]	NPC	2016b	Taiwan	1996-2010	Case-control	Family	Infection and clinical
Cui *et al.*[68]	NPC	1982	China	Unknown	Case-control	Blood donor	Human genetic
Cui *et al.*[69]	NPC	2015	China	Unknown	Case-control	Unknown	Human genetic
Da *et al.*[70]	NPC	2002	China	Unknown	Case-control	Population	Human genetic
Dai *et al.*[71]	NPC	2007	China	2005-2006	Case-control	Population	Human genetic
Dai *et al.*[72]	NPC	2016	China	Unknown	Case-control	Family, blood donors, population, individuals with other conditions; many from different setting	Human genetic
Dardari *et al.*[73]	NPC	2001	Morocco	1997-1999	Case-control	Blood donors, staff, population	Human genetic
Delfitri *et al.*[74]	NPC	2011	Indonesia	Unknown	Case-control	Hospital	Human genetic
Deng *et al.*[75]	NPC	2002	China	Unknown	Case-control	Students	Human genetic
Deng *et al.*[76]	NPC	2004	China	2001-2002	Case-control	Population	Human genetic
Deng *et al.*[77]	NPC	2005	China	2001-2003	Case-control	Population	Human genetic

Douik *et al.*[78]	NPC	2009	Tunisia	Unknown	Case-control	Population	Human genetic
Douik *et al.*[79]	NPC	2016	Tunisia	Unknown	Case-control	Population	Human genetic
Ekburanawat *et al.*[80]	NPC	2010	Thailand	2007-2008	Case-control	Hospital	Diet; Environment and non-diet lifestyle; Infection and clinical
Fachiroh *et al.*[81]	NPC	2012	Thailand	2005-2010	Case-control	Hospital	Diet; Environment and non-diet lifestyle; Human genetic
Fan *et al.*[82]	NPC	2005	China	Unknown	Case-control	Hospital	Human genetic
Fan *et al.*[83]	NPC	2006	China	2000-2004	Case-control	Hospital	Human genetic
Fan *et al.*[84]	NPC	2013	China	2003-2004	Case-control	Population	Human genetic
Farhat *et al.*[85]	NPC	2008	Tunisia	1998-unknown	Case-control	Blood donors	Human genetic
Farhat *et al.*[86]	NPC	2008	Tunisia	1998-unknown	Case-control	Blood donors	Human genetic
Farrow *et al.*[87]	NPC	1998	USA	1987-1993	Case-control	Population	Diet
Feng *et al.*[88]	NPC	2002	China	Unknown	Case-control	Family	Human genetic
Feng *et al.*[89]	NPC	2007	Algeria, Morocco, Tunisia	2002-2005	Case-control	Hospital	Diet
Feng *et al.*[90]	NPC	2008	China	2005-2007	Case-control	Blood donors	Human genetic
Feng *et al.*[91]	NPC	2009	Algeria, Morocco, Tunisia	2002-2005	Case-control	Hospital	Diet; Environment and non-diet lifestyle
Friborg *et al.*[92]	NPC	2005	Greenland	1973-2002	Cohort/longitudinal	National	Infection and clinical
Friborg *et al.*[93]	NPC	2007	Singapore	1993-2005	Cohort/longitudinal	Region	Diet; Environment and non-diet lifestyle
Gao *et al.*[94]	NPC	2008	China	2005-2007	Case-control	Hospital	Human genetic
Gao *et al.*[95]	NPC	2009	China	2005-2008	Case-control	Hospital	Human genetic
Gao *et al.*[96]	NPC	2012	China	Unknown	Case-control	Unknown	Human genetic
Gao *et al.*[97]	NPC	2015	China	Unknown	Case-control	Unknown	Human genetic
Ghandri *et al.*[98]	NPC	2011	Tunisia	Unknown	Case-control	Blood donors	Human genetic
Golovleva *et al.*[99]	NPC	1997	China	Unknown	Case-control	Blood donors	Human genetic
Guo *et al.*[100]	NPC	2006	China	Unknown	Case-control	Population	Human genetic
Guo *et al.*[101]	NPC	2008	China	2000-2001	Case-control	Family, population	Human genetic
Guo *et al.*[102]	NPC	2009	China	2004-2005	Case-control	Population	Diet; Environment and non-diet lifestyle; Infection and clinical
Guo *et al.*[103]	NPC	2010	China	2000-2005	Case-control	Family, population	Human genetic
Guo *et al.*[104]	NPC	2014	China	2000-2001 and 2004-2005	Case-control	Population	Human genetic
Hadhri-Guiga *et al.*[105]	NPC	2007	Tunisia	Unknown	Case-control	Population	Human genetic
Hall *et al.*[106]	NPC	1982	Kenya, Tanzania	Unknown	Case-control	Hospital, population	Human genetic
Hashim *et al.*[107]	NPC	2012	Malaysia	Unknown	Case-control	Hospital	Diet; Environment and non-diet lifestyle; Human genetic
Hassen *et al.*[108]	NPC	2007	Tunisia	Unknown	Case-control	Blood donors	Human genetic

Hassen *et al.*[109]	NPC	2011	Tunisia	Unknown	Case-control	Blood donors	Human genetic
Hawkins *et al.*[110]	NPC	1974	China, Malaysia, Singapore	Unknown	Case-control	Hospital, blood donors	Human genetic
He *et al.*[111]	NPC	2005	China	2000-2003	Case-control	Blood donors, staff	Human genetic
He *et al.*[112]	NPC	2007	China	2003-2004	Case-control	Population	Human genetic
He *et al.*[113]	NPC	2009	China	Unknown	Case-control	Blood donors	Human genetic
Henderson *et al.*[114]	NPC	1976	USA	1960-1974	Case-control	Hospital	Diet; Environment and non-diet lifestyle; Infection and clinical
Hildesheim *et al.*[115]	NPC	1992	The Philippines	Unknown	Case-control	Hospital, population	Infection and clinical
Hildesheim *et al.*[116]	NPC	1995	Taiwan	1991-1994	Case-control	Population	Human genetic
Hildesheim *et al.*[117]	NPC	1997	Taiwan	1991-1994	Case-control	Population	Human genetic
Hildesheim *et al.*[118]	NPC	2001	Taiwan	1991-1994	Case-control	Population	Environment and non-diet lifestyle
Hildesheim *et al.*[119]	NPC	2002	Taiwan	1991-1994	Case-control	Population	Human genetic
Hildesheim *et al.*[120]	NPC	1991	The Philippines	Unknown	Case-control	Unknown	Infection and clinical
Hirankarn *et al.*[121]	NPC	2004	Thailand	1994-2001	Case-control	Blood donors	Human genetic
Hirunsatit *et al.*[122]	NPC	2003	Thailand	1994-2001	Case-control	Blood donors	Human genetic
Ho *et al.*[123]	NPC	2006	Taiwan	2000-2004	Case-control	Hospital	Human genetic
Hsu *et al.*[124]	NPC	2009	Taiwan	1984-2006	Cohort/longitudinal	Region (male)	Environment and non-diet lifestyle; Infection and clinical
Hsu *et al.*[125]	NPC	2011	Taiwan	1984-2008	Cohort/longitudinal	Region (male)	Environment and non-diet lifestyle; Infection and clinical
Hsu *et al.*[126]	NPC	2012	Taiwan	1991-1994	Case-control	Population	Diet
Hsu *et al.*[127]	NPC	2015	Taiwan	2010-2014	Case-control	Population (male)	Diet; Environment and non-diet lifestyle; Infection and clinical
Hsu *et al.*[128]	NPC	2016	Taiwan	2010-2014	Case-control	Unknown	Diet; Environment and non-diet lifestyle; Infection and clinical
Hu *et al.*[129]	NPC	2012	China	2001-2007	Case-control	Population	Human genetic
Huang *et al.*[130]	NPC	2011	China	2005-2007	Case-control	Hospital	Human genetic
Hung *et al.*[131]	NPC	2014	Taiwan	2002-2011	Case-control	Medical insurance	Infection and clinical
Jalbout *et al.*[132]	NPC	2003	Tunisia	Unknown	Case-control	Population	Human genetic
Jeannel *et al.*[133]	NPC	1990	Tunisia	1986-1987	Case-control	Population	Diet; Infection and clinical
Ji *et al.*[134]	NPC	2011	China	1991-2009	Case-control	Population	Diet; Environment and non-diet lifestyle; Infection and clinical
Jia *et al.*[135]	NPC	2004	China	1999-2001	Cohort/longitudinal	Family, region	Infection and clinical
Jia *et al.*[136]	NPC	2009	China	2001-2004 and 2005-2007	Case-control	Family, hospital	Human genetic
Jia *et al.*[137]	NPC	2010	China	2005-2007	Case-control	Hospital	Diet
Jiang *et al.*[138]	NPC	2011	China	2006-2010	Case-control	Hospital	Human genetic
Jing *et al.*[139]	NPC	1977	USA	1960-1974	Case-control	Hospital	Human genetic
Ko *et al.*[140]	NPC	2014	China	2009-2012	Case-control	Population	Human genetic

Kongruttanachok *et al.*[141]	NPC	2001	Thailand	Unknown	Case-control	Blood donors	Human genetic
Laantri *et al.*[142]	NPC	2011	Algeria, Morocco, Tunisia	Unknown	Case-control	Hospital	Human genetic
Lakhanpal *et al.*[143]	NPC	2015	India	2010-2013	Case-control	Hospital	Human genetic
Lanier *et al.*[144]	NPC	1980	USA	1966-1976	Case-control	Population	Human genetic
Lanier *et al.*[145]	NPC	1980b	USA	1965-unknown	Case-control	Population	Infection and clinical
Laouamri *et al.*[146]	NPC	2000	Algeria	1994-1997	Case-control	Population	Infection and clinical
Laouamri *et al.*[147]	NPC	2001	Algeria	1994-1997	Case-control	Population	Diet; Environmental and non-diet lifestyle
Lee *et al.*[148]	NPC	1994	Singapore	1988-1990	Case-control	Hospital	Diet
Levine *et al.*[149]	NPC	1998	USA	1984-1988	Case-control	Population (male)	Infection and clinical
Li *et al.*[150]	NPC	1995	China	Unknown	Case-control	Unknown	Human genetic
Li *et al.*[151]	NPC	2006	China	1981-1998	Case-cohort	Occupational (female)	Environment and non-diet lifestyle
Li *et al.*[152]	NPC	2007	Tunisia	1991-2004	Case-control	Blood donor	Human genetic
Li *et al.*[153]	NPC	2012	China	2006-2010	Case-control	Hospital	Human genetic
Li *et al.*[154]	NPC	2013	China	Unknown	Case-control	Population	Human genetic
Lin *et al.*[155]	NPC	1973	Taiwan	1969-1971	Case-control	Population	Environment and non-diet lifestyle; Infection and clinical
Lin *et al.*[156]	NPC	1979	Taiwan	1969-1971	Case-control	Population	Environment and non-diet lifestyle; Infection and clinical
Lin *et al.*[157]	NPC	1986	Taiwan	1969-1971	Case-control	Population	Environment and non-diet lifestyle; Infection and clinical
Lin *et al.*[158]	NPC	1997	Taiwan	1985-1993	Cohort	Population (male)	Diet; Environment and non-diet lifestyle; Infection and clinical
Liu *et al.*[159]	NPC	2015	Sweden	1961-2009	Cohort/longitudinal	Family and national	Infection and clinical
Liu *et al.*[160]	NPC	2015	Taiwan	2003-2009	Case-control	Hospital	Human genetic
Liu *et al.*[161]	NPC	2015	China	2001-2010	Case-control	Hospital, population	Human genetic
Liu *et al.*[162]	NPC	2016	Sweden	1961-2009	Case-control	Population	Environment and non-diet lifestyle
Liu *et al.*[163]	NPC	2016	Taiwan	1980-2011	Cohort/longitudinal	Family	Environment and non-diet lifestyle
Liu *et al.*[164]	NPC	2016	China	2010-2014	Case-control	Population	Infection and clinical
Lo *et al.*[165]	NPC	2016	Taiwan	1991-1994	Case-control	Population	Diet
Lourembam *et al.*[166]	NPC	2015	India	2011-2013	Case-control	Unknown	Diet; Environment and non-diet lifestyle; Human genetic
Lu *et al.*[167]	NPC	2003	Taiwan	1998-1999	Case-control	Blood and organ donors, staff	Human genetic
Mabuchi *et al.*[168]	NPC	1985	USA	Unknown	Case-control	Hospital	Environment and non-diet lifestyle
Mazzoni *et al.*[169]	NPC	2016	Italy	2010-2013	Case-control	Population	Infection and clinical
Mirabelli *et al.*[170]	NPC	2000	USA	Unknown	Case-control	Population (male)	Environment and non-diet lifestyle

Mo *et al.*[171]	NPC	2009	China	2006	Case-control	Unknown	Infection and clinical
Mokni-Baizig *et al.*[172]	NPC	2001	Tunisia	Unknown	Case-control	Population	Human genetic
Moumad *et al.*[173]	NPC	2013	Morocco, Tunisia	2001-2004 and 2006-2009	Case-control	Hospital	Human genetic
Mousavi *et al.*[174]	NPC	2010	Sweden	1932-2006	Cohort/longitudinal	Family and national	Environment and non-diet lifestyle
Nam *et al.*[175]	NPC	1992	USA	Unknown	Case-control	Population (Caucasian)	Diet; Environment and non-diet lifestyle
Nazar-Stewart *et al.*[176]	NPC	1999	USA	1987-1993	Case-control	Population	Human genetic
Nesic *et al.*[177]	NPC	2010	Serbia	2001-2003	Case-control	Hospital	Diet; Environment and non-diet lifestyle; Infection and clinical
Ng *et al.*[178]	NPC	2009	Malaysia	Unknown	Case-control	Hospital	Human genetic
Ning *et al.*[179]	NPC	1990	China	1985-1986	Case-control	Population	Diet; Environment and non-diet lifestyle; Infection and clinical
Nong *et al.*[180]	NPC	2009	China	2007-2008	Case-control	Blood donors, staff	Human genetic
Ooi *et al.*[181]	NPC	1997	Singapore	Unknown	Case-control	Population	Human genetic
Ou *et al.*[182]	NPC	1985	China	Unknown	Case-control	Blood donors	Human genetic
Pasini *et al.*[183]	NPC	2009	Italy	1994-2005	Case-control	Organ donors	Human genetic
Polesel *et al.*[184]	NPC	2011	Italy	1992-2008	Case-control	Hospital (Caucasian)	Diet; Environment and non-diet lifestyle
Pratesi *et al.*[185]	NPC	2006	Italy	Unknown	Case-control	Population	Human genetic
Qin *et al.*[186]	NPC	2011	China	2002-2009	Case-control	Hospital, population	Human genetic
Qiu *et al.*[187]	NPC	2015	China	2002-2010	Case-control	Hospital, population	Human genetic
Ren *et al.*[188]	NPC	2005	China	Unknown	Case-control	Students	Human genetic
Ren *et al.*[189]	NPC	2010	China	2005-2007	Case-control	Hospital	Diet; Environment and non-diet lifestyle; Infection and clinical
Ruan *et al.*[190]	NPC	2010	China	2005-2007	Case-control	Hospital	Diet
Ruan *et al.*[191]	NPC	2013	China	2005-2007	Case-control	Hospital	Environment and non-diet lifestyle; Human genetic; Infection and clinical
Shanmugaratnum *et al.*[192]	NPC	1978	Singapore	1966-1968	Case-control	Hospital	Environment and non-diet lifestyle
Shao *et al.*[193]	NPC	2011	China	2000-2003	Case-control	Population	Human genetic
Sheng *et al.*[194]	NPC	2013	China	2004-2011	Case-control	Population	Human genetic
Shield *et al.*[195]	NPC	2017	Global	2012	Cohort/longitudinal	National	Environment and non-diet lifestyle
Shih *et al.*[196]	NPC	2012	Taiwan	2003-2009	Case-control	Hospital	Human genetic
Simons *et al.*[197]	NPC	1974	Singapore	Unknown	Case-control	Hospital, unknown	Human genetic
Simons *et al.*[198]	NPC	1974	Singapore	Unknown	Case-control	Hospital, unknown	Human genetic
Simons *et al.*[199]	NPC	1975	Singapore	Unknown	Case-control	Hospital, pregnant women	Human genetic

Simons *et al.*[200]	NPC	1975	Singapore	Unknown	Case-control	Unknown	Human genetic
Song *et al.*[201]	NPC	2006	China	2000-2004	Case-control	Population (Caucasian)	Human genetic
Sousa *et al.*[202]	NPC	2006	Portugal	Unknown	Case-control	Population	Human genetic
Sousa *et al.*[203]	NPC	2011	Portugal	2002-2007	Case-control	Blood donors	Human genetic
Sousa *et al.*[204]	NPC	2011	Portugal	Unknown	Case-control	Blood donors (Caucasian)	Human genetic
Sousa *et al.*[205]	NPC	2013	Portugal	Unknown	Case-control	Blood donors (Caucasian)	Human genetic
Sousa *et al.*[206]	NPC	2016	Portugal	Unknown	Case-control	Blood donors (Caucasian)	Human genetic
Sousa *et al.*[207]	NPC	2016	Portugal	Unknown	Case-control	Blood donors (Caucasian)	Human genetic
Sriamporn *et al.*[208]	NPC	1992	Thailand	1990	Case-control	Hospital	Diet; Environment and non-diet lifestyle
Su *et al.*[209]	NPC	2011	Taiwan	Unknown	Case-control	Unknown	Human genetic
Sui *et al.*[210]	NPC	2010	China	2004-2008	Case-control	Population	Human genetic
Tai *et al.*[211]	NPC	2007	China	Unknown	Case-control	Unknown	Human genetic
Tang *et al.*[212]	NPC	2010	China	Unknown	Case-control	Family, population	Human genetic
Tang *et al.*[213]	NPC	2012	China	2000-2001 and 2004-2005	Case-control	Family, population, unknown	Human genetic
Tian *et al.*[214]	NPC	2006	China	Unknown	Case-control	Unknown	Human genetic
Tiwawech *et al.*[215]	NPC	2003	Thailand	Unknown	Case-control	Hospital	Human genetic
Tiwawech *et al.*[216]	NPC	2005	Thailand	Unknown	Case-control	Hospital	Human genetic
Tsai *et al.*[217]	NPC	2013	Taiwan	2003-2009	Case-control	Hospital	Human genetic
Tse *et al.*[218]	NPC	2009	Taiwan	1984-2008	Case-control	Hospital	Human genetic
Tsou *et al.*[219]	NPC	2011	Taiwan	2003-2009	Case-control	Hospital	Human genetic
Turkoz *et al.*[220]	NPC	2011	Turkey	Unknown	Case-control	Hospital	Diet; Environment and non-diet lifestyle; Human genetic; Infection and clinical
Ung *et al.*[221]	NPC	1999	Taiwan	1991-1994	Case-control	Population	Infection and clinical
Vaughan *et al.*[222]	NPC	1996	USA	1987-1993	Case-control	Population	Diet; Environment and non-diet lifestyle; Infection and clinical
Vaughan *et al.*[223]	NPC	2000	USA	1987-1993	Case-control	Population	Environment and non-diet lifestyle
Wang *et al.*[224]	NPC	2010	China	2007-2008	Case-control	Hospital	Human genetic
Ward *et al.*[225]	NPC	2000	Taiwan	1991-1994	Case-control	Population	Diet
Wei *et al.*[226]	NPC	2007	China	2005-2006	Case-control	Hospital	Human genetic
Wei *et al.*[227]	NPC	2007	China	2005-2006	Case-control	Blood donors, staff	Human genetic
Wei *et al.*[228]	NPC	2007	China	2005-2006	Case-control	Hospital	Human genetic
Wei *et al.*[229]	NPC	2009	China	2005-2006	Case-control	Hospital	Human genetic
Wei *et al.*[230]	NPC	2010	China	2007-2008	Case-control	Hospital	Human genetic
Wei *et al.*[231]	NPC	2012	China	Unknown	Case-control	Hospital	Human genetic

West *et al.*[232]	NPC	1993	The Philippines	Unknown	Case-control	Hospital, population	Environment and non-diet lifestyle
Wu *et al.*[233]	NPC	1989	Taiwan	Unknown	Case-control	Hospital	Human genetic
Xiao *et al.*[234]	NPC	2010	China	2001-2007	Case-control	Population	Human genetic
Xiao *et al.*[235]	NPC	2010	China	2001-2007	Case-control	Population	Human genetic
Xiao *et al.*[236]	NPC	2013	China	2001-2009	Case-control	Population	Human genetic
Xie *et al.*[237]	NPC	2015	China	2010-2012	Case-control	Hospital	Environment and non-diet lifestyle; Infection and clinical
Xiong *et al.*[238]	NPC	2004	China	Unknown	Case-control	Family	Human genetic
Xu *et al.*[239]	NPC	2010	China	2005-2006	Case-control	Hospital	Human genetic
Xu *et al.*[240]	NPC	2012	China	2005-2007	Case-control	Hospital	Diet; Environment and non-diet lifestyle; Infection and clinical
Yang *et al.*[241]	NPC	2005	Taiwan	1996-unknown	Case-control	Family	Diet; Human genetic; Environment and non-diet lifestyle
Yang *et al.*[242]	NPC	2007	China	2005-2006	Case-control	Hospital	Human genetic
Yang *et al.*[243]	NPC	2008	China	2005-2006	Case-control	Hospital	Human genetic
Yang *et al.*[244]	NPC	2009	China	2005-2006	Case-control	Hospital	Human genetic
Yang *et al.*[245]	NPC	2011	China	2007-2009	Case-control	Hospital	Human genetic
Yang *et al.*[246]	NPC	2014	China	2002-2010 and 2001-2009	Case-control	Hospital, population	Human genetic
Ye *et al.*[247]	NPC	2015	China	2008-2013	Case-control	Hospital, population	Infection and clinical
Yew *et al.*[248]	NPC	2012	Malaysia	Unknown	Case-control	Blood donors	Human genetic
Yu *et al.*[249]	NPC	1986	China	1981-unknown	Case-control	Friends	Diet
Yu *et al.*[250]	NPC	1988	China	1984-1987	Case-control	Population	Diet; Environment and non-diet lifestyle; Infection and clinical
Yu *et al.*[251]	NPC	1989	China	1983-1987	Case-control	Population	Diet
Yu *et al.*[252]	NPC	1990	China	1983-1987	Case-control	Population	Environment and non-diet lifestyle; Infection and clinical
Yu *et al.*[253]	NPC	2009	Taiwan	1980-2003	Case-control	Family. Population	Human genetic
Yuan *et al.*[254]	NPC	2000	China	1988-1991	Case-control	Population	Environment and non-diet lifestyle; Infection and clinical
Zeng *et al.*[255]	NPC	2016	China	2009-2011	Case-control	Hospital	Diet
Zhao *et al.*[256]	NPC	2012	China	2005-2008	Case-control	Hospital	Human genetic
Zheng *et al.*[257]	NPC	1994	China	1986-unknown	Case-control	Population	Diet; Environment and non-diet lifestyle
Zheng *et al.*[258]	NPC	1994	China	1985-1989	Case-control	Friends, population	Diet; Infection and clinical
Zheng *et al.*[259]	NPC	2007	China	Unknown	Case-control	Hospital	Human genetic
Zheng *et al.*[260]	NPC	2011	China	2001-2009	Case-control	Hospital, population	Human genetic

Zheng *et al.*[261]	NPC	2012	China	2001-2009	Case-control	Hospital, population	Human genetic
Zheng *et al.*[262]	NPC	2015	China	Unknown	Case-control	Unknown	Human genetic
Zhou *et al.*[263]	NPC	2006	China	2003-2004	Case-control	Hospital	Human genetic
Zhou *et al.*[264]	NPC	2007	China	2003-2005	Case-control	Population	Human genetic
Zhou *et al.*[265]	NPC	2007	China	2003-2005	Case-control	Blood donors, population, staff	Human genetic
Zhou *et al.*[266]	NPC	2009	China	2006-2008	Case-control	Hospital	Human genetic
Zhu *et al.*[267]	NPC	1990	China	Unknown	Case-control	Unknown	Human genetic
Zhu *et al.*[268]	NPC	1995	USA	1984-1988	Case-control	Population (male)	Environment and non-diet lifestyle
Zhu *et al.*[269]	NPC	2008	China	Unknown	Case-control	Hospital	Human genetic
Zhu *et al.*[270]	NPC	2010	China	2008-2009	Case-control	Blood donors	Human genetic
Zong *et al.*[271]	NPC	1992	China	1986-1987	Cohort/longitudinal	Region	Environment and non-diet lifestyle

Table S2: Quality assessment

BL- Burkitt's lymphoma, GC- gastric carcinoma, HL- Hodgkin's lymphoma, N- no, N/A- not applicable, NPC- nasopharyngeal carcinoma, U- unknown, Y- yes

Author	Cancer	Is the study design clearly reported?	Is the hypothesis/aim/objective of the study clearly described?	Are the main outcomes to be measured clearly described in the Introduction or Methods section?	Are the characteristics of the patients included in the study clearly described?	Are the distributions of principal confounders in each group of subjects to be compared clearly described?	Are the main findings of the study clearly described?	Does the study provide estimates of the random variability in the data for the main outcomes?	Have the characteristics of patients lost to follow-up been described?	Have actual probability values been reported for the main outcomes except where the probability value is <0.001?	Was there potential for recall bias in the ascertainment of the exposure?	Was there potential for differential or non-differential misclassification of the exposure?	Was there potential for observer bias in ascertainment of the outcome?	Was there potential for differential or non-differential misclassification of the outcome?	If any of the results of the study were based on 'data dredging' was this made clear?	In trials and cohort studies, do the analyses adjust for different lengths of follow-up of patients, or in case-control studies, is the time period between the intervention and outcome the same for cases and controls?	Were the statistical tests used to assess the main outcomes appropriate?	Were the main outcome measures used accurate (valid and reliable)?	Were the patients in different intervention groups (trials and cohort studies) or were the cases and controls (case-control studies) recruited from the same population?	Were study subjects in different intervention groups (trials and cohort studies) or were the cases and controls (case-control studies) recruited over the same period of time?	Was there adequate adjustment for confounding in the analyses of interest?	Were losses of patients to follow-up taken into account?	Are the study results appropriately interpreted e.g. in terms of the strength of the evidence, its application/implications, causality?	Did the study have sufficient power to detect a clinically important effect where the probability value for a difference being due to chance is less than 5%?
de-Thé *et al.*[1]	BL	Y	Y	Y	Y	N	Y	N	N/A	Y	N	N	N	N	N/A	U	N	Y	Y	Y	N	N/A	Y	0
Geser *et al.*[2]	BL	Y	Y	Y	Y	N	Y	N	N/A	Y	N	N	N	N	N/A	U	N	Y	Y	Y	N	N/A	Y	0
Alexander *et al.*[3]	HL	Y	N	Y	Y	N	N	Y	N/A	Y	Y	Y	N	N	N/A	U	Y	Y	Y	N	N	N/A	Y	0
Alexander *et al.*[4]	HL	Y	Y	Y	Y	N	Y	Y	N/A	Y	Y	Y	N	N	N/A	U	N	Y	Y	Y	N	N/A	Y	4
Besson *et al.*[5]	HL	Y	Y	Y	Y	N	Y	Y	N/A	Y	N	N	N	N	N/A	U	Y	Y	Y	U	N	N/A	Y	4
Delahaye-Sourdeix *et al.*[6]	HL	Y	Y	Y	Y	N	Y	Y	N/A	Y	N	N	U	U	N/A	U	Y	U	N	U	N	N/A	Y	5
Diepstra *et al.*[7]	HL	Y	Y	Y	Y	N	Y	Y	N/A	Y	N	N	N	N	N/A	U	Y	Y	Y	U	N	N/A	N	4
Epstein *et al.*[8]	HL	Y	Y	Y	Y	Y	Y	Y	N/A	Y	Y	Y	N	N	N/A	U	Y	Y	Y	N	N	N/A	Y	3
Farrell *et al.*[9]	HL	N	Y	N	N	N	Y	Y	N/A	N	Y	Y	U	U	N/A	U	Y	N	N	N	N	N/A	Y	U
Gao *et al.*[10]	HL	Y	Y	Y	Y	N	Y	Y	N/A	Y	Y	Y	N	N	N/A	U	Y	Y	Y	N	Y	N/A	Y	3
Glaser *et al.*[11]	HL	Y	Y	Y	Y	N	Y	Y	N/A	Y	Y	Y	N	N	N/A	U	Y	Y	Y	N	Y	N/A	Y	0
Glaser *et al.*[12]	HL	Y	Y	Y	Y	Y	Y	Y	N/A	Y	Y	Y	N	N	N/A	U	Y	Y	Y	N	Y	N/A	Y	0
Hjalgrim *et al.*[13]	HL	Y	Y	Y	Y	N	Y	Y	N/A	Y	Y	Y	N	N	N/A	U	Y	Y	Y	Y	N	N/A	Y	5
Hjalgrim *et al.*[14]	HL	Y	Y	Y	Y	N	Y	Y	N/A	Y	Y	Y	N	N	N/A	U	Y	Y	Y	Y	N	N/A	Y	5
Hollander *et al.*[15]	HL	Y	Y	Y	Y	N	Y	Y	N/A	Y	Y	Y	N	N	N/A	Y	Y	Y	Y	Y	N	N/A	Y	5

Jarrett *et al.*[16]	HL	N	N	N	N	N	N	Y	N/A	N	N	N	U	U	N/A	U	Y	N	N	N	N	N/A	N	U
Johnson *et al.*[17]	HL	Y	Y	Y	Y	N	Y	Y	N/A	Y	N	N	N	N	N/A	U	N	Y	Y	N	N	N/A	Y	5
Keegan *et al.*[18]	HL	Y	Y	Y	Y	N	Y	Y	N/A	Y	Y	Y	N	N	N/A	U	Y	Y	Y	N	Y	N/A	Y	0
Li *et al.*[19]	HL	Y	Y	Y	Y	N	Y	N	N/A	N	Y	Y	N	N	N/A	U	Y	Y	Y	N	Y	N/A	Y	U
Linabery *et al.*[20]	HL	Y	Y	Y	Y	N	Y	Y	N/A	Y	Y	Y	N	N	N/A	U	Y	Y	Y	N	N	N/A	Y	3
Linabery *et al.*[21]	HL	N	Y	Y	Y	N	Y	Y	N/A	Y	Y	Y	N	N	N/A	U	Y	Y	Y	N	N	N/A	Y	3
Niens *et al.*[22]	HL	Y	Y	Y	Y	N	Y	Y	N/A	Y	N	N	N	N	N/A	U	N	Y	Y	U	N	N/A	Y	5
Niens *et al.*[23]	HL	Y	Y	Y	Y	N	Y	N	N/A	Y	N	N	N	N	N/A	U	N	Y	N	U	N	N/A	Y	5
Urayama *et al.*[24]	HL	Y	Y	Y	Y	N	Y	Y	N/A	Y	N	N	U	U	N/A	U	U	U	N	U	N	N/A	Y	5
Willett *et al.*[25]	HL	Y	Y	Y	N	N	N	Y	N/A	Y	Y	Y	N	N	N/A	U	Y	Y	Y	Y	N	N/A	Y	1
Willett *et al.*[26]	HL	Y	Y	Y	Y	N	Y	Y	N/A	N	Y	Y	N	N	N/A	U	Y	Y	Y	N	N	N/A	Y	1
Levine *et al.*[27]	GC	Y	Y	Y	Y	N	Y	Y	N/A	N	N	N	N	N	N/A	U	N	Y	Y	Y	N	N/A	Y	0
Armstrong *et al.*[28]	NPC	Y	Y	Y	Y	Y	Y	N	N/A	Y	Y	Y	N	N	N/A	Y	Y	Y	Y	U	N	N/A	Y	4
Armstrong *et al.*[29]	NPC	Y	Y	Y	Y	Y	Y	N	N/A	Y	Y	Y	N	N	N/A	Y	Y	Y	Y	U	N	N/A	Y	4
Armstrong *et al.*[30]	NPC	Y	Y	Y	Y	Y	Y	Y	N/A	Y	Y	Y	N	N	N/A	N	Y	Y	Y	U	N	N/A	Y	5
Armstrong *et al.*[31]	NPC	Y	Y	Y	Y	Y	Y	Y	N/A	Y	Y	Y	N	N	N/A	N	Y	Y	Y	U	N	N/A	Y	5
Bei *et al.*[32]	NPC	Y	Y	Y	Y	N	Y	Y	N/A	Y	N	N	N	N	N/A	U	Y	Y	N	Y	N	N/A	Y	5
Bel Hadj Jrad *et al.*[33]	NPC	Y	Y	Y	Y	N	Y	Y	N/A	Y	N	N	N	N	N/A	Y	N	Y	U	U	N	N/A	Y	5
Ben Chaaben *et al.*[34]	NPC	Y	Y	Y	Y	N	Y	Y	N/A	Y	N	N	N	N	N/A	Y	Y	Y	U	U	N	N/A	Y	5
Ben Chaaben *et al.*[35]	NPC	Y	Y	Y	Y	N	Y	Y	N/A	N	N	N	N	N	N/A	Y	Y	Y	U	U	N	N/A	Y	5
Ben Nasr *et al.*[36]	NPC	Y	Y	Y	Y	N	Y	Y	N/A	Y	N	N	N	N	N/A	Y	Y	Y	Y	U	N	N/A	Y	5
Ben Nasr *et al.*[37]	NPC	Y	Y	Y	Y	N	Y	Y	N/A	Y	N	N	N	N	N/A	Y	Y	Y	Y	U	N	N/A	Y	5
Ben Nasr *et al.*[38]	NPC	Y	Y	Y	Y	N	Y	Y	N/A	N	N	N	N	N	N/A	Y	Y	Y	Y	U	N	N/A	Y	5
Ben Nasr *et al.*[39]	NPC	Y	Y	Y	Y	N	Y	Y	N/A	Y	N	N	N	N	N/A	Y	Y	Y	Y	U	N	N/A	Y	5
Bendjemana *et al.*[40]	NPC	Y	Y	Y	Y	N	Y	N	N/A	N	N	N	N	N	N/A	Y	N	Y	Y	U	N	N/A	Y	1
Birgander *et al.*[41]	NPC	Y	Y	Y	N	N	Y	N	N/A	Y	N	N	U	U	N/A	U	N	U	U	U	N	N/A	Y	3
Bogger-Goren *et al.*[42]	NPC	Y	Y	Y	N	Y	Y	N	N/A	N	N	N	N	N	N/A	Y	N	Y	U	U	N	N/A	Y	0
Bray *et al.*[43]	NPC	Y	Y	Y	Y	N	Y	N	N/A	N	N	N	N	N	N/A	Y	Y	Y	Y	Y	N	N/A	Y	U
Broehm *et al.*[44]	NPC	Y	Y	Y	Y	N	Y	Y	N/A	N	N	N	N	N	N/A	Y	Y	Y	Y	Y	N	N/A	Y	U
Burt *et al.*[45]	NPC	Y	Y	Y	Y	Y	Y	N	N/A	Y	N	N	N	N	N/A	Y	Y	Y	N	Y	Y	N/A	Y	5
Burt *et al.*[46]	NPC	Y	Y	Y	Y	N	Y	Y	N/A	Y	N	N	N	N	N/A	U	N	Y	Y	U	N	N/A	N	1
Burt *et al.*[47]	NPC	Y	Y	Y	Y	N	Y	N	N/A	Y	N	N	N	N	N/A	U	Y	Y	Y	U	Y	N/A	Y	3
Butsch Kovacic *et al.*[48]	NPC	Y	Y	Y	Y	Y	Y	Y	N/A	Y	N	N	N	N	N/A	U	N	Y	Y	U	N	N/A	N	5
Cao *et al.*[49]	NPC	Y	Y	Y	Y	Y	Y	Y	N/A	Y	N	N	N	N	N/A	Y	N	Y	Y	U	Y	N/A	N	5
Cao *et al.*[50]	NPC	Y	Y	Y	Y	Y	Y	Y	N/A	Y	N	N	N	N	N/A	Y	N	Y	U	U	N	N/A	Y	5
Cao *et al.*[51]	NPC	Y	Y	Y	Y	Y	Y	Y	N	N	N	N	N	N	N/A	Y	Y	Y	Y	Y	Y	Y	Y	5
Cao *et al.*[52]	NPC	Y	Y	Y	Y	Y	Y	Y	N/A	Y	N	N	N	N	N/A	Y	N	Y	U	U	Y	N/A	Y	5
Chan *et al.*[53]	NPC	Y	Y	Y	N	Y	Y	N	N/A	Y	N	N	U	U	N/A	U	Y	U	U	U	N	N/A	Y	5
Chan *et al.*[54]	NPC	Y	Y	Y	Y	N	Y	N	N/A	Y	N	N	U	U	N/A	U	Y	U	N	U	N	N/A	Y	1

Chan *et al.*[55]	NPC	Y	Y	Y	Y	N	Y	Y	N/A	N	N	N	N	N	N/A	U	Y	Y	Y	Y	N	N/A	Y	0
Chelleng *et al.*[56]	NPC	Y	Y	Y	Y	N	Y	Y	N/A	Y	Y	Y	N	N	N/A	U	Y	Y	Y	U	N	N/A	Y	1
Chen *et al.*[57]	NPC	Y	Y	Y	Y	N	Y	Y	N/A	N	Y	Y	N	N	N/A	U	Y	Y	Y	U	N	N/A	Y	5
Chen *et al.*[58]	NPC	Y	Y	Y	Y	Y	Y	Y	N/A	Y	Y	N	N	N	N/A	Y	Y	Y	Y	Y	N	N/A	N	4
Chen *et al.*[59]	NPC	Y	Y	Y	Y	N	Y	Y	N	N	N	N	N	N	N/A	Y	Y	Y	Y	Y	N	N	Y	5
Cheng *et al.*[60]	NPC	Y	Y	Y	Y	Y	Y	Y	N/A	Y	Y	Y	N	N	N/A	Y	Y	Y	Y	U	Y	N/A	N	5
Cheng *et al.*[61]	NPC	Y	Y	Y	N	Y	Y	Y	N/A	N	N	N	N	N	N/A	U	N	Y	U	U	Y	N/A	Y	5
Chentoufi *et al.*[62]	NPC	Y	Y	Y	N	N	Y	N	N/A	Y	N	N	N	N	N/A	U	Y	Y	U	U	N	N/A	Y	1
Chin *et al.*[63]	NPC	Y	Y	Y	Y	N	Y	Y	N/A	Y	N	N	N	N	N/A	N	Y	Y	N	U	Y	N/A	Y	5
Cho *et al.*[64]	NPC	Y	Y	Y	Y	Y	Y	Y	N/A	N	N	N	N	N	N/A	Y	N	Y	Y	U	Y	N/A	Y	5
Chung *et al.*[65]	NPC	Y	Y	Y	Y	N	Y	Y	N/A	Y	N	N	N	N	N/A	U	Y	Y	Y	Y	N	N/A	Y	5
Coghill *et al.*[66]	NPC	Y	Y	Y	Y	N	Y	N	N/A	Y	N	N	U	U	N/A	U	Y	U	Y	Y	N	N/A	N	0
Coghill *et al.*[67]	NPC	Y	Y	Y	Y	N	Y	Y	N/A	Y	N	N	U	U	N/A	Y	Y	U	Y	Y	N	N/A	Y	0
Cui *et al.*[68]	NPC	Y	Y	Y	N	N	Y	N	N/A	N	N	N	N	N	N/A	U	Y	Y	N	U	N	N/A	Y	0
Cui *et al.*[69]	NPC	Y	Y	Y	N	N	Y	Y	N/A	N	N	N	N	N	N/A	U	Y	Y	U	U	N	N/A	Y	5
Da *et al.*[70]	NPC	Y	Y	Y	Y	Y	Y	Y	N/A	N	N	N	N	N	N/A	Y	Y	Y	Y	U	N	N/A	Y	3
Dai *et al.*[71]	NPC	Y	Y	Y	N	N	N	Y	N/A	Y	N	N	N	N	N/A	Y	N	Y	N	U	N	N/A	Y	5
Dai *et al.*[72]	NPC	Y	Y	Y	N	N	Y	N	N/A	N	N	N	N	N	N/A	U	U	Y	U	U	N	N/A	Y	5
Dardari *et al.*[73]	NPC	Y	Y	Y	Y	N	Y	N	N/A	Y	N	N	N	N	N/A	Y	Y	Y	N	U	N	N/A	Y	5
Delfitri *et al.*[74]	NPC	Y	Y	Y	Y	Y	Y	Y	N/A	Y	N	N	N	N	N/A	Y	Y	Y	Y	U	N	N/A	Y	2
Deng *et al.*[75]	NPC	Y	Y	Y	Y	N	Y	Y	N/A	Y	N	N	N	N	N/A	N	Y	Y	N	U	N	N/A	Y	3
Deng *et al.*[76]	NPC	Y	Y	Y	N	N	Y	N	N/A	N	N	N	N	N	N/A	U	Y	Y	N	U	N	N/A	Y	4
Deng *et al.*[77]	NPC	Y	Y	Y	N	N	Y	N	N/A	N	N	N	N	N	N/A	U	N	Y	U	U	N	N/A	Y	5
Douik *et al.*[78]	NPC	Y	Y	Y	Y	N	Y	Y	N/A	Y	N	N	N	N	N/A	N	Y	Y	U	U	N	N/A	Y	5
Douik *et al.*[79]	NPC	Y	Y	Y	Y	N	Y	Y	N/A	Y	N	N	N	N	N/A	N	Y	Y	U	U	N	N/A	N	5
Ekburanawat *et al.*[80]	NPC	Y	Y	Y	Y	Y	Y	Y	N/A	N	Y	Y	N	N	N/A	Y	Y	Y	Y	Y	N	N/A	Y	5
Fachiroh *et al.*[81]	NPC	Y	Y	Y	Y	Y	Y	Y	N/A	Y	Y	Y	N	N	N/A	Y	Y	Y	N	Y	N	N/A	Y	5
Fan *et al.*[82]	NPC	Y	Y	Y	N	N	Y	Y	N/A	N	N	N	N	N	N/A	U	Y	Y	Y	U	N	N/A	Y	5
Fan *et al.*[83]	NPC	Y	Y	Y	Y	Y	Y	Y	N/A	Y	N	N	N	N	N/A	Y	N	Y	N	U	N	N/A	N	5
Fan *et al.*[84]	NPC	Y	Y	Y	Y	Y	Y	Y	N/A	Y	N	N	N	N	N	Y	N	Y	Y	Y	N	N/A	Y	5
Farhat *et al.*[85]	NPC	Y	Y	Y	Y	N	Y	N	N/A	Y	N	N	N	N	N/A	Y	Y	Y	N	Y	N	N/A	Y	5
Farhat *et al.*[86]	NPC	Y	Y	Y	Y	N	Y	N	N/A	Y	N	N	N	N	N/A	Y	Y	Y	N	Y	N	N/A	Y	5
Farrow *et al.*[87]	NPC	Y	Y	Y	Y	Y	Y	Y	N/A	Y	Y	Y	N	N	N	Y	N	Y	Y	U	N	N/A	Y	5
Feng *et al.*[88]	NPC	Y	Y	Y	Y	N	Y	N	N/A	Y	N	N	N	N	N/A	U	U	Y	Y	U	N	N/A	Y	2
Feng *et al.*[89]	NPC	Y	Y	Y	Y	Y	Y	Y	N/A	Y	Y	Y	N	N	N/A	Y	N	Y	N	Y	Y	N/A	Y	5
Feng *et al.*[90]	NPC	Y	Y	Y	Y	N	Y	Y	N/A	Y	N	N	N	N	N/A	N	Y	Y	U	Y	N	N/A	Y	5
Feng *et al.*[91]	NPC	Y	Y	Y	Y	Y	Y	Y	N/A	Y	Y	Y	N	N	N/A	Y	N	Y	Y	Y	N	N/A	Y	5
Friborg *et al.*[92]	NPC	Y	Y	Y	Y	Y	Y	Y	N	N	N	Y	N	N	N/A	Y	Y	Y	Y	Y	Y	Y	Y	5
Friborg *et al.*[93]	NPC	Y	Y	Y	Y	Y	Y	Y	N	Y	Y	Y	N	N	N/A	U	Y	Y	Y	Y	Y	Y	Y	5
Gao *et al.*[94]	NPC	Y	Y	Y	Y	N	Y	Y	N/A	Y	N	N	N	N	N/A	Y	Y	Y	Y	Y	N	N/A	Y	5

Gao *et al.*[95]	NPC	Y	Y	Y	Y	Y	Y	Y	N/A	Y	N	N	N	N	N/A	Y	Y	Y	Y	U	N	N/A	Y	5
Gao *et al.*[96]	NPC	Y	Y	Y	Y	N	Y	N	N/A	N	N	N	N	N	N/A	U	Y	Y	U	U	N	N/A	Y	2
Gao *et al.*[97]	NPC	Y	Y	Y	N	N	Y	Y	N/A	N	N	N	N	N	N/A	U	Y	Y	U	U	N	N/A	Y	5
Ghandri *et al.*[98]	NPC	Y	Y	Y	Y	N	Y	N	N/A	N	N	N	N	N	N/A	Y	Y	Y	Y	U	N	N/A	N	5
Golovleva *et al.*[99]	NPC	Y	Y	Y	Y	N	Y	Y	N/A	Y	N	N	U	U	N/A	U	Y	U	U	U	N	N/A	Y	2
Guo *et al.*[100]	NPC	Y	Y	Y	Y	N	Y	Y	N/A	Y	N	N	N	N	N/A	U	N	Y	Y	U	Y	N/A	Y	5
Guo *et al.*[101]	NPC	Y	Y	Y	Y	Y	Y	Y	N/A	Y	N	N	N	N	N/A	Y	N	Y	Y	Y	N	N/A	Y	5
Guo *et al.*[102]	NPC	Y	Y	Y	Y	Y	Y	Y	N/A	Y	Y	Y	N	N	N/A	Y	N	Y	N	N	N	N/A	N	5
Guo *et al.*[103]	NPC	Y	Y	Y	Y	Y	Y	Y	N/A	Y	N	N	N	N	N/A	Y	N	Y	Y	Y	N	N/A	Y	5
Guo *et al.*[104]	NPC	Y	Y	Y	Y	Y	Y	Y	N/A	Y	N	N	N	N	N/A	Y	N	Y	Y	Y	Y	N/A	Y	5
Hadhri-Guiga *et al.*[105]	NPC	Y	Y	Y	N	N	Y	N	N/A	Y	N	N	N	N	N/A	U	Y	Y	U	U	N	N/A	N	4
Hall *et al.*[106]	NPC	Y	Y	Y	N	Y	Y	N	N/A	Y	N	N	N	N	N/A	U	Y	Y	U	U	N	N/A	N	1
Hashim *et al.*[107]	NPC	Y	Y	Y	N	Y	Y	Y	N/A	Y	Y	Y	N	N	N/A	U	N	Y	Y	U	N	N/A	Y	4
Hassen *et al.*[108]	NPC	Y	Y	Y	Y	N	Y	Y	N/A	Y	N	N	N	N	N/A	Y	Y	Y	Y	U	N	N/A	Y	5
Hassen *et al.*[109]	NPC	Y	Y	Y	Y	N	Y	N	N/A	N	N	N	N	N	N/A	Y	N	Y	Y	U	N	N/A	Y	5
Hawkins *et al.*[110]	NPC	Y	Y	Y	Y	N	Y	N	N/A	N	N	N	N	N	N/A	U	Y	Y	N	U	N	N/A	Y	4
He *et al.*[111]	NPC	Y	Y	Y	Y	Y	Y	Y	N/A	Y	N	N	N	N	N/A	Y	Y	Y	Y	Y	N	N/A	Y	5
He *et al.*[112]	NPC	Y	Y	Y	Y	N	Y	Y	N/A	Y	N	N	N	N	N/A	Y	N	Y	Y	Y	Y	N/A	N	5
He *et al.*[113]	NPC	Y	Y	Y	Y	Y	N	Y	N/A	Y	N	N	N	N	N/A	Y	Y	Y	Y	Y	N	N/A	N	5
Henderson *et al.*[114]	NPC	Y	Y	Y	Y	Y	Y	N	N/A	Y	Y	Y	N	N	N/A	Y	N	Y	Y	U	N	N/A	Y	5
Hildesheim *et al.*[115]	NPC	Y	Y	Y	Y	N	Y	Y	N/A	N	Y	Y	N	N	N/A	Y	Y	Y	Y	U	Y	N/A	Y	5
Hildesheim *et al.*[116]	NPC	Y	Y	Y	Y	Y	Y	Y	N/A	Y	N	N	N	N	N/A	Y	N	Y	Y	U	N	N/A	N	1
Hildesheim *et al.*[117]	NPC	Y	Y	Y	Y	Y	Y	Y	N/A	Y	N	N	N	N	N/A	Y	N	Y	Y	U	N	N/A	Y	5
Hildesheim *et al.*[118]	NPC	Y	Y	Y	Y	Y	Y	Y	N/A	Y	Y	Y	N	N	N/A	Y	N	Y	Y	U	Y	N/A	Y	5
Hildesheim *et al.*[119]	NPC	Y	Y	Y	Y	Y	Y	Y	N/A	Y	N	N	N	N	N/A	Y	N	Y	Y	U	Y	N/A	Y	5
Hildesheim *et al.*[120]	NPC	Y	Y	Y	N	N	Y	Y	N/A	N	Y	Y	N	N	N/A	U	Y	Y	U	U	N	N/A	Y	4
Hirankarn *et al.*[121]	NPC	Y	Y	Y	Y	N	Y	Y	N/A	Y	N	N	N	N	N/A	N	N	Y	N	U	N	N/A	Y	4
Hirunsatit *et al.*[122]	NPC	Y	Y	Y	Y	N	Y	Y	N/A	N	N	N	N	N	N/A	U	Y	Y	Y	U	Y	N/A	N	5
Ho *et al.*[123]	NPC	Y	Y	Y	N	Y	Y	Y	N/A	Y	N	N	N	N	N/A	Y	Y	Y	U	Y	N	N/A	Y	4
Hsu *et al.*[124]	NPC	Y	Y	Y	Y	Y	Y	Y	N	N	N	N	N	N	N/A	Y	Y	Y	Y	Y	N	Y	Y	5
Hsu *et al.*[125]	NPC	Y	Y	Y	Y	Y	Y	Y	N	N	Y	Y	N	N	N/A	Y	N	Y	Y	Y	Y	Y	Y	5
Hsu *et al.*[126]	NPC	Y	Y	Y	Y	Y	Y	Y	N/A	N	Y	Y	N	N	N/A	U	N	Y	Y	U	Y	N/A	Y	5
Hsu *et al.*[127]	NPC	Y	Y	Y	Y	N	Y	Y	N/A	N	Y	Y	N	N	N/A	Y	N	Y	Y	U	Y	N/A	Y	5
Hsu *et al.*[128]	NPC	Y	Y	Y	Y	N	Y	Y	N/A	N	Y	Y	N	N	N/A	U	Y	Y	U	U	N	N/A	Y	5
Hu *et al.*[129]	NPC	Y	Y	Y	Y	Y	Y	Y	N/A	Y	N	N	N	N	N/A	Y	N	Y	Y	Y	Y	N/A	Y	5
Huang *et al.*[130]	NPC	Y	Y	Y	Y	N	Y	Y	N/A	Y	N	N	N	N	N/A	Y	Y	Y	Y	Y	N	N/A	Y	5
Hung *et al.*[131]	NPC	Y	Y	Y	Y	N	Y	Y	N/A	Y	N	N	N	N	N/A	U	Y	Y	Y	Y	N	N/A	Y	5
Jalbout *et al.*[132]	NPC	Y	Y	Y	Y	N	Y	Y	N/A	Y	N	N	N	N	N/A	N	Y	Y	Y	U	N	N/A	Y	5
Jeannel *et al.*[133]	NPC	Y	Y	Y	Y	N	Y	Y	N/A	Y	Y	Y	N	N	N/A	U	Y	Y	Y	U	N	N/A	Y	3
Ji *et al.*[134]	NPC	Y	Y	Y	N	Y	Y	Y	N/A	Y	Y	Y	N	N	N/A	Y	N	Y	U	U	Y	N/A	Y	5

Jia *et al.*[135]	NPC	Y	Y	Y	N	N	Y	Y	N/A	N	Y	Y	N	N	N/A	Y	N	Y	N	Y	N	N/A	Y	U
Jia *et al.*[136]	NPC	Y	Y	Y	Y	Y	Y	Y	N/A	Y	N	N	N	N	N/A	Y	N	Y	Y	Y	Y	N/A	N	5
Jia *et al.*[137]	NPC	Y	Y	Y	Y	Y	Y	Y	N/A	Y	Y	Y	N	N	N/A	Y	Y	Y	Y	U	Y	N/A	Y	5
Jiang *et al.*[138]	NPC	Y	Y	Y	Y	N	Y	Y	N/A	N	N	N	N	N	N/A	Y	Y	Y	Y	Y	N	N/A	Y	5
Jing *et al.*[139]	NPC	Y	Y	Y	Y	Y	Y	N	N/A	Y	N	N	N	N	N/A	Y	N	Y	Y	U	N	N/A	Y	3
Ko *et al.*[140]	NPC	Y	Y	Y	N	N	Y	Y	N/A	Y	N	N	N	N	N/A	Y	N	Y	U	U	N	N/A	Y	5
Kongruttanachok *et al.*[141]	NPC	Y	Y	Y	Y	N	Y	Y	N/A	N	N	N	N	N	N/A	U	Y	Y	U	U	N	N/A	N	5
Laantri *et al.*[142]	NPC	Y	Y	Y	N	Y	Y	Y	N/A	Y	N	N	N	N	N/A	Y	Y	Y	Y	Y	N	N/A	Y	5
Lakhanpal *et al.*[143]	NPC	Y	Y	Y	Y	N	Y	N	N/A	Y	Y	Y	N	N	N/A	Y	Y	Y	Y	Y	N	N/A	N	4
Lanier *et al.*[144]	NPC	Y	Y	Y	N	N	Y	N	N/A	N	N	N	N	N	N/A	Y	N	Y	U	U	N	N/A	Y	0
Lanier *et al.*[145]	NPC	Y	Y	Y	Y	N	Y	N	N/A	N	N	N	N	N	N/A	N	N	Y	Y	N	N	N/A	Y	0
Laouamri *et al.*[146]	NPC	Y	Y	Y	Y	N	Y	Y	N/A	Y	N	N	N	N	N/A	U	Y	Y	Y	U	N	N/A	Y	3
Laouamri *et al.*[147]	NPC	Y	Y	Y	Y	N	Y	Y	N/A	N	Y	Y	N	N	N/A	U	N	Y	Y	U	N	N/A	Y	3
Lee *et al.*[148]	NPC	Y	Y	Y	Y	Y	Y	Y	N/A	Y	Y	Y	U	U	N/A	U	Y	U	Y	U	Y	N/A	Y	5
Levine *et al.*[149]	NPC	Y	Y	Y	Y	N	Y	Y	N/A	N	Y	Y	N	N	N/A	U	Y	Y	Y	U	Y	N/A	N	4
Li *et al.*[150]	NPC	Y	Y	Y	N	N	Y	N	N/A	Y	N	N	N	N	N/A	U	Y	Y	U	U	N	N/A	Y	1
Li *et al.*[151]	NPC	Y	Y	Y	Y	N	Y	Y	N/A	Y	N	N	U	U	N/A	N	Y	U	Y	Y	N	N/A	Y	3
Li *et al.*[152]	NPC	Y	Y	Y	Y	N	Y	Y	N/A	Y	N	N	N	N	N/A	Y	N	Y	Y	U	N	N/A	Y	5
Li *et al.*[153]	NPC	Y	Y	Y	N	N	Y	Y	N/A	Y	N	N	N	N	N/A	Y	N	Y	U	U	N	N/A	Y	5
Li *et al.*[154]	NPC	Y	Y	Y	N	Y	Y	Y	N/A	Y	N	N	N	N	N/A	Y	N	Y	Y	U	Y	N/A	Y	5
Lin *et al.*[155]	NPC	Y	Y	Y	Y	Y	N	N	N/A	N	Y	Y	N	N	N/A	Y	N	Y	Y	U	N	N/A	N	5
Lin *et al.*[156]	NPC	Y	Y	Y	Y	Y	Y	Y	N/A	N	Y	Y	N	N	N/A	Y	N	Y	Y	U	N	N/A	Y	5
Lin *et al.*[157]	NPC	Y	N	Y	Y	Y	Y	N	N/A	N	U	U	N	N	N/A	Y	N	Y	Y	U	N	N/A	N	5
Lin *et al.*[158]	NPC	Y	Y	Y	Y	Y	Y	Y	N	N	Y	Y	N	N	N/A	N	Y	Y	Y	Y	N	Y	Y	5
Liu *et al.*[159]	NPC	Y	Y	Y	Y	N	Y	Y	N	N	N	N	N	N	N/A	Y	Y	Y	Y	Y	N	Y	Y	5
Liu *et al.*[160]	NPC	Y	Y	Y	Y	N	Y	Y	N/A	Y	N	N	N	N	N/A	Y	N	Y	Y	Y	N	N/A	Y	5
Liu *et al.*[161]	NPC	Y	Y	Y	Y	Y	Y	Y	N/A	Y	N	N	N	N	N/A	Y	Y	Y	N	Y	Y	N/A	Y	5
Liu *et al.*[162]	NPC	Y	Y	Y	Y	N	Y	Y	N/A	Y	Y	Y	N	N	N/A	Y	Y	Y	Y	Y	N	N/A	Y	5
Liu *et al.*[163]	NPC	Y	Y	Y	Y	N	Y	Y	N	Y	N	N	U	U	N/A	U	Y	U	Y	Y	N	N/A	Y	5
Liu *et al.*[164]	NPC	Y	Y	Y	Y	N	Y	Y	N/A	Y	Y	Y	N	N	N/A	Y	Y	Y	Y	Y	N	N/A	Y	5
Lo *et al.*[165]	NPC	Y	Y	Y	Y	Y	Y	Y	N/A	Y	Y	Y	N	N	N/A	Y	Y	Y	Y	U	N	N/A	Y	5
Lourembam *et al.*[166]	NPC	Y	Y	Y	N	N	Y	N	N/A	Y	Y	Y	N	N	N/A	N	Y	Y	U	U	N	N/A	Y	4
Lu *et al.*[167]	NPC	Y	Y	Y	N	N	Y	Y	N/A	N	N	N	N	N	N/A	N	Y	Y	N	U	N	N/A	Y	2
Mabuchi *et al.*[168]	NPC	Y	Y	Y	Y	N	Y	Y	N/A	Y	Y	Y	N	N	N/A	U	Y	Y	Y	U	N	N/A	N	0
Mazzoni *et al.*[169]	NPC	Y	Y	Y	Y	N	Y	N	N/A	N	N	N	N	N	N/A	U	Y	Y	U	U	N	N/A	Y	2
Mirabelli *et al.*[170]	NPC	Y	Y	Y	N	Y	Y	Y	N/A	Y	Y	Y	N	N	N/A	U	N	Y	Y	U	Y	N/A	Y	4
Mo *et al.*[171]	NPC	Y	Y	Y	N	N	Y	N	N/A	N	N	N	N	N	N/A	Y	N	Y	U	U	N	N/A	Y	2
Mokni-Baizig *et al.*[172]	NPC	Y	Y	Y	Y	N	Y	N	N/A	N	N	N	N	N	N/A	Y	N	Y	Y	U	N	N/A	Y	1
Moumad *et al.*[173]	NPC	Y	Y	Y	Y	Y	Y	Y	N/A	Y	N	N	N	N	N/A	Y	N	Y	Y	Y	Y	N/A	Y	5

Mousavi *et al.*[174]	NPC	Y	Y	Y	N	N	Y	Y	N/A	N	N	N	N	N	N/A	Y	Y	Y	Y	Y	Y	N/A	Y	U
Nam *et al.*[175]	NPC	Y	Y	Y	Y	N	Y	Y	N/A	Y	Y	Y	U	U	N/A	U	N	U	Y	U	N	N/A	Y	5
Nazar-Stewart *et al.*[176]	NPC	Y	Y	Y	Y	Y	Y	Y	N/A	N	N	N	N	N	N/A	Y	N	Y	Y	U	N	N/A	Y	3
Nesic *et al.*[177]	NPC	Y	Y	Y	Y	N	Y	Y	N/A	Y	Y	Y	N	N	N/A	U	Y	Y	Y	Y	N	N/A	Y	1
Ng *et al.*[178]	NPC	Y	Y	Y	N	Y	Y	Y	N/A	Y	N	N	U	U	N/A	U	Y	U	U	U	N	N/A	Y	5
Ning *et al.*[179]	NPC	Y	Y	Y	Y	Y	Y	Y	N/A	Y	Y	Y	N	N	N/A	Y	Y	Y	Y	Y	N	N/A	N	4
Nong *et al.*[180]	NPC	Y	Y	Y	Y	N	Y	Y	N/A	Y	N	N	N	N	N/A	Y	Y	Y	N	Y	N	N/A	Y	5
Ooi *et al.*[181]	NPC	Y	Y	Y	N	N	Y	N	N/A	N	N	N	N	N	N/A	U	Y	Y	U	U	N	N/A	Y	2
Ou *et al.*[182]	NPC	Y	Y	Y	N	N	Y	N	N/A	Y	N	N	N	N	N/A	U	Y	Y	N	U	N	N/A	Y	1
Pasini *et al.*[183]	NPC	Y	Y	Y	Y	N	Y	Y	N/A	Y	N	N	N	N	N/A	N	Y	Y	Y	N	N	N/A	Y	3
Polesel *et al.*[184]	NPC	Y	Y	Y	Y	Y	Y	Y	N/A	N	Y	Y	N	N	N/A	Y	Y	Y	Y	Y	Y	N/A	Y	5
Pratesi *et al.*[185]	NPC	Y	Y	Y	N	N	Y	Y	N/A	N	N	N	N	N	N/A	U	Y	Y	U	U	N	N/A	Y	4
Qin *et al.*[186]	NPC	Y	Y	Y	Y	N	Y	Y	N/A	Y	N	N	N	N	N/A	Y	N	Y	Y	U	N	N/A	Y	5
Qiu *et al.*[187]	NPC	Y	Y	Y	Y	N	Y	Y	N/A	Y	N	N	N	N	N/A	Y	Y	Y	Y	U	N	N/A	Y	5
Ren *et al.*[188]	NPC	Y	Y	Y	Y	N	Y	N	N/A	Y	N	N	U	U	N/A	N	Y	U	N	U	N	N/A	Y	3
Ren *et al.*[189]	NPC	Y	Y	Y	Y	Y	Y	Y	N/A	N	Y	Y	N	N	N/A	U	Y	Y	Y	U	Y	N/A	Y	5
Ruan *et al.*[190]	NPC	Y	Y	Y	Y	Y	Y	Y	N/A	Y	Y	Y	N	N	N/A	Y	N	Y	Y	Y	Y	N/A	Y	5
Ruan *et al.*[191]	NPC	Y	Y	Y	Y	Y	Y	Y	N/A	Y	Y	Y	N	N	N/A	Y	Y	Y	N	Y	Y	N/A	Y	5
Shanmugaratnum *et al.*[192]	NPC	Y	Y	Y	Y	N	Y	N	N/A	N	Y	Y	N	N	N/A	U	Y	Y	N	Y	N	N/A	Y	5
Shao *et al.*[193]	NPC	Y	Y	Y	Y	Y	Y	Y	N/A	Y	N	N	N	N	N/A	Y	Y	Y	U	U	Y	N/A	Y	5
Sheng *et al.*[194]	NPC	Y	Y	Y	Y	N	Y	Y	N/A	Y	N	N	N	N	N/A	Y	Y	Y	Y	U	N	N/A	Y	5
Shield *et al.*[195]	NPC	Y	Y	Y	Y	N	Y	N	N/A	N	N	N	N	N	N/A	Y	Y	Y	Y	Y	N	N/A	Y	U
Shih *et al.*[196]	NPC	Y	Y	Y	Y	N	Y	N	N/A	Y	N	N	N	N	N/A	Y	N	Y	Y	Y	N	N/A	Y	5
Simons *et al.*[197]	NPC	Y	Y	Y	Y	N	N	N	N/A	Y	N	N	N	N	N/A	Y	N	Y	U	U	N	N/A	Y	5
Simons *et al.*[198]	NPC	Y	Y	Y	Y	N	Y	N	N/A	Y	N	N	N	N	N/A	Y	Y	Y	U	U	N	N/A	Y	5
Simons *et al.*[199]	NPC	Y	Y	Y	Y	N	Y	N	N/A	N	N	N	N	N	N/A	U	N	Y	N	U	N	N/A	N	0
Simons *et al.*[200]	NPC	N	N	Y	Y	N	Y	N	N/A	N	N	N	N	N	N/A	Y	Y	Y	U	U	N	N/A	Y	5
Song *et al.*[201]	NPC	Y	Y	Y	Y	Y	Y	Y	N/A	N	N	N	N	N	N/A	Y	N	Y	Y	Y	Y	N/A	N	5
Sousa *et al.*[202]	NPC	Y	Y	Y	Y	Y	Y	Y	N/A	Y	N	N	N	N	N/A	Y	Y	Y	Y	U	N	N/A	N	4
Sousa *et al.*[203]	NPC	Y	Y	Y	Y	Y	Y	Y	N/A	Y	N	N	N	N	N/A	Y	Y	Y	Y	U	N	N/A	Y	5
Sousa *et al.[204]*	NPC	Y	Y	Y	Y	Y	Y	Y	N/A	Y	N	N	N	N	N/A	Y	Y	Y	Y	U	U	N/A	N	5
Sousa *et al.*[205]	NPC	Y	Y	Y	Y	Y	Y	Y	N/A	Y	N	N	N	N	N/A	Y	Y	Y	Y	U	Y	N/A	Y	4
Sousa *et al.*[206]	NPC	Y	Y	Y	Y	Y	Y	Y	N/A	Y	N	N	N	N	N/A	Y	Y	Y	Y	U	N	N/A	Y	5
Sousa *et al.*[207]	NPC	Y	Y	Y	Y	Y	Y	Y	N/A	Y	N	N	N	N	N/A	Y	Y	Y	Y	U	Y	N/A	Y	4
Sriamporn *et al.*[208]	NPC	Y	Y	Y	Y	Y	Y	Y	N/A	N	Y	Y	N	N	N/A	U	Y	Y	Y	U	N	N/A	N	4
Su *et al.*[209]	NPC	Y	Y	Y	N	N	Y	N	N/A	N	N	N	N	N	N/A	U	U	Y	U	U	U	N/A	N	5
Sui *et al.*[210]	NPC	Y	Y	Y	Y	Y	Y	Y	N/A	N	N	N	N	N	N/A	Y	N	Y	Y	Y	Y	N/A	Y	5
Tai *et al.*[211]	NPC	Y	Y	Y	N	N	Y	N	N/A	Y	N	N	N	N	N/A	U	Y	Y	Y	U	N	N/A	Y	4

Tang *et al.*[212]	NPC	Y	Y	Y	N	Y	Y	Y	N/A	Y	N	N	U	U	N/A	Y	N	U	Y	U	N	N/A	Y	5
Tang *et al.*[213]	NPC	Y	Y	Y	N	N	Y	Y	N/A	Y	N	N	N	N	N/A	Y	N	Y	U	Y	Y	N/A	Y	5
Tian *et al.*[214]	NPC	Y	Y	Y	N	Y	N	Y	N/A	Y	N	N	N	N	N/A	Y	Y	Y	U	U	N	N/A	Y	5
Tiwawech *et al.*[215]	NPC	Y	Y	Y	Y	Y	Y	Y	N/A	N	N	N	N	N	N	Y	N	Y	Y	U	N	N/A	N	4
Tiwawech *et al.*[216]	NPC	Y	Y	Y	Y	N	Y	Y	N/A	N	N	N	N	N	N/A	Y	N	Y	Y	U	N	N/A	N	3
Tsai *et al.*[217]	NPC	Y	Y	Y	Y	N	Y	Y	N/A	Y	N	N	N	N	N/A	Y	N	Y	Y	Y	N	N/A	N	5
Tse *et al.*[218]	NPC	Y	Y	Y	Y	N	Y	Y	N/A	Y	N	N	N	N	N/A	U	Y	Y	Y	N	Y	N/A	Y	5
Tsou *et al.*[219]	NPC	Y	Y	Y	Y	N	Y	N	N/A	Y	N	N	N	N	N/A	Y	N	Y	Y	Y	N	N/A	N	5
Turkoz *et al.*[220]	NPC	Y	Y	Y	Y	N	Y	Y	N/A	Y	Y	Y	N	N	N/A	Y	U	Y	Y	U	N	N/A	Y	5
Ung *et al.*[221]	NPC	Y	Y	Y	Y	Y	Y	Y	N/A	N	Y	Y	N	N	N/A	U	N	Y	N	U	N	N/A	Y	5
Vaughan *et al.*[222]	NPC	Y	Y	Y	Y	Y	Y	Y	N/A	Y	Y	Y	N	N	N/A	Y	Y	Y	Y	U	N	N/A	Y	5
Vaughan *et al.*[223]	NPC	Y	Y	Y	Y	Y	Y	Y	N/A	Y	Y	Y	N	N	N/A	Y	N	Y	Y	U	Y	N/A	N	5
Wang *et al.*[224]	NPC	Y	Y	Y	Y	Y	Y	Y	N/A	Y	N	N	N	N	N/A	Y	N	Y	Y	U	Y	N/A	Y	5
Ward *et al.*[225]	NPC	Y	Y	Y	Y	Y	Y	Y	N/A	N	Y	Y	N	N	N/A	U	N	Y	Y	U	Y	N/A	N	5
Wei *et al.*[226]	NPC	Y	Y	Y	Y	N	Y	Y	N/A	Y	N	N	N	N	N/A	Y	Y	Y	Y	Y	N	N/A	Y	5
Wei *et al.*[227]	NPC	Y	Y	Y	Y	N	Y	Y	N/A	Y	N	N	N	N	N/A	Y	Y	Y	Y	Y	N	N/A	Y	5
Wei *et al.*[228]	NPC	Y	Y	Y	Y	N	Y	Y	N/A	Y	N	N	N	N	N/A	Y	Y	Y	Y	Y	N	N/A	Y	4
Wei *et al.*[229]	NPC	Y	Y	Y	Y	N	Y	Y	N/A	Y	N	N	N	N	N/A	Y	N	Y	Y	Y	N	N/A	Y	5
Wei *et al.*[230]	NPC	Y	Y	Y	Y	N	Y	N	N/A	N	N	N	N	N	N/A	Y	N	Y	Y	Y	N	N/A	N	5
Wei *et al.*[231]	NPC	Y	Y	Y	Y	N	Y	Y	N/A	Y	N	N	N	N	N/A	Y	N	Y	Y	U	N	N/A	Y	5
West *et al.*[232]	NPC	Y	Y	Y	Y	N	Y	Y	N/A	N	Y	Y	N	N	N/A	Y	Y	Y	Y	U	N	N/A	N	4
Wu *et al.*[233]	NPC	Y	Y	Y	Y	N	Y	Y	N/A	Y	N	N	N	N	N/A	U	N	Y	Y	U	N	N/A	Y	0
Xiao *et al.*[234]	NPC	Y	Y	Y	Y	Y	Y	Y	N/A	Y	N	N	U	U	N/A	Y	Y	U	U	U	Y	N/A	Y	5
Xiao *et al.*[235]	NPC	Y	Y	Y	Y	Y	Y	Y	N/A	Y	N	N	N	N	N/A	Y	Y	Y	U	U	Y	N/A	Y	5
Xiao *et al.*[236]	NPC	Y	Y	Y	Y	Y	Y	Y	N/A	Y	N	N	N	N	N/A	Y	Y	Y	Y	Y	Y	N/A	Y	5
Xie *et al.*[237]	NPC	Y	Y	Y	Y	N	Y	Y	N/A	Y	Y	Y	N	N	N/A	Y	N	Y	Y	Y	N	N/A	Y	5
Xiong *et al.*[238]	NPC	Y	Y	Y	Y	N	Y	N	N/A	Y	N	N	N	N	N/A	U	Y	Y	Y	U	N	N/A	Y	1
Xu *et al.*[239]	NPC	Y	Y	Y	Y	Y	Y	Y	N/A	Y	N	N	N	N	N/A	Y	N	Y	Y	Y	Y	N/A	Y	5
Xu *et al.*[240]	NPC	Y	Y	Y	Y	N	Y	Y	N/A	Y	Y	Y	N	N	N/A	U	N	Y	Y	Y	N	N/A	Y	5
Yang *et al.*[241]	NPC	Y	Y	Y	Y	N	Y	Y	N/A	N	Y	Y	N	N	N/A	N	Y	Y	Y	Y	N	N/A	Y	5
Yang *et al.*[242]	NPC	Y	Y	Y	Y	Y	Y	Y	N/A	N	Y	Y	N	N	N/A	Y	N	Y	Y	U	N	N/A	Y	5
Yang *et al.*[243]	NPC	Y	Y	Y	Y	Y	Y	Y	N/A	Y	N	N	N	N	N/A	Y	N	Y	U	U	N	N/A	Y	5
Yang *et al.*[244]	NPC	Y	Y	Y	Y	Y	Y	Y	N/A	Y	N	N	N	N	N/A	Y	N	Y	U	U	Y	N/A	Y	5
Yang *et al.*[245]	NPC	Y	Y	Y	Y	Y	Y	Y	N/A	Y	N	N	N	N	N/A	Y	Y	Y	U	Y	N	N/A	Y	5
Yang *et al.*[246]	NPC	Y	Y	Y	Y	Y	Y	Y	N/A	Y	N	N	N	N	N/A	Y	Y	Y	N	Y	Y	N/A	Y	5
Ye *et al.*[247]	NPC	Y	Y	Y	Y	N	Y	Y	N/A	Y	N	N	N	N	N/A	U	N	Y	N	N	N	N/A	Y	5
Yew *et al.*[248]	NPC	Y	Y	Y	Y	Y	Y	Y	N/A	Y	N	N	N	N	N/A	N	Y	Y	N	U	N	N/A	Y	5
Yu *et al.*[249]	NPC	Y	Y	Y	Y	Y	Y	N	N/A	N	Y	Y	N	N	N/A	Y	Y	Y	Y	U	U	N/A	Y	5
Yu *et al.*[250]	NPC	Y	Y	Y	Y	Y	Y	Y	N/A	Y	Y	Y	N	N	N/A	Y	Y	Y	Y	Y	Y	N/A	Y	5
Yu *et al.*[251]	NPC	Y	Y	Y	Y	N	Y	Y	N/A	N	Y	Y	N	N	N/A	U	Y	Y	Y	Y	N	N/A	N	5

Yu *et al.*[252]	NPC	Y	Y	Y	Y	N	Y	Y	N/A	Y	Y	Y	N	N	N/A	U	Y	Y	Y	Y	N	N/A	Y	5
Yu *et al.*[253]	NPC	Y	Y	Y	Y	N	Y	Y	N/A	N	N	N	N	N	N/A	Y	Y	Y	Y	U	N	N/A	Y	5
Yuan *et al.*[254]	NPC	Y	Y	Y	Y	N	Y	Y	N/A	Y	Y	Y	N	N	N/A	U	Y	Y	Y	Y	N	N/A	Y	5
Zeng *et al.*[255]	NPC	Y	Y	Y	Y	N	Y	Y	N/A	Y	Y	Y	N	N	N/A	Y	Y	Y	N	U	N	N/A	Y	5
Zhao *et al.*[256]	NPC	Y	Y	Y	Y	Y	Y	Y	N/A	Y	N	N	N	N	N/A	Y	N	Y	Y	U	Y	N/A	Y	5
Zheng *et al.*[257]	NPC	Y	Y	Y	Y	N	Y	Y	N/A	N	Y	Y	N	N	N/A	Y	Y	Y	Y	N	N	N/A	N	4
Zheng *et al.*[258]	NPC	Y	Y	Y	N	Y	Y	Y	N/A	Y	Y	Y	N	N	N/A	Y	Y	Y	Y	U	N	N/A	Y	5
Zheng *et al.*[259]	NPC	Y	Y	Y	N	N	Y	N	N/A	Y	N	N	N	N	N/A	Y	N	Y	U	U	N	N/A	Y	5
Zheng *et al.*[260]	NPC	Y	Y	Y	Y	Y	Y	Y	N/A	Y	N	N	N	N	N/A	Y	Y	Y	Y	U	Y	N/A	Y	5
Zheng *et al.*[261]	NPC	Y	Y	Y	Y	Y	Y	Y	N/A	Y	N	N	N	N	N/A	Y	Y	Y	Y	Y	Y	N/A	Y	5
Zheng *et al.*[262]	NPC	Y	Y	Y	N	N	Y	N	N/A	N	N	N	N	N	N/A	Y	U	Y	U	U	N	N/A	Y	5
Zhou *et al.*[263]	NPC	Y	Y	Y	Y	Y	Y	Y	N/A	Y	N	N	N	N	N/A	Y	Y	Y	Y	Y	N	N/A	Y	5
Zhou *et al.*[264]	NPC	Y	Y	Y	Y	Y	Y	Y	N/A	Y	N	N	N	N	N/A	Y	Y	Y	Y	Y	Y	N/A	Y	5
Zhou *et al.*[265]	NPC	Y	Y	Y	Y	Y	Y	Y	N/A	N	N	N	N	N	N/A	Y	Y	Y	Y	Y	Y	N/A	Y	5
Zhou *et al.*[266]	NPC	Y	Y	Y	Y	Y	Y	Y	N/A	Y	N	N	N	N	N/A	Y	Y	Y	Y	U	N	N/A	Y	5
Zhu *et al.*[267]	NPC	Y	Y	Y	N	N	Y	N	N/A	Y	N	N	U	U	N/A	U	Y	U	U	U	N	N/A	Y	0
Zhu *et al.*[268]	NPC	Y	Y	Y	Y	N	Y	Y	N/A	N	Y	Y	N	N	N/A	U	Y	Y	Y	U	Y	N/A	Y	4
Zhu *et al.*[269]	NPC	Y	Y	Y	Y	N	Y	Y	N/A	Y	N	N	N	N	N/A	Y	Y	Y	Y	U	N	N/A	Y	5
Zhu *et al.*[270]	NPC	Y	Y	Y	Y	Y	Y	Y	N/A	N	N	N	N	N	N/A	Y	Y	Y	Y	U	N	N/A	Y	5
Zong *et al.*[271]	NPC	Y	Y	Y	Y	N	Y	N	N	Y	N	N	N	N	N/A	N	Y	Y	Y	Y	N	N	Y	5

References

1 De Thé G, Geser A, Day NE, Tukei PM, Williams EH, Beri DP, et al. Epidemiological evidence for causal relationship between Epstein-Barr virus and Burkitt's lymphoma from Ugandan prospective study. Nature. 1978;274:756-61.

2 Geser A, De The G, Lenoir G. Final case reporting from the Ugandan prospective study of the relationship between EBV and Burkitt's lymphoma. International Journal of Cancer. 1982;29:397-400.

3 Alexander FE, Jarrett RF, Lawrence D, Armstrong AA, Freeland J, Gokhale DA, et al. Risk factors for Hodgkin's disease by Epstein-Barr virus (EBV) status: prior infection by EBV and other agents. Br J Cancer. 2000;82:1117-21.

4 Alexander FE, Lawrence DJ, Freeland J, Krajewski AS, Angus B, Taylor GM, et al. An epidemiologic study of index and family infectious mononucleosis and adult Hodgkin's disease (HD): evidence for a specific association with EBV+ve HD in young adults. International Journal of Cancer. 2003;107:298-302.

5 Besson C, Roetynck S, Williams F, Orsi L, Amiel C, Lependeven C, et al. Association of killer cell immunoglobulin-like receptor genes with Hodgkin's lymphoma in a familial study. PLoS ONE [Electronic Resource]. 2007;2:e406.

6 Delahaye-Sourdeix M, Urayama KY, Gaborieau V, Veenstra R, Foll M, Chabrier A, et al. A Novel Risk Locus at 6p21.3 for Epstein-Barr Virus-Positive Hodgkin Lymphoma. Cancer Epidemiol Biomarkers Prev. 2015;24:1838-43.

7 Diepstra A, Niens M, Vellenga E, van Imhoff GW, Nolte IM, Schaapveld M, et al. Association with HLA class I in Epstein-Barr-virus-positive and with HLA class III in Epstein-Barr-virus-negative Hodgkin's lymphoma. Lancet. 2005;365:2216-24.

8 Epstein MM, Chang ET, Zhang Y, Fung TT, Batista JL, Ambinder RF, et al. Dietary pattern and risk of hodgkin lymphoma in a population-based case-control study. Am J Epidemiol. 2015;182:405-16.

9 Farrell K, Shield L, Montgomery D, Carman WF, Jarrett R. Seropositivity for human cytomegalovirus is associated with an increased risk of both EBV-negative and EBV-positive classical hodgkin lymphoma. Haematologica. 2010;95:S2.

10 Gao Y, Li Q, Bassig BA, Chang ET, Dai M, Qin Q, et al. Subtype of dietary fat in relation to risk of Hodgkin lymphoma: A population-based case-control study in Connecticut and Massachusetts. Cancer Causes and Control. 2013;24:485-94.

11 Glaser SL, Keegan TH, Clarke CA, Darrow LA, Gomez SL, Dorfman RF, et al. Smoking and Hodgkin lymphoma risk in women United States. Cancer Causes & Control. 2004;15:387-97.

12 Glaser SL, Keegan TH, Clarke CA, Trinh M, Dorfman RF, Mann RB, et al. Exposure to childhood infections and risk of Epstein-Barr virus--defined Hodgkin's lymphoma in women. International Journal of Cancer. 2005;115:599-605.

13 Hjalgrim H, Ekstrom-Smedby K, Rostgaard K, Amini RM, Molin D, Hamilton-Dutoit S, et al. Cigarette smoking and risk of Hodgkin lymphoma: a population-based case-control study. Cancer Epidemiol Biomarkers Prev. 2007;16:1561-6.

14 Hjalgrim H, Smedby KE, Rostgaard K, Molin D, Hamilton-Dutoit S, Chang ET, et al. Infectious mononucleosis, childhood social environment, and risk of Hodgkin lymphoma. Cancer Research. 2007;67:2382-8.

15 Hollander P, Rostgaard K, Smedby KE, Chang ET, Amini RM, de Nully Brown P, et al. Autoimmune and Atopic Disorders and Risk of Classical Hodgkin Lymphoma. Am J Epidemiol. 2015;182:624-32.

16 Jarrett R, Shield L, Lake A, Crae S, Little AM, Taylor GM, et al. Human leukocyte antigen genes are associated with risk of EBV-negative hodgkin lymphoma. Haematologica. 2010;95:S6.

17 Johnson PC, McAulay KA, Montgomery D, Lake A, Shield L, Gallagher A, et al. Modeling HLA associations with EBV-positive and -negative Hodgkin lymphoma suggests distinct mechanisms in disease pathogenesis. Int J Cancer. 2015;137:1066-75.

18 Keegan THM, Glaser SL, Clarke CA, Dorfman RF, Mann RB, DiGiuseppe JA, et al. Body size, physical activity, and risk of Hodgkin's lymphoma in women. Cancer Epidemiology Biomarkers and Prevention. 2006;15:1095-101.
19 Li Q, Chang ET, Bassig BA, Dai M, Qin Q, Gao YS, et al. Body size and risk of Hodgkin's lymphoma by age and gender: a population-based case-control study in Connecticut and Massachusetts. Cancer Causes & Control. 2013;24:287-95.
20 Linabery AM, Erhardt EB, Fonstad RK, Ambinder RF, Bunin GR, Ross JA, et al. Infectious, autoimmune and allergic diseases and risk of Hodgkin lymphoma in children and adolescents: a Children's Oncology Group study. International Journal of Cancer. 2014;135:1454-69.
21 Linabery AM, Erhardt EB, Richardson MR, Ambinder RF, Friedman DL, Glaser SL, et al. Family history of cancer and risk of pediatric and adolescent Hodgkin lymphoma: A Children's Oncology Group study. International Journal of Cancer. 2015;137:2163-74.
22 Niens M, van den Berg A, Diepstra A, Nolte IM, van der Steege G, Gallagher A, et al. The human leukocyte antigen class I region is associated with EBV-positive Hodgkin's lymphoma: HLA-A and HLA complex group 9 are putative candidate genes. Cancer Epidemiology Biomarkers & Prevention. 2006;15:2280-4.
23 Niens M, Jarrett RF, Hepkema B, Nolte IM, Diepstra A, Platteel M, et al. HLA-A 02 is associated with a reduced risk and HLA-A 01 with an increased risk of developing EBV+ Hodgkin lymphoma. Blood. 2007;110:3310-5.
24 Urayama KY, Jarrett RF, Hjalgrim H, Diepstra A, Kamatani Y, Chabrier A, et al. Genome-Wide Association Study of Classical Hodgkin Lymphoma and Epstein-Barr Virus Status-Defined Subgroups. Journal of the National Cancer Institute. 2012;104:240-53.
25 Willett EV, Roman E. Obesity and the risk of Hodgkin lymphoma (United Kingdom). Cancer Causes & Control. 2006;17:1103-6.
26 Willett EV, O'Connor S, Smith AG, Roman E. Does smoking or alcohol modify the risk of Epstein-Barr virus-positive or -negative Hodgkin lymphoma? Epidemiology. 2007;18:130-6.
27 Levine PH, Stemmermann G, Lennette ET, Hildesheim A, Shibata D, Nomura A. Elevated antibody titers to Epstein-Barr virus prior to the diagnosis of Epstein-Barr-virus-associated gastric adenocarcinoma. Int J Cancer. 1995;60:642-4.
28 Armstrong RW, Eng AC. Salted fish and nasopharyngeal carcinoma in Malaysia. Soc Sci Med. 1983;17:1559-67.
29 Armstrong RW, Armstrong MJ, Yu MC, Henderson BE. Salted fish and inhalants as risk factors for nasopharyngeal carcinoma in Malaysian Chinese. Cancer Res. 1983;43:2967-70.
30 Armstrong RW, Imrey PB, Lye MS, Armstrong MJ, Yu MC, Sani S. Nasopharyngeal carcinoma in Malaysian Chinese: salted fish and other dietary exposures. Int J Cancer. 1998;77:228-35.
31 Armstrong RW, Imrey PB, Lye MS, Armstrong MJ, Yu MC, Sani S. Nasopharyngeal carcinoma in Malaysian Chinese: occupational exposures to particles, formaldehyde and heat. Int J Epidemiol. 2000;29:991-8.
32 Bei JX, Li Y, Jia WH, Feng BJ, Zhou GQ, Chen LZ, et al. A genome-wide association study of nasopharyngeal carcinoma identifies three new susceptibility loci. Nature Genetics. 2010;42:599-U173.
33 Ben Hadj Jrad B, Mahfouth W, Bouaouina N, Gabbouj S, Ben Ahmed S, Ltaief M, et al. A polymorphism in FAS gene promoter associated with increased risk of nasopharyngeal carcinoma and correlated with anti-nuclear autoantibodies induction. Cancer Letters. 2006;233:21-7.
34 Ben Chaaben A, Busson M, Douik H, Boukouaci W, Mamoghli T, Chaouch L, et al. Association of IL-12p40 +1188 A/C polymorphism with nasopharyngeal cancer risk and tumor extension. Tissue Antigens. 2011;78:148-51.

35 Ben Chaaben A, Abaza H, Douik H, Chaouch L, Ayari F, Ouni N, et al. Genetic polymorphism of cytochrome P450 2E1 and the risk of nasopharyngeal carcinoma. Bulletin Du Cancer. 2015;102:967-72.

36 Ben Nasr H, Chahed K, Mestiri S, Bouaouina N, Snoussi K, Chouchane L. Association of IL-8 (-251)T/A polymorphism with susceptibility to and aggressiveness of nasopharyngeal carcinoma. Hum Immunol. 2007;68:761-9.

37 Ben Nasr H, Chahed K, Bouaouina N, Chouchane L. Functional vascular endothelial growth factor -2578 C/A polymorphism in relation to nasopharyngeal carcinoma risk and tumor progression. Clin Chim Acta. 2008;395:124-9.

38 Ben Nasr H, Chahed K, Bouaouina N, Chouchane L. PTGS2 (COX-2) -765 G > C functional promoter polymorphism and its association with risk and lymph node metastasis in nasopharyngeal carcinoma. Mol Biol Rep. 2009;36:193-200.

39 Ben Nasr H, Hamrita B, Batbout M, Gabbouj S, Bouaouina N, Chouchane L, et al. A single nucleotide polymorphism in the E-cadherin gene promoter-160 C/A is associated with risk of nasopharyngeal cancer. Clinica Chimica Acta. 2010;411:1253-7.

40 Bendjemana K, Abdennebi M, Gara S, Jmal A, Ghanem A, Touati S, et al. [Genetic polymorphism of gluthation-S transferases and N-acetyl transferases 2 and nasopharyngeal carcinoma: the Tunisia experience]. Bull Cancer. 2006;93:297-302.

41 Birgander R, Sjalander A, Zhou Z, Fan C, Beckman L, Beckman G. p53 polymorphisms and haplotypes in nasopharyngeal cancer. Hum Hered. 1996;46:49-54.

42 Bogger-Goren S, Gotlieb-Stematsky T, Rachima M, Barkowsky E, Schlomo-David J. Nasopharyngeal carcinoma in Israel: epidemiology and Epstein-Barr virus-related serology. European journal of cancer & clinical oncology. 1987;23:1277-81.

43 Bray F, Haugen M, Moger TA, Tretli S, Aalen OO, Grotmol T. Age-incidence curves of nasopharyngeal carcinoma worldwide: Bimodality in low-risk populations and aetiologic implications. Cancer Epidemiology Biomarkers & Prevention. 2008;17:2356-65.

44 Broehm C, Meisner A, Barry M, Wiggins C, Bocklage T. EBV-related nasopharyngeal carcinoma in native americans of New Mexico. Laboratory Investigation. 2017;97:322A.

45 Burt RD, Vaughan TL, McKnight B. Descriptive epidemiology and survival analysis of nasopharyngeal carcinoma in the United States. Int J Cancer. 1992;52:549-56.

46 Burt RD, Vaughan TL, Nisperos B, Swanson M, Berwick M. A protective association between the HLA-A2 antigen and nasopharyngeal carcinoma in US Caucasians. International Journal of Cancer. 1994;56:465-7.

47 Burt RD, Vaughan TL, McKnight B, Davis S, Beckmann AM, Smith AG, et al. Associations between human leukocyte antigen type and nasopharyngeal carcinoma in Caucasians in the United States. Cancer Epidemiology Biomarkers & Prevention. 1996;5:879-87.

48 Butsch Kovacic M, Martin M, Gao X, Fuksenko T, Chen CJ, Cheng YJ, et al. Variation of the killer cell immunoglobulin-like receptors and HLA-C genes in nasopharyngeal carcinoma. Cancer Epidemiol Biomarkers Prev. 2005;14:2673-7.

49 Cao Y, Miao XP, Huang MY, Deng L, Hu LF, Ernberg I, et al. Polymorphisms of XRCC1 genes and risk of nasopharyngeal carcinoma in the Cantonese population. BMC Cancer. 2006;6 (no pagination).

50 Cao Y, Miao XP, Huang MY, Deng L, Liang XM, Lin DX, et al. Polymorphisms of methylenetetrahydrofolate reductase are associated with a high risk of nasopharyngeal carcinoma in a smoking population from Southern China. Mol Carcinog. 2010;49:928-34.

51 Cao SM, Liu ZW, Jia WH, Huang QH, Liu Q, Guo X, et al. Fluctuations of Epstein-Barr Virus Serological Antibodies and Risk for Nasopharyngeal Carcinoma: A Prospective Screening Study with a 20-Year Follow-Up. Plos One. 2011;6.

52 Cao Y, Miao XP, Huang MY, Deng L, Lin DX, Zeng YX, et al. Polymorphisms of death pathway genes FAS and FASL and risk of nasopharyngeal carcinoma. Mol Carcinog. 2010;49:944-50.
53 Chan SH, Day NE, Kunaratnam N, Chia KB, Simons MJ. HLA and nasopharyngeal carcinoma in Chinese--a further study. Int J Cancer. 1983;32:171-6.
54 Chan SH, Chew CT, Prasad U, Wee GB, Srinivasan N, Kunaratnam N. HLA and nasopharyngeal carcinoma in Malays. Br J Cancer. 1985;51:389-92.
55 Chan CK, Mueller N, Evans A, Harris NL, Comstock GW, Jellum E, et al. Epstein-Barr virus antibody patterns preceding the diagnosis of nasopharyngeal carcinoma. Cancer causes & control : CCC. 1991;2:125-31.
56 Chelleng PK, Narain K, Das HK, Chetia M, Mahanta J. Risk factors for cancer nasopharynx: a case-control study from Nagaland, India. Natl Med J India. 2000;13:6-8.
57 Chen CJ, Lian KY, Chang YS, Wang YF, Hsieh T, Hsu MM, et al. Multiple risk factors of nasopharyngeal carcinoma: Epstein-Barr virus, malarial infection, cigarette smoking and familial tendency. Anticancer Research. 1990;10:547-53.
58 Chen DL, Huang TB. A case-control study of risk factors of nasopharyngeal carcinoma. Cancer Lett. 1997;117:17-22.
59 Chen F, Liu K, Huang QH, Liu ZW, Cao SM. [Comparison of incidence of nasopharyngeal carcinoma in populations with different fluctuation modes of immunoglobulin A antibody levels against Epstein-Barr virus capsid antigen]. [Chinese]. Zhonghua yu fang yi xue za zhi [Chinese journal of preventive medicine]. 2012;46:125-8.
60 Cheng YJ, Hildesheim A, Hsu MM, Chen IH, Brinton LA, Levine PH, et al. Cigarette smoking, alcohol consumption and risk of nasopharyngeal carcinoma in Taiwan. Cancer Causes & Control. 1999;10:201-7.
61 Cheng YJ, Chien YC, Hildesheim A, Hsu MM, Chen IH, Chuang J, et al. No association between genetic polymorphisms of CYP1A1, GSTM1, GSTT1, GSTP1, NAT2, and nasopharyngeal carcinoma in Taiwan. Cancer Epidemiol Biomarkers Prev. 2003;12:179-80.
62 Chentoufi AA, AlZahrani KA, Mulia EA, AlMakoshi L, Alshareef R, Paz MR, et al. Association between the frequency of human leukocyte antigen class I and class II genes in Saudi population and relative risk of developing nasopharyngeal carcinoma. Human Immunology. 2016;77:124.
63 Chin YM, Mushiroda T, Takahashi A, Kubo M, Krishnan G, Yap LF, et al. HLA-A SNPs and amino acid variants are associated with nasopharyngeal carcinoma in Malaysian Chinese. International Journal of Cancer. 2015;136:678-87.
64 Cho EY, Hildesheim A, Chen CJ, Hsu MM, Chen IH, Mittl BF, et al. Nasopharyngeal carcinoma and genetic polymorphisms of DNA repair enzymes XRCC1 and hOGG1. Cancer Epidemiology Biomarkers & Prevention. 2003;12:1100-4.
65 Chung SD, Wu CS, Lin HC, Hung SH. Association Between Allergic Rhinitis and Nasopharyngeal Carcinoma: A Population-Based Study. Laryngoscope. 2014;124:1744-9.
66 Coghill AE, Bu W, Nguyen H, Hsu WL, Yu KJ, Lou PJ, et al. Exploring the association between potentially neutralizing antibodies against EBV infection and nasopharyngeal carcinoma. BMC Proceedings Conference: 7th Biannual International Symposium on Nasopharyngeal Carcinoma. 2015;10.
67 Coghill AE, Bu W, Nguyen H, Hsu WL, Yu KJ, Lou PJ, et al. High levels of antibody that neutralize b-cell infection of Epstein-barr virus and that bind ebv gp350 are associated with a lower risk of nasopharyngeal carcinoma. Clinical Cancer Research. 2016;22:3451-7.
68 Cui HY. [Apparent correlation between nasopharyngeal carcinoma and HLA phenotype]. Zhonghua Zhong Liu Za Zhi. 1982;4:249-53.
69 Cui Q, Xu M, Bei JX, Zeng YX. Risk prediction of nasopharyngeal carcinoma by detecting host genetic and Epstein-Barr virus variation in saliva. BMC Proceedings

Conference: 7th Biannual International Symposium on Nasopharyngeal Carcinoma. 2015;10.
70 Da S-J, Liang B, Wu H-L, Chen M-N, Liu P-X. Relationship between GSTM1 gene polymorphism and genetic susceptibility in nasopharyngeal carcinoma. Shi yong ai zheng za zhi= The practical journal of cancer. 2002;17:617-8.
71 Dai Q, Yang ZH, Ye YL. Association between genetic polymorphisms in the DNA repair gene X-ray repair cross-complementing 1-Arg399Gln and the risk of nasopharyngeal carcinoma in Chinese population from Sichuan province. [Chinese]. Journal of Clinical Rehabilitative Tissue Engineering Research. 2007;11:5861-4.
72 Dai W, Zheng H, Cheung AKL, Tang CSM, Ko JMY, Wong BWY, et al. Whole-exome sequencing identifies MST1R as a genetic susceptibility gene in nasopharyngeal carcinoma. Proceedings of the National Academy of Sciences of the United States of America. 2016;113:3317-22.
73 Dardari R, Khyatti M, Jouhadi H, Benider A, Ettayebi H, Kahlain A, et al. Study of human leukocyte antigen class I phenotypes in Moroccan patients with nasopharyngeal carcinoma. Int J Cancer. 2001;92:294-7.
74 Delfitri M. No Association between HLA-DQB1 Genotypes with Nasopharyngeal Carcinoma in Batak Ethnic Groups in Indonesia. Biomedical Research-India. 2011;22:235-40.
75 Deng L, Zhao X-R, Pan KF, Wang Y, Deng X-Y, Lu Y-Y, et al. Cyclin D1 Polymorphism and the Susceptibility to NPC Using DHPLC. Acta Biochimica et Biophysica Sinica. 2002;34:16-20.
76 Deng ZL, Wei YP, Ma Y. [Frequent genetic deletion of detoxifying enzyme GSTM1 and GSTT1 genes in nasopharyngeal carcinoma patients in Guangxi Province, China]. Zhonghua Zhong Liu Za Zhi. 2004;26:598-600.
77 Deng Z, Wei Y, Luo W, Liao Z, Ma Y. Glutathione S-transferase M1 and T1 Gene Deletion Associated with Increased Susceptibility to Nasopharyngeal Carcinoma. The Chinese-German Journal of Clinical Oncology. 2005;4:276-8.
78 Douik H, Ben Chaaben A, Attia Romdhane N, Romdhane HB, Mamoghli T, Fortier C, et al. Association of MICA-129 polymorphism with nasopharyngeal cancer risk in a Tunisian population. Hum Immunol. 2009;70:45-8.
79 Douik H, Romdhane NA, Guemira F. Are HLA-E*0103 alleles predictive markers for nasopharyngeal cancer risk? Pathology Research and Practice. 2016;212:345-9.
80 Ekburanawat W, Ekpanyaskul C, Brennan P, Kanka C, Tepsuwan K, Temiyastith S, et al. Evaluation of non-viral risk factors for nasopharyngeal carcinoma in Thailand: results from a case-control study. Asian Pac J Cancer Prev. 2010;11:929-32.
81 Fachiroh J, Sangrajrang S, Johansson M, Renard H, Gaborieau V, Chabrier A, et al. Tobacco consumption and genetic susceptibility to nasopharyngeal carcinoma (NPC) in Thailand. Cancer Causes Control. 2012;23:1995-2002.
82 Fan Q, Jia WH, Zhang RH, Yu XJ, Chen LZ, Feng QS, et al. Correlation of polymeric immunoglobulin receptor gene polymorphisms to susceptibility of nasopharyngeal carcinoma. [Chinese]. Ai zheng = Aizheng = Chinese journal of cancer. 2005;24:915-8.
83 Fan Q, Jia WH, Zhang RH, Yu XJ, Chen LZ, Feng QS, et al. Association of pIgR polymorphisms with nasopharyngeal carcinoma. Chinese Journal of Cancer Research. 2006;18:168-72.
84 Fan Q, He JF, Wang QR, Cai HB, Sun XG, Zhou XX, et al. Functional polymorphism in the 5'-UTR of CR2 is associated with susceptibility to nasopharyngeal carcinoma. Oncology Reports. 2013;30:11-6.
85 Farhat K, Hassen E, Bouzgarrou N, Gabbouj S, Bouaouina N, Chouchane L. Functional IL-18 promoter gene polymorphisms in Tunisian nasopharyngeal carcinoma patients. Cytokine. 2008;43:132-7.
86 Farhat K, Hassen E, Gabbouj S, Bouaouina N, Chouchane L. Interleukin-10 and interferon-gamma gene polymorphisms in patients with nasopharyngeal carcinoma. International Journal of Immunogenetics. 2008;35:197-205.

87 Farrow DC, Vaughan TL, Berwick M, Lynch CF, Swanson GM, Lyon JL. Diet and nasopharyngeal cancer in a low-risk population. Int J Cancer. 1998;78:675-9.
88 Feng BJ, Huang W, Shugart YY, Lee MK, Zhang F, Xia JC, et al. Genome-wide scan for familial nasopharyngeal carcinoma reveals evidence of linkage to chromosome 4. Nature Genetics. 2002;31:395-9.
89 Feng BJ, Jalbout M, Ayoub WB, Khyatti M, Dahmoul S, Ayad M, et al. Dietary risk factors for nasopharyngeal carcinoma in Maghrebian countries. International Journal of Cancer. 2007;121:1550-5.
90 Feng XL, Zhou W, Li H, Fang WY, Zhou YB, Yao KT, et al. The DLC-1 -29A/T polymorphism is not associated with nasopharyngeal carcinoma risk in Chinese population. Genet Test. 2008;12:345-9.
91 Feng BJ, Khyatti M, Ben-Ayoub W, Dahmoul S, Ayad M, Maachi F, et al. Cannabis, tobacco and domestic fumes intake are associated with nasopharyngeal carcinoma in North Africa. Br J Cancer. 2009;101:1207-12.
92 Friborg J, Wohlfahrt J, Koch A, Storm H, Olsen OR, Melbye M. Cancer susceptibility in nasopharyngeal carcinoma families - A population-based cohort study. Cancer Research. 2005;65:8567-72.
93 Friborg JT, Yuan JM, Wang RW, Koh WP, Lee HP, Yu MC. A prospective study of tobacco and alcohol use as risk factors for pharyngeal carcinomas in Singapore Chinese. Cancer. 2007;109:1183-91.
94 Gao LB, Wei YS, Zhou B, Wang YY, Liang WB, Li C, et al. No association between epidermal growth factor and epidermal growth factor receptor polymorphisms and nasopharyngeal carcinoma. Cancer Genet Cytogenet. 2008;185:69-73.
95 Gao LB, Liang WB, Xue H, Rao L, Pan XM, Lv ML, et al. Genetic polymorphism of interleukin-16 and risk of nasopharyngeal carcinoma. Clin Chim Acta. 2009;409:132-5.
96 Gao SQ, Deng ZH. A study on the correlation of killer immunoglobulin-like receptor gene diversity with nasopharyngeal carcinoma in the southern chinese han population. Vox Sanguinis. 2012;103:265-6.
97 Gao X, Tang M, Martin P, Zeng Y, Carrington M. Effect of HLA and KIR polymorphism on NPC risk. BMC Proceedings Conference: 7th Biannual International Symposium on Nasopharyngeal Carcinoma. 2015;10.
98 Ghandri N, Gabbouj S, Farhat K, Bouaouina N, Abdelaziz H, Nouri A, et al. Association of HLA-G polymorphisms with nasopharyngeal carcinoma risk and clinical outcome. Human Immunology. 2011;72:150-8.
99 Golovleva I, Birgander R, Sjalander A, Lundgren E, Beckman L. Interferon-alpha and p53 alleles involved in nasopharyngeal carcinoma. Carcinogenesis. 1997;18:645-7.
100 Guo XC, O'Brien SJ, Winkler C, Scott K, Hutcheson H, David V, et al. Association study of chromosome 4 STRs polymorphisms with nasopharyngeal carcinoma. [Chinese]. Yi chuan = Hereditas / Zhongguo yi chuan xue hui bian ji. 2006;28:783-90.
101 Guo XC, O'Brien SJ, Zeng Y, Nelson GW, Winkler CA. GSTM1 and GSTT1 gene deletions and the risk for nasopharyngeal carcinoma in Han Chinese. Cancer Epidemiology Biomarkers & Prevention. 2008;17:1760-3.
102 Guo XC, Johnson RC, Deng H, Liao J, Guan L, Nelson GW, et al. Evaluation of nonviral risk factors for nasopharyngeal carcinoma in a high-risk population of Southern China. International Journal of Cancer. 2009;124:2942-7.
103 Guo X, Zeng Y, Deng H, Liao J, Zheng Y, Li J, et al. Genetic Polymorphisms of CYP2E1, GSTP1, NQO1 and MPO and the Risk of Nasopharyngeal Carcinoma in a Han Chinese Population of Southern China. BMC Res Notes. 2010;3:212.
104 Guo X, Winkler CA, Li J, Guan L, Tang M, Liao J, et al. Evaluation and integration of genetic signature for prediction risk of nasopharyngeal carcinoma in Southern China. BioMed Research International. 2014;2014 (no pagination).
105 Hadhri-Guiga B, Toumi N, Khabir A, Sellami-Boudawara T, Ghorbel A, Daoud J, et al. Proline homozygosity in codon 72 of TP53 is a factor of susceptibility to nasopharyngeal carcinoma in Tunisia. Cancer Genet Cytogenet. 2007;178:89-93.

106 Hall PJ, Levin AG, Entwistle CC, Knight SC, Wasunna A, Kung'u A, et al. HLA antigens in East African Black patients with Burkitt's lymphoma or nasopharyngeal carcinoma and in controls: a pilot study. Hum Immunol. 1982;5:91-105.
107 Hashim NAN, Ramzi NH, Velapasamy S, Alex L, Chahil JK, Lye SH, et al. Identification of Genetic and Non-genetic Risk Factors for Nasopharyngeal Carcinoma in a Southeast Asian Population. Asian Pacific Journal of Cancer Prevention. 2012;13:6005-10.
108 Hassen E, Farhat K, Gabbouj S, Jalbout M, Bouaouina N, Chouchane L. TAP1 gene polymorphisms and nasopharyngeal carcinoma risk in a Tunisian population. Cancer Genet Cytogenet. 2007;175:41-6.
109 Hassen E, Ghedira R, Ghandri N, Farhat K, Gabbouj S, Bouaouina N, et al. Lack of Association Between Human Leukocyte Antigen-E Alleles and Nasopharyngeal Carcinoma in Tunisians. DNA and Cell Biology. 2011;30:603-9.
110 Hawkins BR, Simons MJ, Goh EH, Chia KB, Shanmugaratnam K. Immunogenetic aspects of nasopharyngeal carcinoma. II. Analysis of ABO, rhesus and MNSs red cell systems. Int J Cancer. 1974;13:116-21.
111 He Y, Zhou G, Zhai Y, Dong X, Lv L, He F, et al. Association of PLUNC gene polymorphisms with susceptibility to nasopharyngeal carcinoma in a Chinese population. Journal of Medical Genetics. 2005;42:172-6.
112 He JF, Jia WH, Fan Q, Zhou XX, Qin HD, Shugart YY, et al. Genetic polymorphisms of TLR3 are associated with Nasopharyngeal carcinoma risk in Cantonese population. BMC Cancer. 2007;7 (no pagination).
113 He Y, Zhou GQ, Li X, Dong XJ, Chai XQ, Yao KT. Correlation of polymorphism of the coding region of glutathione S- transferase M1 to susceptibility of nasopharyngeal carcinoma in South China population. Ai Zheng. 2009;28:5-7.
114 Henderson BE, Louie E, SooHoo Jing J, Buell P, Gardner MB. Risk factors associated with nasopharyngeal carcinoma. N Engl J Med. 1976;295:1101-6.
115 Hildesheim A, West S, DeVeyra E, De Guzman MF, Jurado A, Jones C, et al. Herbal medicine use, Epstein-Barr virus, and risk of nasopharyngeal carcinoma. Cancer Research. 1992;52:3048-51.
116 Hildesheim A, Chen CJ, Caporaso NE, Cheng YJ, Hoover RN, Hsu MM, et al. Cytochrome P4502E1 genetic polymorphisms and risk of nasopharyngeal carcinoma: results from a case-control study conducted in Taiwan. Cancer Epidemiol Biomarkers Prev. 1995;4:607-10.
117 Hildesheim A, Anderson LM, Chen CJ, Cheng YJ, Brinton LA, Daly AK, et al. CYP2E1 genetic polymorphisms and risk of nasopharyngeal carcinoma in Taiwan. Journal of the National Cancer Institute. 1997;89:1207-12.
118 Hildesheim A, Dosemeci M, Chan CC, Chen CJ, Cheng YJ, Hsu MM, et al. Occupational exposure to wood, formaldehyde, and solvents and risk of nasopharyngeal carcinoma. Cancer Epidemiology Biomarkers and Prevention. 2001;10:1145-53.
119 Hildesheim A, Apple RJ, Chen CJ, Wang SS, Cheng YJ, Klitz W, et al. Association of HLA class I and II alleles and extended haplotypes with nasopharyngeal carcinoma in Taiwan. Journal of the National Cancer Institute. 2002;94:1780-9.
120 Hildesheim A, West S, Jones C, Deveyra E, Hinuma Y. EPSTEIN-BARR-VIRUS, HERBAL MEDICINE USE, AND RISK OF NASOPHARYNGEAL CARCINOMA. American Journal of Epidemiology. 1991;134:758-.
121 Hirankarn N, Kimkong I, Mutirangura A. HLA-E polymorphism in patients with nasopharyngeal carcinoma. Tissue Antigens. 2004;64:588-92.
122 Hirunsatit R, Kongruttanachok N, Shotelersuk K, Supiyaphun P, Voravud N, Sakuntabhai A, et al. Polymeric immunoglobulin receptor polymorphisms and risk of nasopharyngeal cancer. Bmc Genetics. 2003;4.
123 Ho SY, Wang YJ, Huang PC, Tsai ST, Chen CH, Chen HHW, et al. Evaluation of the associations between the single nucleotide polymorphisms of the promoter region of

the tumor necrosis factor-alpha gene and nasopharyngeal carcinoma. Journal of the Chinese Medical Association. 2006;69:351-7.
124 Hsu WL, Chen JY, Chien YC, Liu MY, You SL, Hsu MM, et al. Independent effect of EBV and cigarette smoking on nasopharyngeal carcinoma: A 20-year follow-up study on 9,622 males without family history in Taiwan. Cancer Epidemiology Biomarkers and Prevention. 2009;18:1218-26.
125 Hsu WL, Yu KJ, Chien YC, Chiang CJ, Cheng YJ, Chen JY, et al. Familial tendency and risk of nasopharyngeal carcinoma in Taiwan: Effects of covariates on risk. American Journal of Epidemiology. 2011;173:292-9.
126 Hsu WL, Pan WH, Chien YC, Yu KJ, Cheng YJ, Chen JY, et al. Lowered Risk of Nasopharyngeal Carcinoma and Intake of Plant Vitamin, Fresh Fish, Green Tea and Coffee: A Case-Control Study in Taiwan. Plos One. 2012;7.
127 Hsu WL, Yu KJ, Chien YC, Chen TC, Lin CY, Tsou YA, et al. Familial tendency and environmental co-factors of nasopharyngeal carcinoma: The gene-environment interaction study on nasopharyngeal carcinoma in Taiwan. BMC Proceedings Conference: 7th Biannual International Symposium on Nasopharyngeal Carcinoma. 2015;10.
128 Hsu WL, Chien YC, Yu KJ, Wang CP, Lin CY, Tsou YA, et al. Prediction of nasopharyngeal carcinoma risk by Epstein-Barr virus seromarkers and environmental co-factors: The gene-environment interaction study on nasopharyngeal carcinoma in Taiwan. BMC Proceedings Conference: 7th Biannual International Symposium on Nasopharyngeal Carcinoma. 2015;10.
129 Hu S, Zhou G, Zhang L, Jiang H, Xiao M. The effects of functional polymorphisms in the TGFbeta1 gene on nasopharyngeal carcinoma susceptibility. Otolaryngol Head Neck Surg. 2012;146:579-84.
130 Huang X, Cao Z, Zhang Z, Yang Y, Wang J, Fang D. No association between Vitamin D receptor gene polymorphisms and nasopharyngeal carcinoma in a Chinese Han population. Biosci Trends. 2011;5:99-103.
131 Hung SH, Chen PY, Lin HC, Ting J, Chung SD. Association of Rhinosinusitis With Nasopharyngeal Carcinoma: A Population-Based Study. Laryngoscope. 2014;124:1515-20.
132 Jalbout M, Bouaouina N, Gargouri J, Corbex M, Ben Ahmed S, Chouchane L. Polymorphism of the stress protein HSP70-2 gene is associated with the susceptibility to the nasopharyngeal carcinoma. Cancer Lett. 2003;193:75-81.
133 Jeannel D, Hubert A, de Vathaire F, Ellouz R, Camoun M, Ben Salem M, et al. Diet, living conditions and nasopharyngeal carcinoma in Tunisia--a case-control study. Int J Cancer. 1990;46:421-5.
134 Ji X, Zhang W, Xie C, Wang B, Zhang G, Zhou F. Nasopharyngeal carcinoma risk by histologic type in central China: impact of smoking, alcohol and family history. Int J Cancer. 2011;129:724-32.
135 Jia WH, Feng BJ, Xu ZL, Zhang XS, Huang P, Huang LX, et al. Familial risk and clustering of nasopharyngeal carcinoma in Guangdong, China. Cancer. 2004;101:363-9.
136 Jia WH, Pan QH, Qin HD, Xu YF, Shen GP, Chen L, et al. A Case-control and a family-based association study revealing an association between CYP2E1 polymorphisms and nasopharyngeal carcinoma risk in Cantonese. Carcinogenesis. 2009;30:2031-6.
137 Jia WH, Luo XY, Feng BJ, Ruan HL, Bei JX, Liu WS, et al. Traditional Cantonese diet and nasopharyngeal carcinoma risk: a large-scale case-control study in Guangdong, China. BMC Cancer. 2010;10:446.
138 Jiang Y, Li N, Dong P, Zhang N, Sun Y, Han M, et al. Polymorphisms in GSTM1, GSTTI and GSTP1 and nasopharyngeal cancer in the east of China: A case-control study. Asian Pacific Journal of Cancer Prevention. 2011;12:3097-100.

139 Jing J, Louie E, Henderson BE, Terasaki P. Histocompatibility leukocyte antigen patterns in nasopharyngeal carcinoma cases from California. Natl Cancer Inst Monogr. 1977;47:153-6.
140 Ko JMY, Dai W, Wong EHW, Kwong D, Ng WT, Lee A, et al. Multigene pathway-based analyses identify nasopharyngeal carcinoma risk associations for cumulative adverse effects of TERT-CLPTM1L and DNA double-strand breaks repair. International Journal of Cancer. 2014;135:1634-45.
141 Kongruttanachok N, Sukdikul S, Setavarin S, Kerekhjanarong V, Supiyaphun P, Voravud N, et al. Cytochrome P450 2E1 polymorphism and nasopharyngeal carcinoma development in Thailand: a correlative study. BMC Cancer. 2001;1:4.
142 Laantri N, Jalbout M, Khyatti M, Ayoub WB, Dahmoul S, Ayad M, et al. XRCC1 and hOGG1 genes and risk of nasopharyngeal carcinoma in North African countries. Mol Carcinog. 2011;50:732-7.
143 Lakhanpal M, Singh LC, Rahman T, Sharma J, Singh MM, Kataki AC, et al. Contribution of susceptibility locus at HLA class I region and environmental factors to occurrence of nasopharyngeal cancer in Northeast India. Tumor Biology. 2015;36:3061-73.
144 Lanier A, Bender T, Talbot M, Wilmeth S, Tschopp C, Henle W, et al. Nasopharyngeal carcinoma in Alaskan Eskimos, Indians, and Aleuts: A review of cases and study of Epstein-Barr virus, HLA, and environmental risk factors. Cancer. 1980;46:2100-6.
145 Lanier AP, Henle W, Bender TR, Henle G, Talbot ML. Epstein-Barr virus-specific antibody titers in seven Alaskan natives before and after diagnosis of nasopharyngeal carcinoma. Int J Cancer. 1980;26:133-7.
146 Laouamri S, Hamdi-Cherif M. Nasopharyngeal carcinoma and familial cancer in Setif (Algeria). Himmich H, editor2000.
147 Laouamri S, Hamdi-Cherif M, Sekfali N, Mokhtari L, Kharchi R. Dietary risk factors of nasopharyngeal carcinoma in the Setif area in Algeria. Revue D Epidemiologie Et De Sante Publique. 2001;49:145-56.
148 Lee HP, Gourley L, Duffy SW, Esteve J, Lee J, Day NE. Preserved foods and nasopharyngeal carcinoma: a case-control study among Singapore Chinese. Int J Cancer. 1994;59:585-90.
149 Levine R, Zhu K, Gu Y, Brann E, Hall I, Caplan L, et al. Self-reported infectious mononucleosis and 6 cancers: a population-based, case-control study. Scand J Infect Dis. 1998;30:211-4.
150 Li PK, Poon AS, Tsao SY, Ho S, Tam JS, So AK, et al. No association between HLA-DQ and -DR genotypes with nasopharyngeal carcinoma in southern Chinese. Cancer Genet Cytogenet. 1995;81:42-5.
151 Li W, Ray RM, Gao DL, Fitzgibbons ED, Seixas NS, Camp JE, et al. Occupational risk factors for nasopharyngeal cancer among female textile workers in Shanghai, China. Occup Environ Med. 2006;63:39-44.
152 Li X, Ghandri N, Piancatelli D, Adams S, Chen D, Robbins FM, et al. Associations between HLA class I alleles and the prevalence of nasopharyngeal carcinoma (NPC) among Tunisians. J Transl Med. 2007;5:22.
153 Li ZH, Pan XM, Han BW, Han HB, Zhang Z, Gao LB. No association between ACE polymorphism and risk of nasopharyngeal carcinoma. J Renin Angiotensin Aldosterone Syst. 2012;13:210-5.
154 Li LJ, Wu J, Sima XT, Bai P, Deng W, Deng XK, et al. Interactions of miR-34b/c and TP-53 polymorphisms on the risk of nasopharyngeal carcinoma. Tumor Biology. 2013;34:1919-23.
155 Lin TM, Chen KP, Lin CC, Hsu MM, Tu SM, Chiang TC, et al. Retrospective study on nasopharyngeal carcinoma. J Natl Cancer Inst. 1973;51:1403-8.
156 Lin TM, Yang CS, Tu SM, Chen CJ, Kuo KC, Hirayama T. Interaction of factors associated with cancer of the nasopharynx. Cancer. 1979;44:1419-23.

157 Lin TM, Chang HJ, Chen CJ. Risk factors for nasopharyngeal carcinoma. Anticancer Research. 1986;6:791-6.
158 Lin Y-H, Chen C-J. A cohort study on multiple risk factors of nasopharyngeal carcinoma. Chinese Journal of Public Health. 1997;16:466-77.
159 Liu Z, Fang F, Chang ET, Ye W. Cancer risk in the relatives of patients with nasopharyngeal carcinoma - A register-based cohort study in Sweden. Br J Cancer. 2015;112:1827-31.
160 Liu JC, Tsai CW, Hsu CM, Chang WS, Li CY, Liu SP, et al. Contribution of Double Strand Break Repair Gene XRCC3 Genotypes to Nasopharyngeal Carcinoma Risk in Taiwan. Chinese Journal of Physiology. 2015;58:64-71.
161 Liu YH, Qiu FM, Yang L, Yang RR, Yang XR, Huang DS, et al. Polymorphisms of NF kappa B1 and I kappa B alpha and Their Synergistic Effect on Nasopharyngeal Carcinoma Susceptibility. Biomed Research International. 2015.
162 Liu ZW, Fang F, Chang ET, Adami HO, Ye WM. Sibship size, birth order and risk of nasopharyngeal carcinoma and infectious mononucleosis: a nationwide study in Sweden. International Journal of Epidemiology. 2016;45:825-34.
163 Liu ZW, Coghill AE, Pfeiffer RM, Hsu WL, Lou PJ, Wang CP, et al. Birth order and risk of nasopharyngeal carcinoma in multiplex families from Taiwan. International Journal of Cancer. 2016;139:2467-73.
164 Liu ZW, Chang ET, Liu Q, Cai YL, Zhang Z, Chen GM, et al. Oral Hygiene and Risk of Nasopharyngeal Carcinoma-A Population-Based Case-Control Study in China. Cancer Epidemiology Biomarkers & Prevention. 2016;25:1201-7.
165 Lo YL, Pan WH, Hsu WL, Chien YC, Chen JY, Hsu MM, et al. Partial least square discriminant analysis discovered a dietary pattern inversely associated with nasopharyngeal carcinoma risk. PLoS ONE. 2016;11 (6) (no pagination).
166 Lourembam DS, Singh AR, Sharma TD, Singh TS, Singh TR, Singh LS. Evaluation of Risk Factors for Nasopharyngeal Carcinoma in a High-risk Area of India, the Northeastern Region. Asian Pacific journal of cancer prevention : APJCP. 2015;16:4927-35.
167 Lu CC, Chen JC, Jin YT, Yang HB, Chan SH, Tsai ST. Genetic susceptibility to nasopharyngeal carcinoma within the HLA-A locus in Taiwanese. International Journal of Cancer. 2003;103:745-51.
168 Mabuchi K, Bross DS, Kessler, II. Cigarette smoking and nasopharyngeal carcinoma. Cancer. 1985;55:2874-6.
169 Mazzoni E, Martini F, Corallini A, Taronna A, Barbanti-Brodano G, Querzoli P, et al. Serologic investigation of undifferentiated nasopharyngeal carcinoma and simian virus 40 infection. Head and Neck-Journal for the Sciences and Specialties of the Head and Neck. 2016;38:232-6.
170 Mirabelli MC, Hoppin JA, Tolbert PE, Herrick RF, Gnepp DR, Brann EA. Occupational exposure to chlorophenol and the risk of nasal and nasopharyngeal cancers among U.S. men aged 30 to 60. Am J Ind Med. 2000;37:532-41.
171 Mo WN, Tang AZ, Zhou L, Huang GW, Wang Z, Zeng Y. Analysis of Epstein-Barr viral DNA load, EBV-LMP2 specific cytotoxic T-lymphocytes and levels of CD4(+)CD25(+) T cells in patients with nasopharyngeal carcinomas positive for IgA antibody to EBV viral capsid antigen. Chinese Medical Journal. 2009;122:1173-8.
172 Mokni-Baizig N, Ayed K, Ayed FB, Ayed S, Sassi F, Ladgham A, et al. Association between HLA-A/-B antigens and -DRB1 alleles and nasopharyngeal carcinoma in Tunisia. Oncology. 2001;61:55-8.
173 Moumad K, Lascorz J, Bevier M, Khyatti M, Ennaji MM, Benider A, et al. Genetic polymorphisms in host innate immune sensor genes and the risk of nasopharyngeal carcinoma in North Africa. G3: Genes, Genomes, Genetics. 2013;3:971-7.
174 Mousavi SM, Sundquist J, Hemminki K. Nasopharyngeal and hypopharyngeal carcinoma risk among immigrants in Sweden. International Journal of Cancer. 2010;127:2888-92.

175 Nam JM, McLaughlin JK, Blot WJ. Cigarette smoking, alcohol, and nasopharyngeal carcinoma: a case-control study among U.S. whites. J Natl Cancer Inst. 1992;84:619-22.
176 Nazar-Stewart V, Vaughan TL, Burt RD, Chen C, Berwick M, Swanson GM. Glutathione S-transferase M1 and susceptibility to nasopharyngeal carcinoma. Cancer Epidemiol Biomarkers Prev. 1999;8:547-51.
177 Nesic V, Sipetic S, Vlajinac H, Stosic-Divjak S, Jesic S. Risk Factors for the Occurrence of Undifferentiated Carcinoma of Nasopharyngeal Type: A Case-Control Study. Srpski Arhiv Za Celokupno Lekarstvo. 2010;138:6-10.
178 Ng CC, Yew PY, Puah SM, Krishnan G, Yap LF, Teo SH, et al. A genome-wide association study identifies ITGA9 conferring risk of nasopharyngeal carcinoma. J Hum Genet. 2009;54:392-7.
179 Ning JP, Yu MC, Wang QS, Henderson BE. Consumption of salted fish and other risk factors for nasopharyngeal carcinoma (NPC) in Tianjin, a low-risk region for NPC in the People's Republic of China. J Natl Cancer Inst. 1990;82:291-6.
180 Nong LG, Luo B, Zhang L, Nong HB. Interleukin-18 gene promoter polymorphism and the risk of nasopharyngeal carcinoma in a Chinese population. DNA and Cell Biology. 2009;28:507-13.
181 Ooi EE, Ren EC, Chan SH. Association between microsatellites within the human MHC and nasopharyngeal carcinoma. Int J Cancer. 1997;74:229-32.
182 Ou B-X, H-Y. R, Fan Y, Liu L-M. Study on association between HLA-A, B, C, DR and nasopharyngeal cancer (NPC) in Guangzhou area. Ai zheng = Aizheng = Chinese journal of cancer. 1985;4:5-7.
183 Pasini E, Caggiari L, Dal Maso L, Martorelli D, Guidoboni M, Vaccher E, et al. Undifferentiated nasopharyngeal carcinoma from a nonendemic area: Protective role of HLA allele products presenting conserved EBV epitopes. International Journal of Cancer. 2009;125:1358-64.
184 Polesel J, Franceschi S, Talamini R, Negri E, Barzan L, Montella M, et al. Tobacco smoking, alcohol drinking, and the risk of different histological types of nasopharyngeal cancer in a low-risk population. Oral Oncology. 2011;47:541-5.
185 Pratesi C, Bortolin MT, Bidoli E, Tedeschi R, Vaccher E, Dolcetti R, et al. Interleukin-10 and interleukin-18 promoter polymorphisms in an Italian cohort of patients with undifferentiated carcinoma of nasopharyngeal type. Cancer Immunology Immunotherapy. 2006;55:23-30.
186 Qin HD, Shugart YY, Bei JX, Pan QH, Chen LN, Feng QS, et al. Comprehensive Pathway-Based Association Study of DNA Repair Gene Variants and the Risk of Nasopharyngeal Carcinoma. Cancer Research. 2011;71:3000-8.
187 Qiu F, Yang L, Zhang L, Yang X, Yang R, Fang W, et al. Polymorphism in mature microRNA-608 sequence is associated with an increased risk of nasopharyngeal carcinoma. Gene. 2015;565:180-6.
188 Ren W, Zheng H, Li M, Deng L, Li XL, Pan KF, et al. A functional single nucleotide polymorphism site detected in nasopharyngeal carcinoma-associated transforming gene Tx. Cancer Genet Cytogenet. 2005;157:49-52.
189 Ren ZF, Liu WS, Qin HD, Xu YF, Yu DD, Feng QS, et al. Effect of family history of cancers and environmental factors on risk of nasopharyngeal carcinoma in Guangdong, China. Cancer Epidemiology. 2010;34:419-24.
190 Ruan HL, Xu FH, Liu WS, Feng QS, Chen LZ, Zeng YX, et al. Alcohol and tea consumption in relation to the risk of nasopharyngeal carcinoma in Guangdong, China. Frontiers of Medicine in China. 2010;4:448-56.
191 Ruan HL, Qin HD, Shugart YY, Bei JX, Luo FT, Zeng YX, et al. Developing Genetic Epidemiological Models to Predict Risk for Nasopharyngeal Carcinoma in High-Risk Population of China. PLoS ONE. 2013;8 (2) (no pagination).
192 Shanmugaratnam K, Tye CY, Goh EH, Chia KB. Etiological factors in nasopharyngeal carcinoma: a hospital-based, retrospective, case-control, questionnaire study. In: de-Thé G, Ito Y, editors. Nasopharyngeal Carcinoma:

Etiology and Control: International Agency for Research on Cancer; 1978. p. 199-212.
193 Shao JY, Cao Y, Miao XP, Huang MY, Deng L, Hao JJ, et al. A single nucleotide polymorphism in the matrix metalloproteinase 2 promoter is closely associated with high risk of nasopharyngeal carcinoma in Cantonese from southern China. Chin J Cancer. 2011;30:620-6.
194 Sheng L, Sun X, Zhang L, Su D. ABO blood group and nasopharyngeal carcinoma risk in a population of Southeast China. Int J Cancer. 2013;133:893-7.
195 Shield KD, Ferlay J, Jemal A, Sankaranarayanan R, Chaturvedi AK, Bray F, et al. The global incidence of lip, oral cavity, and pharyngeal cancers by subsite in 2012. Ca-a Cancer Journal for Clinicians. 2017;67:51-64.
196 Shih LC, Tsai CW, Tsai MH, Tsou YA, Chang WS, Li FJ, et al. Association of Cyclin D1 Genotypes with Nasopharyngeal Carcinoma Risk. Anticancer Research. 2012;32:1093-8.
197 Simons MJ, Day NE, Wee GB, Shanmugaratnam K, Ho HC, Wong SH, et al. Nasopharyngeal carcinoma V: immunogenetic studies of Southeast Asian ethnic groups with high and low risk for the tumor. Cancer Res. 1974;34:1192-5.
198 Simons MJ, Wee GB, Day NE, Morris PJ, Shanmugaratnam K, De-The GB. Immunogenetic aspects of nasopharyngeal carcinoma: I. Differences in HL-A antigen profiles between patients and control groups. Int J Cancer. 1974;13:122-34.
199 Simons MJ, Wee GB, Chan SH, Shanmugaratnam K. Probable identification of an HL-A second-locus antigen associated with a high risk of nasopharyngeal carcinoma. Lancet. 1975;1:142-3.
200 Simons MJ, Wee GB, Chan SH, Shanmugaratnam K, Day NE, de-The G. Immunogenetic aspects of nasopharyngeal carcinoma (NPC) III. HL A type as a genetic marker of NPC predisposition to test the hypothesis that Epstein Barr virus is an etiological factor in NPC. IARC (International Agency for Research on Cancer) Scientific Publications. 1975;No.11 II:249-58.
201 Song C, Chen LZ, Zhang RH, Yu XJ, Zeng YX. Functional variant in the 3'-untranslated region of toll-like receptor 4 is associated with nasopharyngeal carcinoma risk. Cancer Biology and Therapy. 2006;5:1285-91.
202 Sousa H, Santos AM, Catarinoa R, Pinto D, Vasconcelos A, Lopes C, et al. Linkage of TP53 codon 72 pro/pro genotype as predictive factor for nasopharyngeal carcinoma development. European Journal of Cancer Prevention. 2006;15:362-6.
203 Sousa H, Pando M, Breda E, Catarino R, Medeiros R. Role of the MDM2 SNP309 polymorphism in the initiation and early age of onset of nasopharyngeal carcinoma. Molecular Carcinogenesis. 2011;50:73-9.
204 Sousa H, Breda E, Santos AM, Catarino R, Pinto D, Medeiros R. Genetic risk markers for nasopharyngeal carcinoma in Portugal: Tumor necrosis factor alpha-308G>A polymorphism. DNA and Cell Biology. 2011;30:99-103.
205 Sousa H, Breda E, Santos AM, Catarino R, Pinto D, Canedo P, et al. IL-1RN VNTR polymorphism as a susceptibility marker for nasopharyngeal carcinoma in Portugal. Archives of Oral Biology. 2013;58:1040-6.
206 Sousa H, Mesquita L, Ribeiro J, Catarino R, Breda E, Medeiros R. Polymorphisms in host immune response associated genes and risk of nasopharyngeal carcinoma development in Portugal. Immunobiology. 2016;221:145-52.
207 Sousa H, Bastos M, Ribeiro J, Oliveira S, Breda E, Catarino R, et al. 5'UTR +24T>C CR2 is not associated with nasopharyngeal carcinoma development in the North Region of Portugal. Oral Diseases. 2016;22:280-4.
208 Sriamporn S, Vatanasapt V, Pisani P, Yongchaiyudha S, Rungpitarangsri V. Environmental risk factors for nasopharyngeal carcinoma: a case-control study in northeastern Thailand. Cancer Epidemiol Biomarkers Prev. 1992;1:345-8.
209 Su WH, Tse KP, Yang ML, Shugart YY, Chang YS. Copy number variation analysis identified multiple genetic variants related to nasopharyngeal carcinoma

predisposition. Cancer Research Conference: 102nd Annual Meeting of the American Association for Cancer Research, AACR. 2011;71.

210 Sui J, Mo Q, Li XJ, Ma J, Ren YX, Gao W, et al. [Transporter associated with antigen processing 1 637A/G gene polymorphisms and susceptibility to nasopharyngeal carcinoma in Han population in Yunnan Province, China]. [Chinese]. Zhonghua er bi yan hou tou jing wai ke za zhi = Chinese journal of otorhinolaryngology head and neck surgery. 2010;45:238-43.

211 Tai SH, Wei YS, Wang P, Zhou B, Ran P, Yang ZH, et al. [Genetic polymorphisms of interferon-gamma and interleukin-8 in patients with nasopharyngeal carcinoma]. Sichuan Da Xue Xue Bao Yi Xue Ban. 2007;38:862-5.

212 Tang M, Zeng Y, Poisson A, Marti D, Guan L, Zheng Y, et al. Haplotype-dependent HLA susceptibility to nasopharyngeal carcinoma in a Southern Chinese population. Genes Immun. 2010;11:334-42.

213 Tang M, Lautenberger JA, Gao X, Sezgin E, Hendrickson SL, Troyer JL, et al. The Principal Genetic Determinants for Nasopharyngeal Carcinoma in China Involve the HLA Class I Antigen Recognition Groove. PLoS Genetics. 2012;8 (11) (no pagination).

214 Tian W, Zeng XM, Li LX, Jin HK, Luo QZ, Wang F, et al. Gender-specific associations between MICA-STR and nasopharyngeal carcinoma in a southern Chinese Han population. Immunogenetics. 2006;58:113-21.

215 Tiwawech D, Srivatanakul P, Karaluk A, Ishida T. The p53 codon 72 polymorphism in Thai nasopharyngeal carcinoma. Cancer Letters. 2003;198:69-75.

216 Tiwawech D, Srivatanakul P, Karalak A, Ishida T. Glutathione S-transferase M1 gene polymorphism in Thai nasopharyngeal carcinoma. Asian Pac J Cancer Prev. 2005;6:270-5.

217 Tsai CW, Tsai MH, Shih LC, Chang WS, Lin CC, Bau DT. Association of Interleukin-10 (IL10) Promoter Genotypes with Nasopharyngeal Carcinoma Risk in Taiwan. Anticancer Research. 2013;33:3391-6.

218 Tse KP, Su WH, Chang KP, Tsang NM, Yu CJ, Tang P, et al. Genome-wide Association Study Reveals Multiple Nasopharyngeal Carcinoma-Associated Loci within the HLA Region at Chromosome 6p21.3. American Journal of Human Genetics. 2009;85:194-203.

219 Tsou YA, Tsai CW, Tsai MH, Chang WS, Li FJ, Liu YF, et al. Association of Caveolin-1 Genotypes with Nasopharyngeal Carcinoma Susceptibility in Taiwan. Anticancer Research. 2011;31:3629-32.

220 Turkoz FP, Celenkoglu G, Dogu GG, Kalender ME, Coskun U, Alkis N, et al. Risk factors of nasopharyngeal carcinoma in Turkey-an epidemiological survey of the Anatolian Society of Medical Oncology. Asian Pac J Cancer Prev. 2011;12:3017-21.

221 Ung A, Chen CJ, Levine PH, Cheng YJ, Brinton LA, Chen IH, et al. Familial and sporadic cases of nasopharyngeal carcinoma in Taiwan. Anticancer Research. 1999;19:661-5.

222 Vaughan TL, Shapiro JA, Burt RD, Swanson GM, Berwick M, Lynch CF, et al. Nasopharyngeal cancer in a low-risk population: Defining risk factors by histological type. Cancer Epidemiology Biomarkers and Prevention. 1996;5:587-93.

223 Vaughan TL, Stewart PA, Teschke K, Lynch CF, Swanson GM, Lyon JL, et al. Occupational exposure to formaldehyde and wood dust and nasopharyngeal carcinoma. Occup Environ Med. 2000;57:376-84.

224 Wang T, Hu K, Ren JH, Zhu QY, Wu G, Peng G. Polymorphism of VEGF-2578C/A associated with the risk and aggressiveness of nasopharyngeal carcinoma in a Chinese population. Molecular Biology Reports. 2010;37:59-65.

225 Ward MH, Pan WH, Cheng YJ, Li FH, Brinton LA, Chen CJ, et al. Dietary exposure to nitrite and nitrosamines and risk of nasopharyngeal carcinoma in Taiwan. Int J Cancer. 2000;86:603-9.

226 Wei YS, Lan Y, Tang RG, Xu QQ, Huang Y, Nong HB, et al. Single nucleotide polymorphism and haplotype association of the interleukin-8 gene with nasopharyngeal carcinoma. Clin Immunol. 2007;125:309-17.
227 Wei YS, Kuang XH, Zhu YH, Liang WB, Yang ZH, Tai SH, et al. Interleukin-10 gene promoter polymorphisms and the risk of nasopharyngeal carcinoma. Tissue Antigens. 2007;70:12-7.
228 Wei YS, Zhu YH, Du B, Yang ZH, Liang WB, Lv ML, et al. Association of transforming growth factor-beta1 gene polymorphisms with genetic susceptibility to nasopharyngeal carcinoma. Clin Chim Acta. 2007;380:165-9.
229 Wei YS, Lan Y, Luo B, Lu D, Nong HB. Association of variants in the interleukin-27 and interleukin-12 gene with nasopharyngeal carcinoma. Mol Carcinog. 2009;48:751-7.
230 Wei YS, Lan Y, Zhang LA, Wang JC. Association of the Interleukin-2 Polymorphisms with Interleukin-2 Serum Levels and Risk of Nasopharyngeal Carcinoma. DNA and Cell Biology. 2010;29:363-8.
231 Wei Y, Long X, Liu Z, Ma Y, Deng Z. Genetic polymorphism of glutathione-S-transferase M1 and T1 in associated with carcinogenesis of hepatocellular carcinoma and nasopharyngeal carcinoma. Chinese-German Journal of Clinical Oncology. 2012;11:138-41.
232 West S, Hildesheim A, Dosemeci M. Non-viral risk factors for nasopharyngeal carcinoma in the Philippines: results from a case-control study. Int J Cancer. 1993;55:722-7.
233 Wu SB, Hwang SJ, Chang AS, Hsieh T, Hsu MM, Hsieh RP, et al. Human leukocyte antigen (HLA) frequency among patients with nasopharyngeal carcinoma in Taiwan. Anticancer Res. 1989;9:1649-53.
234 Xiao M, Qi F, Chen X, Luo Z, Zhang L, Zheng C, et al. Functional polymorphism of cytotoxic T-lymphocyte antigen 4 and nasopharyngeal carcinoma susceptibility in a Chinese population. Int J Immunogenet. 2010;37:27-32.
235 Xiao M, Zhang L, Zhu X, Huang J, Jiang H, Hu S, et al. Genetic polymorphisms of MDM2 and TP53 genes are associated with risk of nasopharyngeal carcinoma in a Chinese population. BMC Cancer. 2010;10:147.
236 Xiao M, Hu S, Zhang L, Huang J, Jiang H, Cai X. Polymorphisms of CD44 gene and nasopharyngeal carcinoma susceptibility in a Chinese population. Mutagenesis. 2013;28:577-82.
237 Xie SH, Yu ITS, Tse LA, Au JSK, Lau JSM. Tobacco smoking, family history, and the risk of nasopharyngeal carcinoma: a case-referent study in Hong Kong Chinese. Cancer Causes & Control. 2015;26:913-21.
238 Xiong W, Zeng ZY, Xia JH, Xia K, Shen SR, Li XL, et al. A susceptibility locus at chromosome 3p21 linked to familial nasopharyngeal carcinoma. Cancer Res. 2004;64:1972-4.
239 Xu YF, Liu WL, Dong JQ, Liu WS, Feng QS, Chen LZ, et al. Sequencing of DC-SIGN promoter indicates an association between promoter variation and risk of nasopharyngeal carcinoma in cantonese. Bmc Medical Genetics. 2010;11.
240 Xu FH, Xiong D, Xu YF, Cao SM, Xue WQ, Qin HD, et al. An epidemiological and molecular study of the relationship between smoking, risk of nasopharyngeal carcinoma, and epsteinbarr virus activation. Journal of the National Cancer Institute. 2012;104:1396-410.
241 Yang X, Diehl S, Pfeiffer R, Chen CJ, Hsu WL, Dosemeci M, et al. Evaluation of risk factors for nasopharyngeal carcinoma in high-risk nasopharyngeal carcinoma families in Taiwan. Cancer Epidemiology Biomarkers and Prevention. 2005;14:900-5.
242 Yang ZH, Du B, Wei YS, Zhang JH, Zhou B, Liang WB, et al. Genetic polymorphisms of the DNA repair gene and risk of nasopharyngeal carcinoma. DNA Cell Biol. 2007;26:491-6.

243 Yang ZH, Liang WB, Jia J, Wei YS, Zhou B, Zhang L. The xeroderma pigmentosum group C gene polymorphisms and genetic susceptibility of nasopharyngeal carcinoma. Acta Oncol. 2008;47:379-84.
244 Yang ZH, Dai Q, Kong XL, Yang WL, Zhang L. Association of ERCC1 polymorphisms and susceptibility to nasopharyngeal carcinoma. Mol Carcinog. 2009;48:196-201.
245 Yang ZH, Dai Q, Zhong L, Zhang X, Guo QX, Li SN. Association of IL-1 polymorphisms and IL-1 serum levels with susceptibility to nasopharyngeal carcinoma. Mol Carcinog. 2011;50:208-14.
246 Yang L, Liu B, Qiu F, Huang B, Li Y, Huang D, et al. The effect of functional MAPKAPK2 copy number variation CNV-30450 on elevating nasopharyngeal carcinoma risk is modulated by EBV infection. Carcinogenesis. 2014;35:46-52.
247 Ye YF, Xiang YQ, Fang F, Gao R, Zhang LF, Xie SH, et al. Hepatitis B Virus Infection and Risk of Nasopharyngeal Carcinoma in Southern China. Cancer Epidemiology Biomarkers & Prevention. 2015;24:1766-73.
248 Yew P-Y, Mushiroda T, Kiyotani K, Govindasamy GP, Yap L-F, Teo S-H, et al. Identification of a functional variant in SPLUNC1 associated with nasopharyngeal carcinoma susceptibility among Malaysian Chinese. Molecular Carcinogenesis. 2012;51:E74-E82.
249 Yu MC, Ho JH, Lai SH, Henderson BE. Cantonese-style salted fish as a cause of nasopharyngeal carcinoma: report of a case-control study in Hong Kong. Cancer Res. 1986;46:956-61.
250 Yu MC, Mo CC, Chong WX, Yeh FS, Henderson BE. Preserved foods and nasopharyngeal carcinoma: a case-control study in Guangxi, China. Cancer Res. 1988;48:1954-9.
251 Yu MC, Huang TB, Henderson BE. Diet and nasopharyngeal carcinoma: a case-control study in Guangzhou, China. Int J Cancer. 1989;43:1077-82.
252 Yu MC, Garabrant DH, Huang TB, Henderson BE. Occupational and other non-dietary risk factors for nasopharyngeal carcinoma in Guangzhou, China. Int J Cancer. 1990;45:1033-9.
253 Yu KJ, Gao XJ, Chen CJ, Yang XH, Diehl SR, Goldstein A, et al. Association of human leukocyte antigens with nasopharyngeal carcinoma in high-risk multiplex families in Taiwan. Human Immunology. 2009;70:910-4.
254 Yuan JM, Wang XL, Xiang YB, Gao YT, Ross RK, Yu MC. Non-dietary risk factors for nasopharyngeal carcinoma in Shanghai, China. Int J Cancer. 2000;85:364-9.
255 Zeng FF, Liu YT, Lin XL, Fan YY, Zhang XL, Xu CH, et al. Folate, vitamin B-6, vitamin B-12 and methionine intakes and risk for nasopharyngeal carcinoma in Chinese adults: a matched case-control study. British Journal of Nutrition. 2016;115:121-8.
256 Zhao M, Cai H, Zheng H, Yang X, Fang W, Zhang L, et al. Further evidence for the existence of major susceptibility of nasopharyngeal carcinoma in the region near HLA-A locus in Southern Chinese. Journal of Translational Medicine. 2012;10 (1) (no pagination).
257 Zheng YM, Tuppin P, Hubert A, Jeannel D, Pan YJ, Zeng Y, et al. ENVIRONMENTAL AND DIETARY RISK-FACTORS FOR NASOPHARYNGEAL CARCINOMA - A CASE-CONTROL STUDY IN ZANGWU COUNTY, GUANGXI, CHINA. Br J Cancer. 1994;69:508-14.
258 Zheng X, Yan L, Nilsson B, Eklund G, Drettner B. Epstein-Barr virus infection, salted fish and nasopharyngeal carcinoma. A case-control study in Southern China. Acta Oncologica. 1994;33:867-72.
259 Zheng MZ, Qin HD, Yu XJ, Zhang RH, Chen LZ, Feng QS, et al. Haplotype of gene Nedd4 binding protein 2 associated with sporadic nasopharyngeal carcinoma in the Southern Chinese population. J Transl Med. 2007;5:36.

260 Zheng J, Zhang C, Jiang L, You YH, Liu YH, Lu JC, et al. Functional NBS1 Polymorphism Is Associated With Occurrence and Advanced Disease Status of Nasopharyngeal Carcinoma. Molecular Carcinogenesis. 2011;50:689-96.
261 Zheng J, Liu B, Zhang LY, Jiang L, Huang BF, You YH, et al. The protective role of polymorphism MKK4-1304 T > G in nasopharyngeal carcinoma is modulated by Epstein-Barr virus' infection status. International Journal of Cancer. 2012;130:1981-90.
262 Zheng H, Dai W, Tang C, Cheung A, Ko J, Sham P, et al. Unraveling the genetic basis of nasopharyngeal carcinoma using next-generation sequencing approaches. Cancer Research Conference: 106th Annual Meeting of the American Association for Cancer Research, AACR. 2015;75.
263 Zhou XX, Jia WH, Shen GP, Qin HD, Yu XJ, Chen LZ, et al. Sequence variants in toll-like receptor 10 are associated with nasopharyngeal carcinoma risk. Cancer Epidemiology Biomarkers & Prevention. 2006;15:862-6.
264 Zhou G, Zhai Y, Cui Y, Zhang X, Dong X, Yang H, et al. MDM2 promoter SNP309 is associated with risk of occurrence and advanced lymph node metastasis of nasopharyngeal carcinoma in Chinese population. Clin Cancer Res. 2007;13:2627-33.
265 Zhou G, Zhai Y, Cui Y, Qiu W, Yang H, Zhang X, et al. Functional polymorphisms and haplotypes in the promoter of the MMP2 gene are associated with risk of nasopharyngeal carcinoma. Hum Mutat. 2007;28:1091-7.
266 Zhou B, Rao L, Li Y, Gao LB, Wang YY, Chen Y, et al. A functional insertion/deletion polymorphism in the promoter region of NFKB1 gene increases susceptibility for nasopharyngeal carcinoma. Cancer Letters. 2009;275:72-6.
267 Zhu XN, Chen R, Kong FH, Liu W. Human leukocyte antigens -A, -B, -C, and -DR and nasopharyngeal carcinoma in northern China. Ann Otol Rhinol Laryngol. 1990;99:286-7.
268 Zhu KM, Levine RS, Brann EA, Gnepp DR, Baum MK. A POPULATION-BASED CASE-CONTROL STUDY OF THE RELATIONSHIP BETWEEN CIGARETTE-SMOKING AND NASOPHARYNGEAL CANCER (UNITED-STATES). Cancer Causes & Control. 1995;6:507-12.
269 Zhu Y, Xu Y, Wei Y, Liang W, Liao M, Zhang L. Association of IL-1B gene polymorphisms with nasopharyngeal carcinoma in a Chinese population. Clin Oncol (R Coll Radiol). 2008;20:207-11.
270 Zhu Q, Wang T, Ren J, Hu K, Liu W, Wu G. FAS-670A/G polymorphism: A biomarker for the metastasis of nasopharyngeal carcinoma in a Chinese population. Clin Chim Acta. 2010;411:179-83.
271 Zong YS, Sham JST, Ng MH, Ou XT, Guo YQ, Zheng SA, et al. IMMUNOGLOBULIN-A AGAINST VIRAL CAPSID ANTIGEN OF EPSTEIN-BARR-VIRUS AND INDIRECT MIRROR EXAMINATION OF THE NASOPHARYNX IN THE DETECTION OF ASYMPTOMATIC NASOPHARYNGEAL CARCINOMA. Cancer. 1992;69:3-7.
